# Revealing the Effect of Stereocontrol on Intermolecular Interactions between Abiotic, Sequence-Defined Polyurethanes and a Ligand

**DOI:** 10.1021/acsbiomaterials.4c00456

**Published:** 2024-05-28

**Authors:** Maksymilian Szatko, Weronika Forysiak, Sara Kozub, Tadeusz Andruniów, Roza Szweda

**Affiliations:** † Łukasiewicz Research Network—PORT Polish Center for Technology Development, Stabłowicka 147, 54-066 Wroclaw, Poland; ‡ Department of Chemistry, Wrocław University of Science and Technology, Wybrzeże Wyspiańskiego 27, 50-370 Wroclaw, Poland; § Faculty of Chemistry, University of Wrocław, F. Joliot-Curie 14, 50-383 Wrocław, Poland; ∥ Center for Advanced Technologies, Adam Mickiewicz University, Uniwersytetu Poznańskiego 8, 61-614 Poznan, Poland

**Keywords:** stereocontrolled polymers, sequence-defined polymers, functional macromolecules, sensing, detection of bisphenol A, molecular dynamics, structure simulations

## Abstract

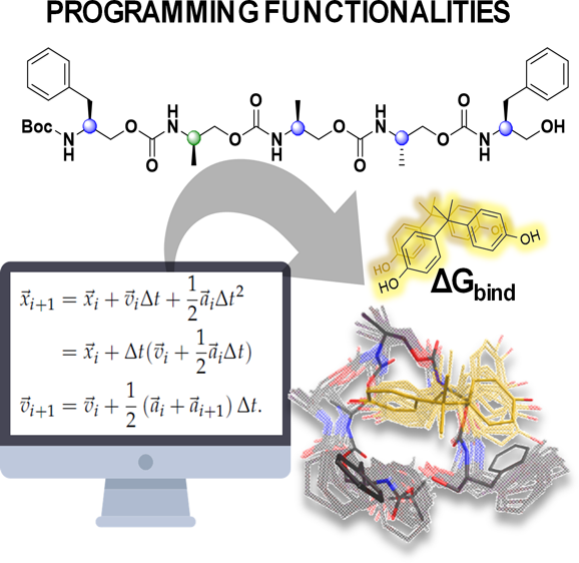

The development of precision polymer synthesis has facilitated access to a diverse library of abiotic structures wherein chiral monomers are positioned at specific locations within macromolecular chains. These structures are anticipated to exhibit folding characteristics similar to those of biotic macromolecules and possess comparable functionalities. However, the extensive sequence space and numerous variables make selecting a sequence with the desired function challenging. Therefore, revealing sequence–function dependencies and developing practical tools are necessary to analyze their conformations and molecular interactions. In this study, we investigate the effect of stereochemistry, which dictates the spatial location of backbone and pendant groups, on the interaction between sequence-defined oligourethanes and bisphenol A ligands. Various methods are explored to analyze the receptor-like properties of model oligomers and the ligand. The accuracy of molecular dynamics simulations and experimental techniques is assessed to uncover the impact of discrete changes in stereochemical arrangements on the structures of the resulting complexes and their binding strengths. Detailed computational investigations providing atomistic details show that the formed complexes demonstrate significant structural diversity depending on the sequence of stereocenters, thus affecting the oligomer–ligand binding strength. Among the tested techniques, the fluorescence spectroscopy data, fitted to the Stern–Volmer equation, are consistently aligned with the calculations, thus validating the developed simulation methodology. The developed methodology opens a way to engineer the structure of sequence-defined oligomers with receptor-like functionality to explore their practical applications, e.g., as sensory materials.

## 1 Introduction

The sequence-defined polymer term was recently formulated to describe a macromolecular species whose length is discrete and the order of monomeric units within a chain is strictly specific.^1−4^ Archetypical representatives of these chemical species are naturally occurring polymers, i.e., proteins and nucleic acids. Non-natural building blocks and backbones broaden the scope of macromolecular structures thanks to advancements in precision polymer synthesis.^5−8^ Backbone structures may range from bioinspired peptoids^9,10^ or polyphosphates^11^ to novel chemical moieties, i.e., triazoles,^12−14^ urease,^15^ urethanes,^16−19^ ethers,^20,21^ esters,^22,23^ or π-conjugated oligomers.^24−27^ Applying chiral monomers enables full control over the stereochemistry of macromolecules.^12,18,28−31^ Consequently, primary structure control and stereochemistry are emerging as promising tools to induce functionalities in abiotic macromolecules.^32^

Discrete, abiotic macromolecules have already been proven to display functions such as natural polymers. For instance, sequence-defined polymers are used as binary information carriers similar to DNA storing genetic code.^33−36^ The encoded digital data can be revealed by mass spectrometry^37−39^ or nanopore sequencing.^40^ Interestingly, a relevant design of the abiotic polymer structure enables the editing of encoded information by the light trigger.^41^ Besides data storage, such macromolecules have been used in catalysis,^42,43^ drug delivery,^44−48^ sensing,^27,49−51^ selective binding,^52,53^ molecular transport,^47,54^ or as peptidomimetic foldamers.^55,56^ The biological environment, where natural macromolecules perform sophisticated functions, is chiral. Therefore, the stereochemistry of macromolecules should be an essential parameter for their function. However, the effect of the stereochemistry on polymer function has not been widely explored.

In general, the functionalities of biological macromolecules are derived from their ability to arrange their chains spatially, attaining a three-dimensional (3D) structure. It is dictated by a sequence of monomers characterized by various pendant substituents and stereochemistry. Stereocontrol opens up the possibility to influence the abiotic macromolecular interactions with the chiral, biological environment.^53,57,58^ Stereospecificity, in combination with monomer sequence control, offers a wide library of abiotic structures to engineer functional macromolecules with a broad range of chemical and physical properties. However, very little is known about the sequence–structure–function relationship of abiotic macromolecules, particularly those built on nonamide backbones. Therefore, engineering selective functionalities into abiotic polymers remains beyond reach due to the enormous sequence space generating multiple variables that impede the rational structure design until effective tools for characterizing their functionalities become available. The lack of a precise methodology impedes the characterization of their structural properties and the assessment of their functionality. While taking cues from related protocols developed in biosciences is possible, the intricate nature of studying nuanced properties, both structural and functional, often renders the direct transfer of these methods challenging.

Here, the impact of sequence stereospecificity on receptor-like functionalities of model oligourethanes toward the target molecule bisphenol A (BPA) was investigated by computational and experimental methods. BPA and natural [3*H*]estradiol compete in the binding process to the estrogen receptors; therefore, it is an endocrine-disruptive substance and should be monitored in the environment.^59,60^ The verification of molecular dynamic simulation outcomes by experimental techniques contributed to the development of a precise computational tool for the characterization of receptor-like functionalities of non-natural, sequence-defined oligourethanes. The developed methodology opens a way to engineer the structure of sequence-defined oligomers with receptor-like functionality to explore their practical applications, e.g., as sensory materials.

## 2 Results and Discussion

**Figure 1:**
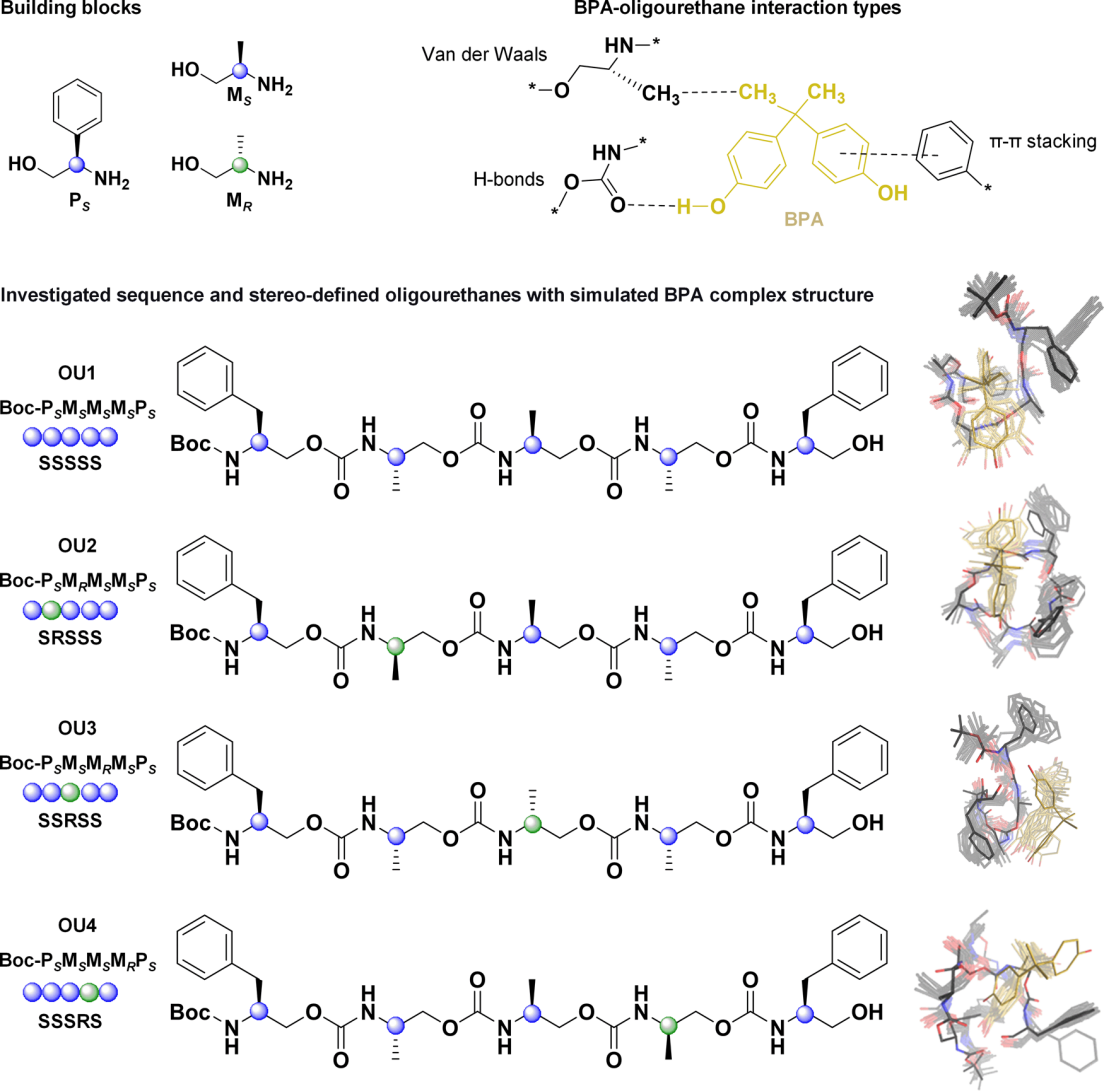
In this study, oligomers consist of aromatic (*P*_S_) and aliphatic (*M*_S_, *M*_R_) monomers with a defined stereochemistry. Structures are designed to foster attractive interactions with BPA, encompassing H-bonds, van der Waals, and π–π stacking forces. The depicted collection of discrete oligourethanes OU1–OU4 contains mutation of one stereocenter in various positions. Structures of oligourethane–BPA complexes are received from the multiple simulated annealing molecular dynamics (MSA-MD). For clarity, all hydrogens have been hidden, and nitrogen and oxygen are represented by blue and red, respectively. BPA atoms are colored yellow.

**Figure 2:**
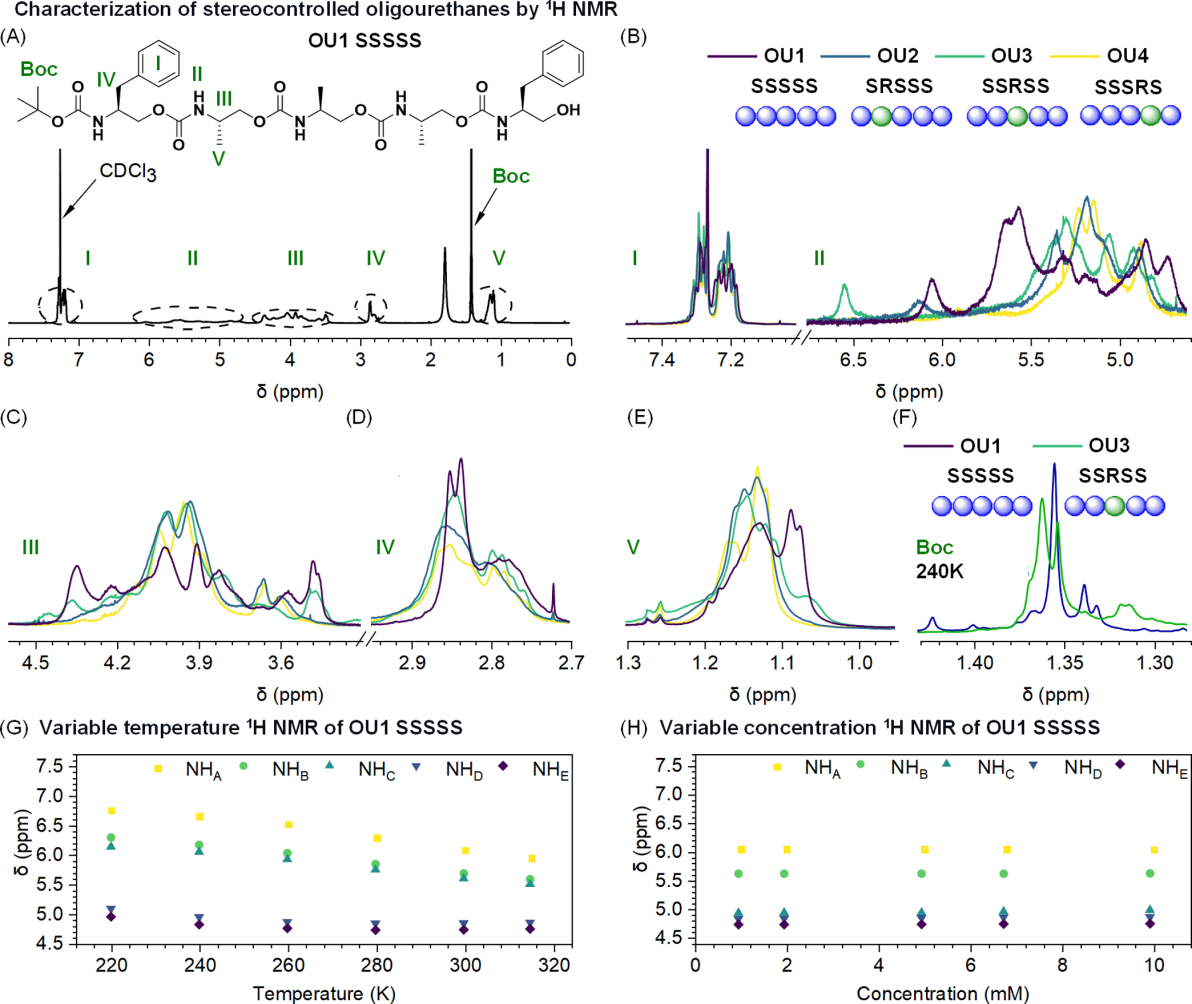
^1^H NMR characterization of stereocontrolled oligourethanes revealed differences between oligomers depending on the sequence of stereocenters. (A) Representative ^1^H NMR spectrum of OU1 SSSSS. Upon zooming individual regions, I (H–Ar, B), II (H–N, B), III (backbone protons, C), IV (–CH_2_–Ar, D), and V (–CH_3_, E), spectrum shape dependence on the stereochemical sequence for OU1–OU4 with one stereocenter mutation becomes apparent. Sequence-specific splitting of the Boc signal is noticed, indicating distinct conformational preferences of studied oligomers (F). Variable temperature experiments (6.8 mM) indicate the formation of intramolecular hydrogen bonds (G). A notable chemical shift in N–H protons upon temperature decrease is evident, where no change occurs during the variable concentration experiment in the range of 1–10 mM (H), confirming that the hydrogen bonds come from the single-chain folding.

A library of model oligourethanes with one (OU1–OU4, Figure 1) or two mutations (OU5–OU7, Figure S1) of stereocenters was evaluated toward an ability to bind the BPA ligand. Following the peptidomimetic nomenclature, studied molecules may be considered γ-peptide derivatives.^61−63^ The resonance stabilization of the urethane bond tends to foster a planar conformation,^64^ which promotes the folding of those scaffolds. Therefore, we hypothesized that oligourethane scaffolds built from chiral monomers can attain a specific set of shapes in solution, depending on the arrangement of stereochemically distinct monomers that affect their interactions with the ligand. Oligourethane sequences with methyl and benzyl pendant substituents are assumed to exhibit attractive interactions with the BPA (Figure 2). BPA is a symmetrical molecule composed of two phenol rings connected via a tetrahedral carbon with two methyl groups. Therefore, BPA is expected to form hydrogen bonds with urethane backbone groups and π–π stacking and van der Waals (vdW) interactions with oligomer side groups.

### 2.1 Characterization of Oligourethanes

Investigated stereocontrolled, discrete oligourethanes (OU1–OU7, Figure S1) were synthesized according to the solution synthesis protocol using chiral monomers (*P*_S_, *M*_S_, *M*_R_, Figure 1) as described previously.^18^ The structures of products were confirmed by size-exclusion chromatography (SEC) (Figures S2–S8), liquid chromatography–mass spectrometry (LC–MS) (Figures S9–S15), and ^1^H NMR (Figures S16–S22) analyses. For all oligomers, SEC analyses yielded a single, narrow signal, proving the uniform structure of oligomers. Overlapped SEC chromatograms show that each oligomer is characterized by a specific retention time that indicates the stereochemistry-dependent hydrodynamic volume of oligomer chains (Figure S8B). In LC–MS chromatograms, we observed one peak corresponding to the oligomer molar mass. As expected, for all studied diastereoisomers, we observed three main signals at *m*/*z* 632.33, 732.38, and 754.36 corresponding to ions [M - Boc + H]^+^, [M + H]^+^, and [M + Na]^+^, respectively.

^1^H NMR analyses revealed structural differences between oligomers depending on the sequence of stereocenters indicating various spatial conformation preferences (Figures 2A–F and S16–S22). In the spectra, we distinguished six signal regions that come from aromatic protons at 7.0–7.5 ppm (I), urethane N–H at 4.6–6.75 ppm (II), backbone protons at 3.5–4.5 ppm (III), –CH_2_– from the benzyl side chain at 2.5–3.0 ppm (IV), methyl side chains at 1–1.2 ppm (V), and Boc protons at 1.40 ppm (Boc), as presented in Figure 2A. At room temperature, protons from backbone and methyl side chains occur as broadened multiplets with sequence-specific patterns (Figure 2B–F) defined by a diverse spatial arrangement of chiral building blocks. The sequence conformation stability can be assessed based on the Boc ^1^H NMR signal as an internal probe (experiment at 240 K), which delivers information about the homogeneity of the Boc neighborhood reflected in signal splitting (Figure 2F).

The decrease in temperature improves the resolution of the signals, causing the stabilization of conformation and formation of energetically favored structures (representative ^1^H NMR variable temperature spectra for OU1 and OU2; see Figures S23 and S24). The decreased temperature has diminished the exchange rate between conformations of oligourethanes, highlighting the uniqueness of shapes for each stereosequence. Moreover, cooling causes significant alterations of urethane N–H proton chemical shifts, indicating the presence of intramolecular hydrogen bonds (Figures 2G, S24, and S26).^65,66^ The possibility of intermolecular hydrogen bond formation due to the aggregation of macromolecules was disproved through variable concentration ^1^H NMR experiments in the range of 1–10 mM. Upon changes in concentration, values of N–H chemical shifts remain constant. Therefore, the obtained data suggest that within the studied concentration range, we observe intramolecular hydrogen bonds from a single-chain folding (Figures 2H, S25, and S26). All investigated oligomers display conformational preferences, and their conformations depend on the sequence of stereocenters; therefore, each oligomer represents a unique set of shapes in the solution. Further, we examined how stereocenter mutations affect the formation of oligourethane–BPA complexes to assess the impact of stereochemistry on their receptor-like function.

### 2.2 Studies of the Oligourethane–BPA Interactions by Molecular Dynamics

**Figure 3:**
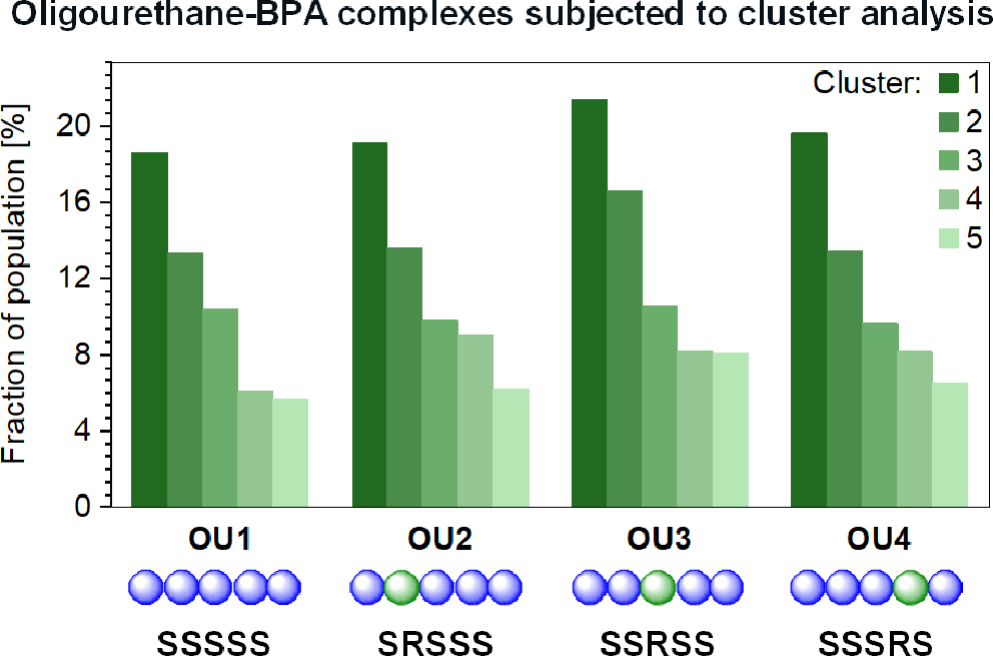
Representative cluster distributions for oligourethanes with a single stereocenter mutation, OU1–OU4, complexed with the BPA. The cluster analysis was performed based on the structural similarity of BPA–oligomer complexes.

Molecular dynamics simulation studies revealed details of oligourethane–BPA structures. Extensive MSA-MD computational protocols followed by clustering were employed to characterize the conformational space of the investigated oligourethane–BPA complexes solvated in implicit chloroform. Simulated annealing was performed for 150 ps by heating from 298 to 500 K, equilibrating, and finally reducing the temperature to 0 K. These calculations were repeated 300 times with randomly generated velocities using the Maxwell–Boltzmann distribution. The applied procedure generates a set of random local minima, which were the starting configurations for 10 ns MD simulations at 298 K. In all simulations, the Amber14SB^67^ force field parameters were employed for oligourethane–BPA complexes. More technical details on simulation parameters are provided in the Supporting Information (Section 4.2). Structures derived from the trajectories of the oligourethane–BPA complex were organized into clusters based on the structural resemblance, corresponding to a specific conformation. Out of 42 866 structures representing the whole ensemble, the 5 most populated clusters were selected and analyzed from a structural standpoint for each oligomer OU1–OU7 (Figure S27). The depicted 5 clusters represent, on average, 60% of the ensemble, whereas a dominant cluster characterizes ca. 20% of all conformations. Representative cluster distributions for oligourethane–BPA complexes with one stereocenter mutation are shown in Figure 3. Simulated three-dimensional structures of oligomer–BPA complexes for dominant clusters are visualized in Figure 1. It is seen that the structure of the complexes depends solely on the stereoconfiguration of the oligomer. The 3D structure of oligourethane with an associated BPA varies among the clusters, inferring that the complexes exhibit conformational flexibility. Oligomer chains adopt diverse shapes to which BPA binds, generating a range of complex conformations that undergo an interchange.

**Figure 4:**
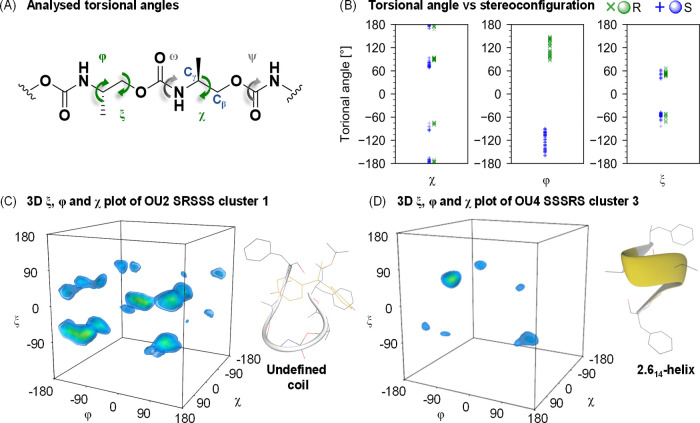
Shapes of oligourethane–BPA complexes can be depicted through graphical analysis of their torsional angles, φ, ξ, and χ. (A) The fragment of a urethane chain shows the projected bond rotations, defining individual torsional angles. (B) The most probable φ, ξ, and χ angle values plotted with respect to the stereochemistry visualize apparent torsion preferences. The analysis involved deriving the highest probability angle values for each monomer within every cluster while considering their stereochemistry. By assessing all mutual angle dependencies via the 3D torsional plot, an example of angle distributions for conformations with respective to the undefined coil (C) or right-handed helix structure (D) is revealed and depicted in the NewCartoon style.

Backbone torsional angles are used to analyze conformations of oligourethane–BPA complexes, identifying a sequence-shape dependency. Structural biology uses the Ramachandran method to graphically analyze the rotation of peptide bonds in the protein chain. Due to steric hindrances and hydrogen bonds, allowed and disallowed torsional angle regions are created, impeding rotations. Their analysis enables one to assess the shape and stability of the three-dimensional structure of the macromolecule. Since urethanes can be considered to be peptide bond relative, the Ramachandran plot methodology was modified to investigate abiotic oligourethanes. We used nomenclature, which refers to the initially developed for peptide bond analysis and defined ψ (C–O), ω (C–N), and φ (N–C_γ_) torsions. In the case of urethanes, the backbone is extended by two rotatable bonds, which obliges us to also introduce ξ (C_γ_–C_β_) and χ (C_β_–O) torsions (Figure 4A). Yet, not all torsions are valid for the analysis; i.e., C–O and C–N bonds, in the urethane group, are affected by electron resonance, thus making ψ and ω torsions immobile and making urethane groups stiff and planar. When the remaining rotations φ, ξ, and χ are analyzed separately, distinct torsional preferences within these systems are unveiled.

We observed that the φ dihedral value is dependent on the stereochemistry of the Cγ atom. The data show that its values are distributed around 120 or −120°, depending solely on the stereochemistry of the introduced monomer. The φ torsion value is <0 if the monomeric unit is of S configuration, and otherwise, φ assumes >0 values if the configuration is R (Figure 4B), whereas no effect of stereoconfiguration is observed for two other torsions ξ and χ. The ξ torsion values oscillate around 60 or −60°, disregarding the stereochemistry. The third torsion χ assumes 4 possible values in proximity to −180, −70, 70, or 180°; similarly, no stereochemical effect has been identified.

Analyzing three angles collectively offers insights into their mutual presence and enables extraction of the structural information. Every set of clusters is composed of a unique distribution of φ, ξ, and χ angles, which reflects the multitude of chain arrangements and possible interaction sites to which BPA binds. Most of the torsional plots for oligourethane–BPA complexes are characterized by multiple, widely distributed values, which reflect undefined coil arrangements, far from helix and sheet characteristics for biomacromolecules (e.g., OU2 SRSSS—cluster 1, Figure 4C; for other examples, see Figures S30 and S32). Among analyzed clusters, we found examples of structural regularity indicating the presence of a helical structure (OU4 SSSRS cluster 3 and OU6 SRSRS cluster 5 depicted in Figures S29 and S31, respectively). For example, cluster 3 of OU4 SSSRS folds into a misshaped right-handed helix (Figure 4D), which occurs 9.6% of the simulated time. The helix structure depends on interactions between monomers in the chain and BPA ligand, which may act as a structure-disrupting agent, thus influencing the stability and regularity of the formed complex. The clusters represent groups of similar complex conformations, not isolated oligomers; hence, a lack of structural regularity is expected.

**Figure 5:**
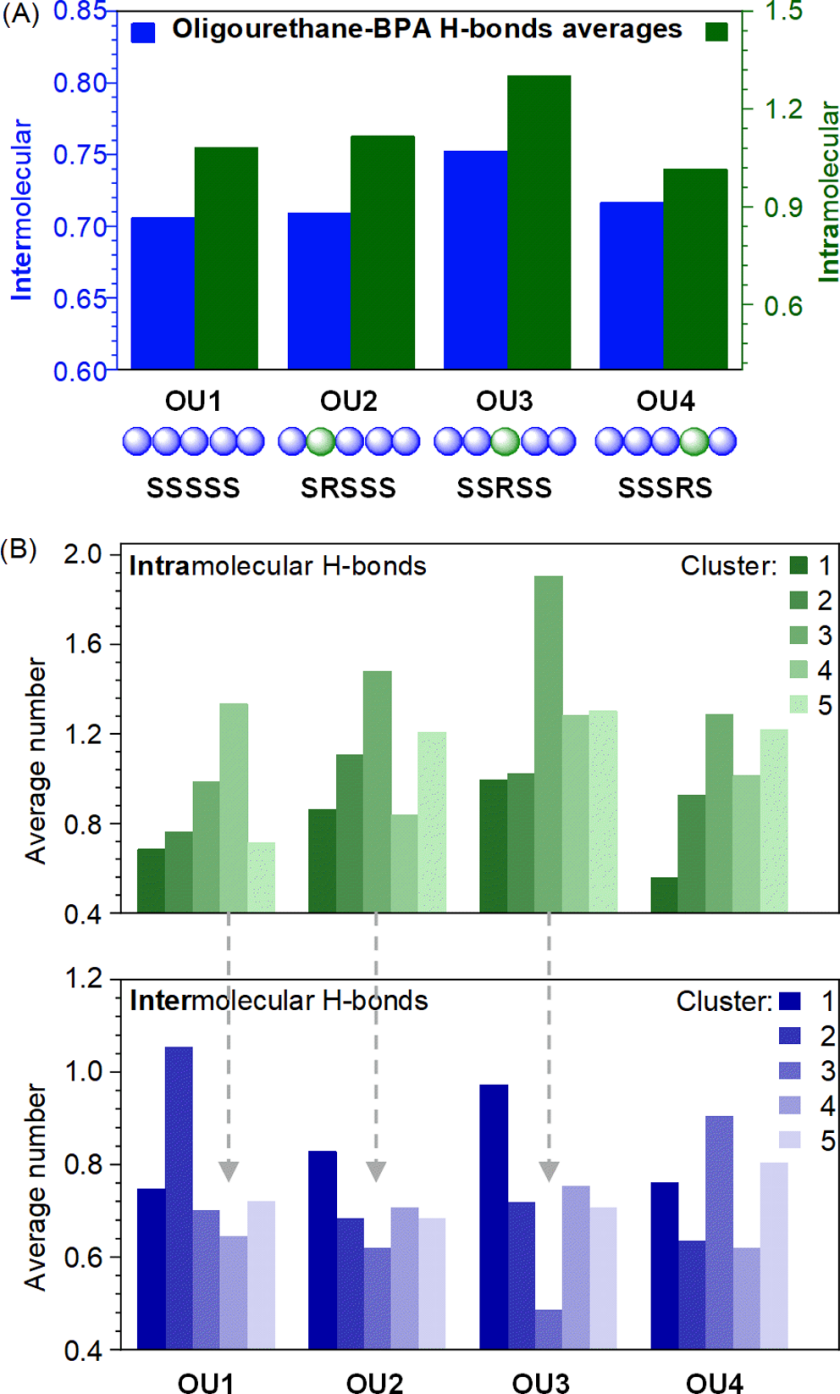
Ensemble average of both intra- and intermolecular hydrogen bonds reveals stereochemistry-dependent characteristics. (A) An inversion of the stereocenter at the third position in the oligomer chain results in an increased count of hydrogen bonds of both types (analysis of the whole ensemble). (B) The analysis of hydrogen bond number averages by individual clusters suggests an inverse tendency of the inter–intra-hydrogen bond formation.

To look into the structural peculiarities of the complex, we studied the formed hydrogen-bonding network. An analysis of intra- and intermolecular hydrogen bonds representing electrostatic interactions in the oligourethane–BPA complexes is presented in Figure 5. The ensemble average analysis provided a general perspective on data analysis (Figure 5A). We see that in complex formation, one hydrogen bond is formed intramolecularly by oligomer and one is used to couple with BPA. The input of H-bonds differs between oligomers. Specifically, when the chirality mutation from S to R occurs at the third position in the sequence, we observe an increase in the contribution of both inter- and intramolecular hydrogen bonds. The analysis of individual clusters (Figure 5B) shows that in most cases, we observe the inversely proportional tendency of inter/intrahydrogen bond formation. The clusters (OU1–OU3) with the highest input of intramolecular hydrogen bonds exhibit the lowest involvement of intermolecular hydrogen bonds. The data suggest that BPA may compete for the donors and acceptors of the hydrogen bonds of oligourethane. Interestingly, the exception is the OU4 sequence, for which the formation of a unique helical complex was observed. The analyzed helix structure of the SSSRS cluster displays an average of 1.29 intramolecular hydrogen bonds, just slightly higher than the overall average across the entire SSSRS complex ensemble −1.01 hydrogen bonds, indicating that structure regularity might not correlate with the number of hydrogen bonds in those systems.

**Figure 6:**
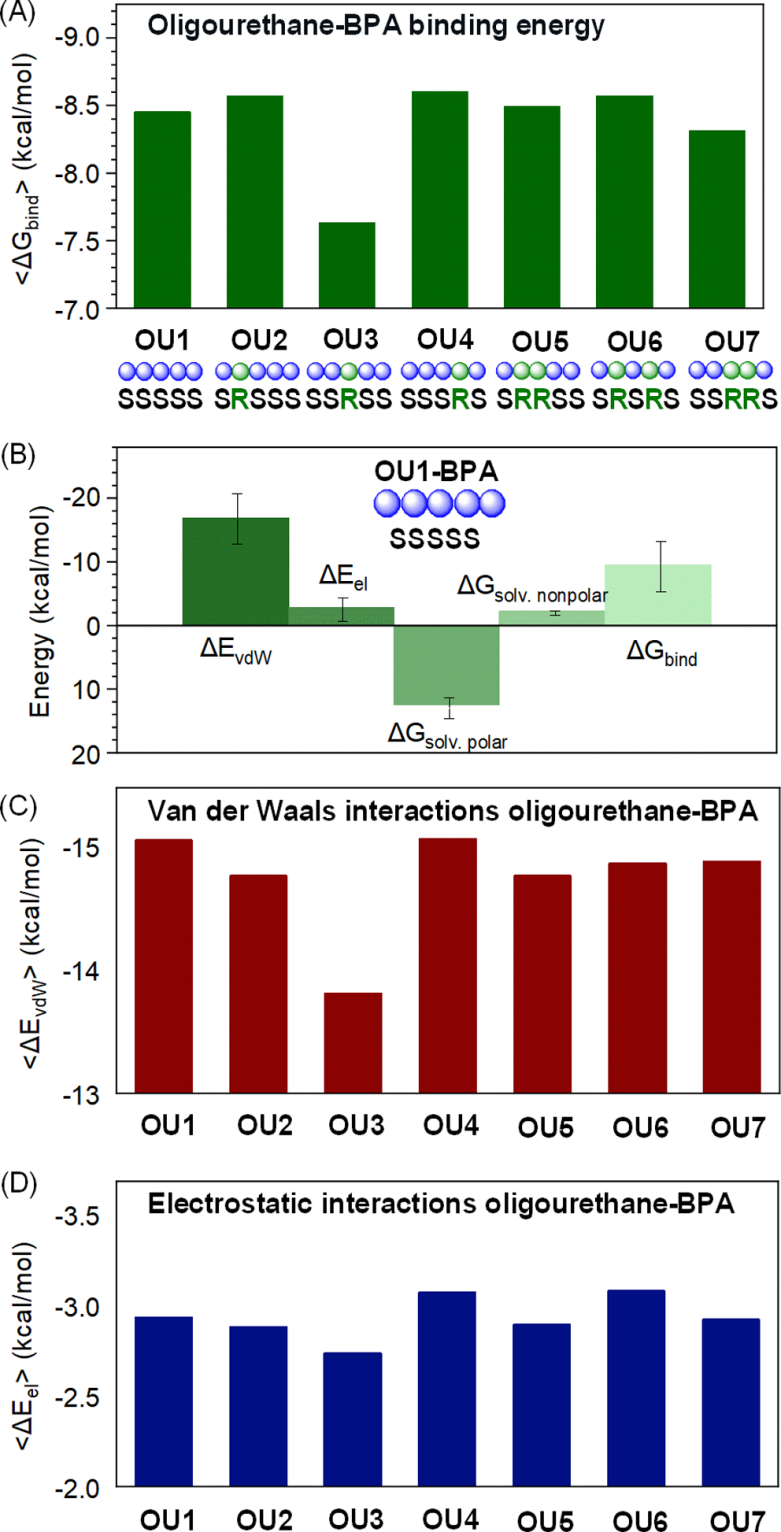
Analysis of receptor-like functionality of stereocontrolled oligourethanes by Gibbs binding energy calculations. The average Δ*G*_bind_ values show a substantial variance, indicating that each oligomer engages the ligand with a characteristic strength (A). When Δ*G*_bind_ for the OU1–BPA complex is decomposed into its energy components (B), a main contribution of dispersive forces Δ*E*_vdW_ to the binding process becomes evident. The bars on the chart indicate the statistical distribution of the energy values for the whole ensemble. (C) The Δ*E*_vdW_ values for each oligomer–BPA complex. (D) Contribution of electrostatic interactions Δ*E*_el_ to oligourethane–BPA binding.

The receptor-like functionality of the studied oligourethanes (OU1–OU7) was demonstrated through variations in Gibbs binding energy values (Δ*G*_bind_) calculated for oligourethane–BPA complexes. We found that Δ*G*_bind_ varies with the stereochemistry of the oligomer as shown by the molecular mechanics-generalized Born surface area (MM-GBSA) calculations (Figure 6).^68^ The details of the calculations are provided in the Supporting Information (Section 4.5). To visualize the general influence of mutations of stereocenters on BPA binding, the average Δ*G*_bind_ for the whole ensemble has been calculated (Figure 6A). For all investigated complexes, its values range from −7.70 to −8.70 kcal/mol between oligomers (Table S4), and each oligourethane–BPA system is characterized by a unique set of binding force magnitudes (Figure S34A). The data clearly show that the stereoconfiguration modulates the intermolecular binding function that relates to the diversity of attained structures. The stereochemical center manipulation has the most prominent effect if the middle monomer is mutated to the opposite configuration (OU3 SSRSS).

Through the decomposition analysis of Δ*G*_bind_ energies (depicted in Figure 6B), we gain insights into the nature of interactions between the oligomer and ligand. Two major components of Δ*G*_bind_ are van der Waals energy (Δ*E*_vdW_) and electrostatic energy (Δ*E*_el_) representing two molecular mechanics nonbonding terms describing noncovalent interactions. Other elements of the Δ*G*_bind_ value comprise interactions between the solute and continuous solvent divided into polar (Δ*G*_solv.polar_) and nonpolar (Δ*G*_solv.nonpolar_), which constitute free energy of solvation (Δ*G*_solv._) dominated by the polar component representing repellent force (Figure S34B,C). For all studied oligomers, the total effect of solvation energy gives a positive value, which depends on the complex shape. Thus, the data indicate that the solvent-accessible surface area and available polar interactions relate to the stereochemistry of oligourethane. The dominant contribution to Δ*G*_bind_ is the vdW interaction, indicating that dispersive forces are the major forces responsible for complex formation. The mutation of stereocenters leads to changes in Δ*E*_vdW_ above 1.23 kcal/mol (Figure 6C). In the case of electrostatic interactions that involve hydrogen bonds, the variations in the Δ*E*_el_ value with stereochemistry are minor, and differences are below 0.3 kcal/mol (Figure 6D). Interestingly, the highest Δ*E*_el_ values are observed in the case of complexes (OU4 and OU6) that can attain a helical conformation.

### 2.3 Experimental Analysis of Oligomer–BPA Interactions

Establishing a relevant protocol adjusted to the studied system is critical to getting reliable information about the interaction between molecules. Numerous experimental methods are applied to analyze molecular interactions, which are mostly validated for biological systems.^69,70^ We evaluated NMR,^71^ circular dichroism,^72,73^ and fluorescence spectroscopy^74−76^ techniques to study oligourethane–BPA interactions.

**Figure 7:**
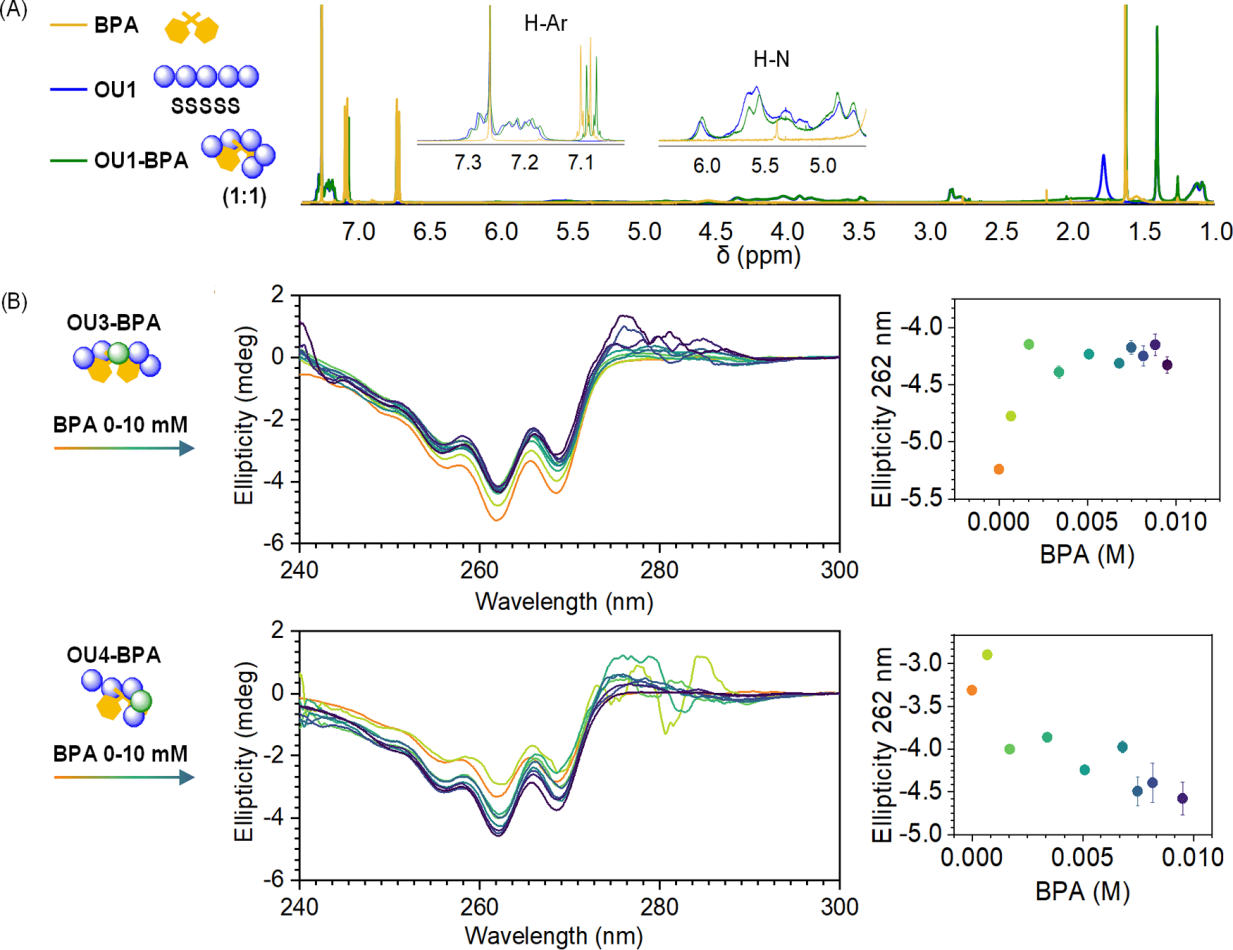
(A) The superimposed ^1^H NMR spectrum shows that the presence of BPA caused alteration of the signals. The greatest changes are visible in the ranges of amine and aromatic protons. (B) Complex formation is observed via CD titration measurements. As the concentration of BPA increased, an increase in the CD signal intensity was noted for OU3 SSRSS (top), signifying the emergence of the complex that impacts the spatial arrangement of the oligomer. On the contrary, a decrease in the CD signal was observed upon titration of BPA in the case of OU4–BPA SSSRS (bottom).

Formation of the complex causes changes in the chemical environments of protons belonging to the binding molecules, which can be followed by NMR. ^1^H NMR analyses of oligourethane–BPA complexes (oligomer:BPA, 1:1 molar ratio) revealed alterations in spectra compared to characteristics of individual compounds (Figures 7A and S36–S42). We observe changes in the shape of signals coming from N–H protons, meaning that BPA is disturbing the oligomer hydrogen bond net. The most noticeable deviations in the spectra appear in the aromatic region, where we observe a clear shift of signals attributed to aromatic protons of BPA. This characteristic shift of about 0.01 ppm is visible for all studied oligomers OU1–OU7, suggesting interactions between both molecules (Table S5). To confirm the formation of the complex, we performed a two-dimensional nuclear Overhauser effect spectroscopy (2D NOESY) experiment for a representative OU5–BPA system. The analysis revealed cross-peaks between the aromatic signal of BPA and oligomer phenyl moiety (Figure S35). Relatively small and irregular changes of ^1^H NMR spectra, without a clear tendency, observed for oligomers upon BPA titration reflect the structural dynamics of complexes and the diversity of ligand binding sites, as presented by cluster analyses.^77^ The chemical shifts of NMR signals are very structure-dependent; hence, studies of dynamic systems forming various complexes do not provide representative data of the whole ensemble, yet the obtained data confirm interactions between both molecules.

The chiral configuration of oligourethanes is an attribute that allows us to investigate interactions between oligomers and BPA using circular dichroism spectroscopy. Oligourethanes built from aromatic chiral phenylalaninol monomers (P_s_) show characteristic CD signals in the range of 240–280 nm (Figures S43–S49); therefore, it may act as an excellent probe to follow structural changes of complexes as BPA is a CD silent ligand. Consequently, CD results demonstrate various interactions occurring between the oligomer and BPA depending on stereochemical arrangements. Increasing concentration of the BPA ligand leads to a significant change in the CD signal intensity for all oligomers (Figures 7B and S43–S49). All spectra apart from OU4–BPA SSSRS show an increase in the CD signal amplitude. Remarkably, OU4 is a unique sequence where a helical conformation has been found by molecular dynamics calculations. A change in the signal intensity indicates the formation of the complexes, affecting the three-dimensional structure of the oligomers, which may be a consequence of adjustments in the skeletal conformation of oligomers to the ligand molecule. However, no typical titration trend matches the obtained CD intensity curves, apart from the SSRSS oligomer (Figure 7B). Similar to NMR, CD spectroscopy is a technique revealing structural details of formed pairs, which demonstrates that data are unsuitable for representing a structurally diverse ensemble.

**Figure 8:**
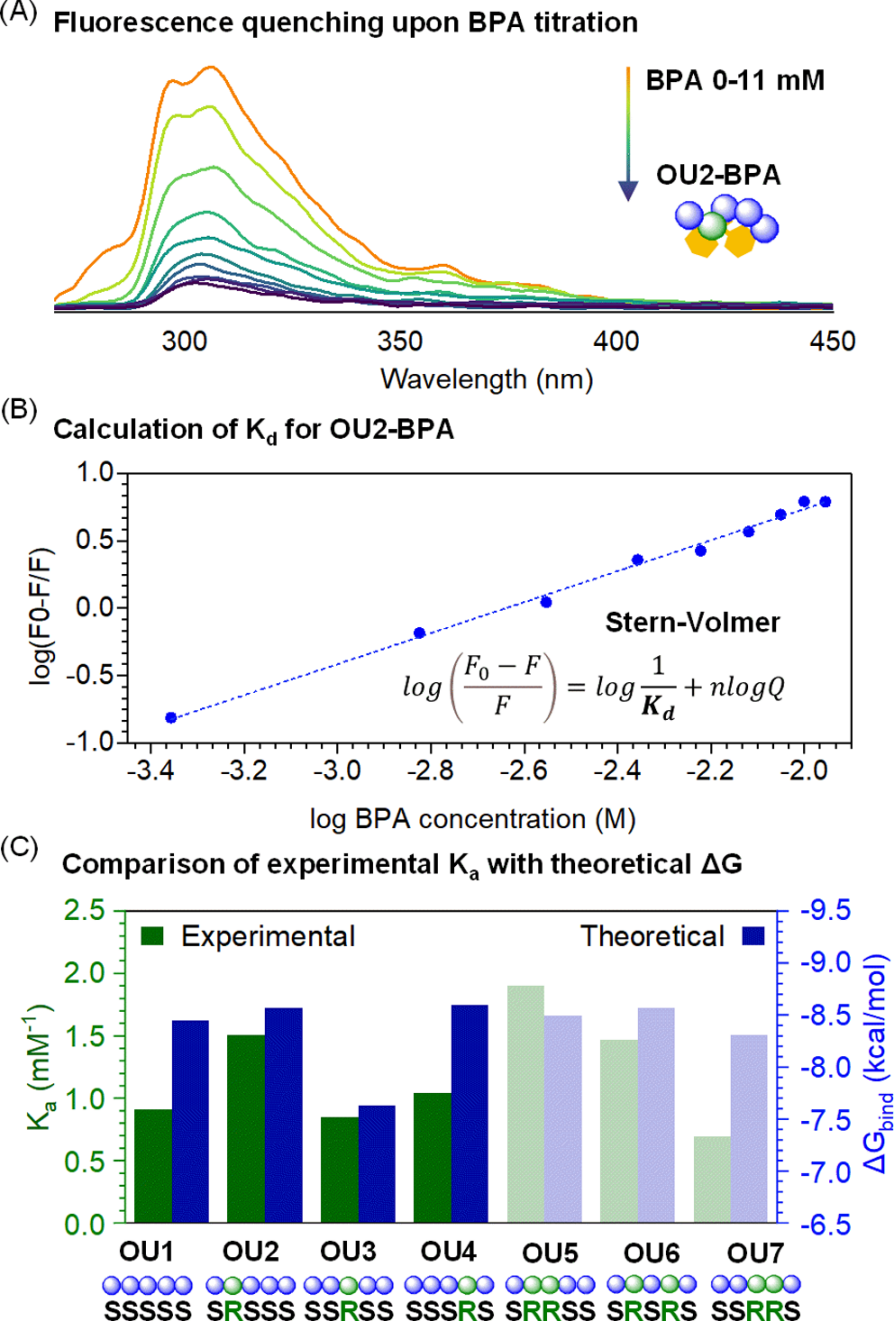
(A) Fluorescence spectra of OU2 (68 μM) recorded in chloroform (orange). BPA titration (0–11 mM) causes quenching of oligourethane fluorescence. (B) Fluorescence quenching was used to calculate dissociation constant (*K*_d_) values based on the Stern–Volmer equation, where *F* is the measured fluorescence, *F*_0_ is the fluorescence of oligourethane solution before BPA is added, and *Q* is the quencher (BPA) concentration. (C) Both experiment-derived *K*_a_ (*K*_a_ = 1/*K*_d_) and the theoretically calculated Δ*G*_bind_ demonstrate a compatible value variation depending on the sequence of stereocenters.

Investigated oligourethanes composed of P_s_ monomers with a benzylic substituent exhibit fluorescent properties, similar to phenylalanine-containing peptides; therefore, interactions with BPA can be followed by emission measurements.^78^ Interactions between molecules often lead to alterations in the intensity, shape, or position of the receptor fluorescence signal; hence, fluorescence is employed as a tool to investigate molecular binding through the measurement of its quenching,^74^ enhancement,^75^ anisotropy,^76^ or shift^79^ depending on the system. Oligourethanes are characterized by excitation and emission at the UV range with maxima at λ_Ex_ = 260 nm and λ_Em_ = 310 nm, while BPA exhibits weak activity within the specified wavelength range (Figure S50). For all studied oligomers, BPA titration leads to significant quenching of the fluorescence signal (Figures 8A and S51–S57). The intensity changes were fitted to the Stern–Volmer equation to determine the dissociation constant (*K*_d_) values for all investigated systems (Figure 8B).^80^ Those values were inversed to obtain association constants (*K*_a_), which were used as a representative parameter to describe the receptor-like functionality of oligourethane toward BPA and compared with theoretical Δ*G*_bind_ values derived from molecular dynamics (Figure 8C).

An alteration of the sequence of stereocenters influences the *K*_a_ values, leading to a subsequent decrease in the Gibbs free binding energy. Comparing the theoretical and experimental data across the entire library of oligourethane–BPA complexes reveals a consistent trend of changing parameters that describe the receptor-like functionality of oligomers. Computational studies show an approximate 70% success rate in trend predictions. Notably, some oligomers displayed a significant decrease in *K*_a_ values with respect to Δ*G*_bind_, indicating the reduced stability of formed oligourethane–BPA complexes. However, discrepancies may relate to the mechanism of a quenching phenomenon that is not considered in calculations but can influence the experimentally determined *K*_a_. Nevertheless, both methods indicate which oligomer is characterized by the most promising receptor-like features (OU5) and appoints the least efficient sequence (OU3). The OU5 shows the most potent interaction with BPA among all studied systems, as indicated by experimental and theoretical approaches. At the computational level, OU5 is in the top 4 oligomers with the highest binding energy values. Differences between them are minor, e.g., a bit diverse value of Δ*G*_bind_ than for OU5 is revealed by OU2 (0.14 kcal/mol), OU4 (0.20 kcal/mol), and OU6 (0.04 kcal/mol). We did not notice a connection between the ability of oligomers to form a secondary helical structure (OU4 and OU6) and its effectiveness in BPA binding. A high number of hydrogen bonds do not guarantee strong binding, as could be expected. OU3, which forms the most hydrogen bonds (Figure 5), is characterized as the weakest receptor (Figure 8C). This observation highlights the leading role of hydrophobic interactions. The presented data emphasize the importance of sequence programming through stereoconformation changes, which directly affects the spatial arrangement of the complex and the strength of binding with the ligand. The developed computational methodology enables the screening of abiotic oligomers and, in silico, identifies abiotic oligomers characterized by binding function toward the chosen ligands, revealing stereochemistry effects.

## 3 Conclusions

The structural properties of sequence-defined oligourethanes hold great potential to form nuanced shapes, which can be controlled by a rational design. Engineering non-natural macromolecule functionalities demands effective tools to study their conformation and molecular interactions. Structure and function studies of biopolymers, such as proteins, are already well-established; thanks to that, we have a broad range of tools to characterize biological macromolecules that facilitate research outcomes. However, we must refine and adjust existing protocols to characterize abiotic systems. Binding between abiotic sequence-defined oligomers and ligands can be induced by rational design based on the supramolecular chemistry background. Oligourethanes equipped with structural motifs, such as phenyl rings and hydrogen bond donors/acceptors, enable attractive interactions with a bisphenol A ligand. Regardless of the stereocenter(s) mutation pattern, all sequences interact with a BPA primarily via vdW rather than electrostatic interaction despite a strongly nonpolar environment, which would enhance the latter. The predominance of nondirectional vdW forces over directional interactions, such as hydrogen bonding, results in complex behavior with a low level of specificity. Studied systems display a range of possible complex conformations interacting with the ligand with different strengths yet showing similar binding characteristics. Such a nature of attraction and liability may render it a nonspecific receptor. Since the dispersive forces are not directional, they allow the binding of structurally similar compounds. On the other hand, the flexibility of the macromolecule may enhance that effect by adopting diverse conformations depending on the type of ligand. Matching the shape of the oligomer and the ligand is key to achieving reliable binding, allowing the highest number of interactions and supramolecular bonds to be formed. Amplification of the polymer molar mass is expected to cause an augmentation in the frequency and strength of interactions between the polyurethane and the ligand, as longer chains will have an increased number of sites to form hydrogen-bonding, π–π stacking, and van der Waals interactions. We speculate that the elongation of polymers to the level of protein size will improve the specificity of binding with the ligand. Moreover, to improve the design of oligourethanes, we should consider the additional polar groups to forge an impactful number and strength of directional electrostatic interactions between molecules. To advance the specificity of the convoluted binding mechanism of BPA, a broad sequence space of oligomers must be explored to find matching structural motifs.

The demonstrated MD simulation methodology is emerging as an invaluable tool for the in silico screening of various structures to optimize receptor-like functionalities of oligourethanes. The method reveals the impact of subtle structural changes, such as the effect of stereocenter sequence (e.g., 1 kcal Δ*G*_bind_ difference). As we demonstrated for NMR and CD spectroscopies, such flexible systems are challenging to characterize by structure-sensitive methodologies. We considered fluorescence measurements a complementary technique for verifying the simulation data. The observed fluorescence quenching is not too sensitive to the structural diversity of complexes and delivers data representative of the entire ensemble of conformations. Combining the developed MD methodology with the Stern–Volmer model is an efficient strategy for predicting and validating receptor-like functionalities of oligomer–ligand complexes that can be used for future structure optimization and the development of sensory materials using abiotic, sequence- and stereo-controlled polymers.

## Supporting Information Available

All experimental details, spectra (NMR, CD, fluorescence), chromatograms (LC–MS, GPC), and a detailed description of simulations (PDF)
