# Supplementary material for: Revealing the Effect of Stereocontrol on Intermolecular Interactions between Abiotic, Sequence-Defined Polyurethanes and a Ligand
Source: ACS Biomater Sci Eng. 2024 May 28;10(6):3727–38. doi: 10.1021/acsbiomaterials.4c00456 (PMC11167595; doi:10.1021/acsbiomaterials.4c00456)
Supplement: Supplementary file 1 — ab4c00456_si_001.pdf [file ab4c00456_si_001.pdf]

Supporting Information for publication

Revealing the Effect of Stereocontrol on Intermolecular Interactions between Abiotic, Sequence-defined Polyurethanes and a Ligand

Maksymilian Szatko[a,b,#], Weronika Forysiak[a,c,#], Sara Kozub[a], Tadeusz Andruniów[b,*], Roza Szweda[a,d,*]

[a]M. Szatko, W. Forysiak, S. Kozub, Dr. R. Szweda
Łukasiewicz Research Network – PORT Polish Center for Technology Development
Stabłowicka 147, 54-066 Wrocław, Poland

[b]Prof. T. Andruniów, M. Szatko
Department of Chemistry, Wroclaw University of Science and Technology
Wybrzeże Wyspiańskiego 27, 50-370 Wroclaw, Poland
*Email: tadeusz.andruniow@pwr.edu.pl

[c]W. Forysiak
Faculty of Chemistry, University of Wrocław
F. Joliot-Curie 14, 50-383 Wrocław, Poland

[d]Dr. R. Szweda
Center for Advanced Technologies, Adam Mickiewicz University
Uniwersytetu Poznańskiego 8, 61-614 Poznań, Poland
*Email: roza.szweda@amu.edu.pl

# These authors contributed equally to this work.

CONTENT

1. EXPERIMENTAL PROCEDURES **5**

1.1. MATERIALS AND CHEMICALS 5

1.2. SYNTHESIS OF N-(TERT-BUTOXYCARBONYL)-(S)-2-AMINO-3-PHENYL-1-PROPANOL (BOC-P_S) 5

1.3. SYNTHESIS OF SEQUENCE-DEFINED OLIGOURETHANES 5

2. ANALYTICAL METHODS **11**

2.1. NUCLEAR MAGNETIC RESONANCE (NMR) 11

2.2. ULTRA-HIGH PERFORMANCE LIQUID CHROMATOGRAPHY (HPLC) 11

2.3. SIZE-EXCLUSION CHROMATOGRAPHY (SEC) 11

2.4. LIQUID CHROMATOGRAPHY – MASS SPECTROSCOPY (LC-MS) 12

2.5. CIRCULAR DICHROISM (CD) 12

2.6. FLUORESCENCE 13

3. CHARACTERIZATION OF OLIGOMERS **13**

3.1. SEC CHROMATOGRAMS 13

OU1 Boc-$P_SM_SM_SM_SP_S$ *13*

OU2 Boc-$P_SM_RM_SM_SP_S$ *14*

OU3 Boc-$P_SM_SM_RM_SP_S$ *15*

OU4 Boc-$P_SM_SM_SM_RP_S$ *16*

OU5 Boc-$P_SM_RM_RM_SP_S$ *17*

OU6 Boc-$P_SM_RM_SM_RP_S$ *18*

OU7 Boc-$P_SM_SM_RM_RP_S$ *19*

3.2. LC-MS DATA 20

OU1 Boc-$P_SM_SM_SM_SP_S$ *20*

OU2 Boc-$P_SM_RM_SM_SP_S$ *21*

OU3 Boc-$P_SM_SM_RM_SP_S$ *22*

OU4 Boc-$P_SM_SM_SM_RP_S$ *23*

OU5 Boc-$P_SM_RM_RM_SP_S$ *24*

OU6 Boc-$P_SM_RM_SM_RP_S$ *25*

OU7 Boc-$P_SM_SM_RM_RP_S$ *26*

3.3. NMR SPECTRA 27

OU1 Boc-$P_SM_SM_SM_SP_S$ *27*

OU2 Boc-$P_SM_RM_SM_SP_S$ *28*

OU3 Boc-$P_SM_SM_RM_SP_S$ *29*

OU4 Boc-$P_SM_SM_SM_RP_S$ *30*

OU5 Boc-$P_SM_RM_RM_SP_S$ *31*

OU6 Boc-$P_SM_RM_SM_RP_S$ *32*
OU7 Boc-$P_SM_SM_RM_RP_S$ *33*
3.4. ^{1}H NMR VARIABLE TEMPERATURE 34
3.5. ^{1}H NMR VARIABLE CONCENTRATION 36

4. SIMULATIONS 38

4.1. STARTING GEOMETRY AND PARAMETRIZATION 38
4.2. MULTIPLE SIMULATED ANNEALING – MOLECULAR DYNAMICS (MSA-MD) 38
4.3. STRUCTURAL ANALYSIS - RAMACHANDRAN PLOT 40
4.4. FORCE FIELD COMPARISON 47
4.5. MOLECULAR MECHANICS GENERALIZED BORN SURFACE AREA (MMGBSA) 48

5. NMR ANALYSIS OF OLIGOMER-BPA COMPLEX 51

5.1. NUCLEAR OVERHAUSER EFFECT SPECTROSCOPY (NOESY) 51
5.2. ^{1}H NMR OF OLIGOMERS-BPA 52
OU1 Boc-$P_SM_SM_SM_SP_S$:BPA, 1:1 molar ratio *52*
OU2 Boc-$P_SM_RM_SM_SP_S$:BPA, 1:1 molar ratio *53*
OU3 Boc-$P_SM_SM_RM_SP_S$:BPA, 1:1 molar ratio *54*
OU4 Boc-$P_SM_SM_SM_RP_S$:BPA, 1:1 molar ratio *55*
OU5 Boc-$P_SM_RM_RM_SP_S$:BPA, 1:1 molar ratio *56*
OU6 Boc-$P_SM_RM_SM_RP_S$:BPA, 1:1 molar ratio *57*
OU7 Boc-$P_SM_SM_RM_RP_S$:BPA, 1:1 molar ratio *58*

6. LIGAND TITRATION EXPERIMENTS 60

6.1. CIRCULAR DICHROISM ANALYSES 60
OU1 Boc-$P_SM_SM_SM_SP_S$ *60*
OU2 Boc-$P_SM_RM_SM_SP_S$ *60*
OU3 Boc-$P_SM_SM_RM_SP_S$ *61*
OU4 Boc-$P_SM_SM_SM_RP_S$ *61*
OU5 Boc-$P_SM_RM_RM_SP_S$ *62*
OU6 Boc-$P_SM_RM_SM_RP_S$ *62*
OU7 Boc-$P_SM_SM_RM_RP_S$ *63*
6.2. FLUORESCENCE ANALYSES 64
OU1 Boc-$P_SM_SM_SM_SP_S$ *65*
OU2 Boc-$P_SM_RM_SM_SP_S$ *66*
OU3 Boc-$P_SM_SM_RM_SP_S$ *67*
OU4 Boc-$P_SM_SM_SM_RP_S$ *68*
OU5 Boc-$P_SM_RM_RM_SP_S$ *69*

OU6 Boc-$P_SM_RM_SM_RP_S$ *70*
OU7 Boc-$P_SM_SM_RM_RP_S$ *71*
6.3. CHARACTERIZATION OF OLIGOMER-BPA BINDING 72
7. REFERENCES 73

1. EXPERIMENTAL PROCEDURES

1.1. MATERIALS AND CHEMICALS

(S)-(+)-2-Aminopropan-1-ol, (M_S, 98%, ApolloScientific, ee: 97%, optical rotation: [α]20/D +18°), (R)-(-)-2-aminopropan-1-ol, (M_R, 98%, Fluorochem, ee: 97%, optical rotation: [α]19/D −18°), (S)-2-amino-3-phenyl-1-propanol (P_S, 97%, Angene, ee: 99%, optical rotation: [α]22/D −22.8°), acetonitrile for HPLC (ACN, ≥ 99.9%, gradient grade ChemSolve), acetonitrile for synthesis (ACN, 99.9%, extra dry, Sigma Aldrich), anisole (98%, Alfa Aesar), di-tert-butyl dicarbonate (Boc_2O, 99%, Sigma-Aldrich), $CDCl_3$ (99.96%, Sigma-Aldrich), diethyl ether (99.5%, p.a, Fisher Scientific), ethyl acetate (EtOAc, 99,5%, p.a., POCH-BASIC), N,N'-disuccinimidyl carbonate (DSC, >98%, TCI), methanol-d4 (99,8%, Sigma-Aldrich), pyridine (PYR, 99.5%, extra dry, Acros Organics), sodium sulfate (>99%, Sigma-Aldrich).

1.2. SYNTHESIS OF N-(TERT-BUTOXYCARBONYL)-(S)-2-AMINO-3-PHENYL-1-PROPANOL (BOC-P_S)

Synthesis of initiator Boc-P followed the literature procedure.[1] P monomer (2 g, 13.2 mmol, 1 mol equiv.) was dissolved in MiliQ water (10 mL). The solution was stirred at 30°C for 30 minutes. Next, liquid di-tert-butyl dicarbonate (Boc_2O, 3.343 mL, 14.5 mmol, 1.1 mol equiv.) was added dropwise to the reaction mixture. After adding Boc_2O, transparent liquid droplets appeared on the water surface. The reaction was conducted for 4 hours upon vigorous mixing. The product was isolated by extraction with diethyl ether (40 mL) and brine (20 mL). Boc-P_S was obtained with a yield of 89.5% and its structure was confirmed by ^{1}H NMR.

1.3. SYNTHESIS OF SEQUENCE-DEFINED OLIGOURETHANES

Synthesis of oligourethanes was performed as described previously.[2] The representative experimental protocol for Boc-$P_SM_SM_SM_SP_S$ synthesis is described below.

The initiator (N-(tert-butoxycarbonyl)-(S)-2-amino-3-phenyl-1-propanol (Boc-P_S, 0.7960 g, 1 mol equiv.) and N,N'-disuccinimidyl carbonate (DSC, 0.9730 g, 1.2 mol equiv.) were introduced into a round-bottom flask. The vessel was closed with a rubber septum to perform the reaction under an inert environment. Therefore, the flask was degassed and filled with nitrogen; the procedure was repeated 3 times. Next, 20 mL of anhydrous acetonitrile, anisole (250 µL), and anhydrous pyridine (950 µL, 6 mol equiv.) were added to the reaction mixture, starting the activation phase (i). The

mixture was stirred at room temperature for 4h, analyzing the reaction progress by RP-HPLC. After completing the activation step, the (S)-2-Amino-1-propanol (M_S, 295 μL, 1.2 mol equiv.) monomer was introduced into the reaction mixture, starting coupling phase (ii). The reaction was left overnight. HPLC confirmed complete reaction conversion. After the coupling phase, solvents were evaporated at 40°C under reduced pressure using a rotary evaporator. The product purification was done by extraction with ethyl acetate (20 mL) and distilled water (10 mL). The organic phase containing the product was collected and dried with anhydrous Na_2SO_4. Next, the mixture was filtered, and the solvent was removed at 40°C under reduced pressure, ending the purification phase (iii). Those three phases (i), (ii), and (iii) were repeated to make Boc-$P_SM_SM_SM_SP_S$. The final purification step in every sequence required a modification of the extraction procedure, i.e., diethyl ether was added to the mixture (8mL) to improve the phase separation.

All synthesized sequences are compiled in Figure S1. The quantities of reagents used in each synthesis are given in Table S1. Table S2 summarizes stepwise yields.

Before BPA-oligomer interaction studies, all sequences were repurified by crystallization at a 100 mg scale. Oligomers were recrystallized from 1/1 v/v of water/ACN mixture.

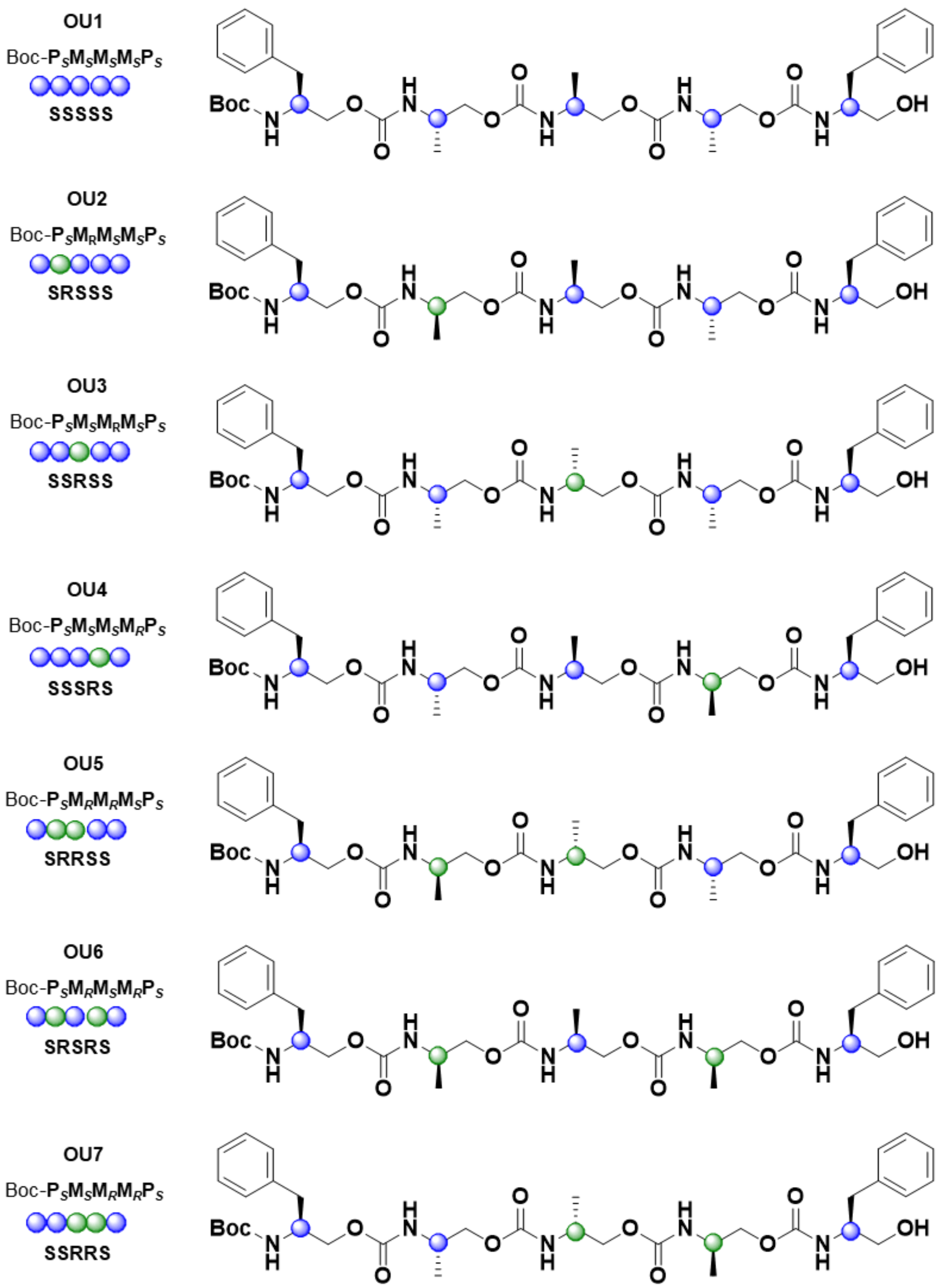

Figure S1. Structures of investigated oligourethanes OU1-OU7.

Table S1. Amount of reagents used in the synthesis of oligourethane sequences

			**Boc-$P_SM_SM_S$**		
	**Synthesis step**		**Amount of reagents**		**Added reagent excess**
**I**	activation	DSC	0.973 g	1.2 eq	
**I**	coupling	M_S	295 µL	1.2 eq	
**II**	activation	DSC	0.973 g	1.2 eq	
**II**	coupling	M_S	285 µL	1.2 eq	
			**Boc-$P_SM_SM_R$**		
	**Synthesis step**		**Amount of reagents**		**Added reagent excess**
**I**	activation	DSC	0.973 g	1.2 eq	
**I**	coupling	M_S	287 µL	1.2 eq	
**II**	activation	DSC	0.892 g	1.2 eq	
**II**	coupling	M_R	271 µL	1.2 eq	
			**Boc-$P_SM_RM_S$**		
	**Synthesis step**		**Amount of reagents**		**Added reagent excess**
**I**	activation	DSC	0.976 g	1.2 eq	
**I**	coupling	M_R	295 µL	1.2 eq	
**II**	activation	DSC	0.923 g	1.2 eq	
**II**	coupling	M_S	280 µL	1.2 eq	
			**Boc-$P_SM_SM_SM_SP_S$**		
	**Synthesis step**		**Amount of reagents**		**Added reagent excess**
**III**	activation	DSC	0.421 g	1.2 eq	
**III**	coupling	M_S	128 µL	1.2 eq	
**IV**	activation	DSC	0.427 g	1.2 eq	
**IV**	coupling	P_S	0.252 g	1.2 eq	
			**Boc-$P_SM_SM_SM_RP_S$**		
	**Synthesis step**		**Amount of reagents**		**Added reagent excess**
**III**	activation	DSC	0.426 g	1.2 eq	0.0463 g (0.1 eq.)
**III**	coupling	M_S	130 µL	1.2 eq	
**IV**	activation	DSC	0.38 g	1.2 eq	
**IV**	coupling	P_S	0.207 g	1.2 eq	
			**Boc-$P_SM_SM_RM_SP_S$**		
	**Synthesis step**		**Amount of reagents**		**Added reagent excess**
**III**	activation	DSC	0.429 g	1.2 eq	
**III**	coupling	M_S	124 µL	1.2 eq	
**IV**	activation	DSC	0.3017 g	1.2 eq	0.0243 g (0.8 eq.)

**IV**	coupling	P_S	0.2407 g	1.2 eq	
			**Boc-$P_SM_SM_SM_RP_S$**		
	**Synthesis step**		**Amount of reagents**		**Added reagent excess**
**III**	activation	DSC	0.440 g	1.2 eq	
**III**	coupling	M_R	130 µL	1.2 eq	
**IV**	activation	DSC	0.408 g	1.2 eq	
**IV**	coupling	P_S	0.240 g	1.2 eq	
			**Boc-$P_SM_RM_SM_RP_S$**		
	**Synthesis step**		**Amount of reagents**		**Added reagent excess**
**III**	activation	DSC	0.383 g	1.2 eq	0.1012 g (0.28 eq.)
**III**	coupling	M_R	117 µL	1.2 eq	
**IV**	activation	DSC	0.334 g	1.2 eq	
**IV**	coupling	P_S	0.1825 g	1.2 eq	
			**Boc-$P_SM_SM_RM_RP_S$**		
	**Synthesis step**		**Amount of reagents**		**Added reagent excess**
**III**	activation	DSC	0.455 g	1.2 eq	
**III**	coupling	M_R	137 µL	1.2 eq	
**IV**	activation	DSC	0.3391 g	1.2 eq	
**IV**	coupling	P_S	0.203 g	1.2 eq	
			**Boc-$P_SM_RM_RM_SP_S$**		
	**Synthesis step**		**Amount of reagents**		**Added reagent excess**
**I**	activation	DSC	0.557 g	1.2 eq	
**I**	coupling	M_R	148 µL	1.2 eq	
**II**	activation	DSC	0.580 g	1.2 eq	
**II**	coupling	M_R	155 µL	1.2 eq	
**III**	activation	DSC	0.5343 g	1.2 eq	
**III**	coupling	M_S	141 µL	1.2 eq	
**IV**	activation	DSC	0.48 g	1.2 eq	0.0822 g (0.18 eq.)
**IV**	coupling	P_S	0.261 g	1.2 eq	

Table S2. Stepwise yields obtained during the synthesis of oligourethanes

**Sequence**	**Initial mass**[a]	**Dimer**		**Trimer**		**Initial mass**[b]	**Tetramer**		**Pentamer**	
	mass [g]	mass [g]	yield [%]	mass [g]	yield [%]	mass [g]	mass [g]	yield [%]	mass [g]	yield [%]
**OU1** Boc-$P_{S}M_{S}M_{S}M_{S}P_{S}$	0.80	1.11	99.61	1.14	79.58	0.56	0.70	100	0.79	72.99
**OU2** Boc-$P_{S}M_{R}M_{S}M_{S}P_{S}$	0.79	1.05	94.84	1.19	83.25	0.62	0.63	82.33	0.79	67.95
**OU3** Boc-$P_{S}M_{S}M_{R}M_{S}P_{S}$	0.79	1.02	91.61	1.20	83.69	0.56	0.54	78.85	0.52	50.33
**OU4** Boc-$P_{S}M_{S}M_{S}M_{R}P_{S}$	0.80	1.11	99.61	1.14	79.58	0.58	0.67	94.22	0.52	46.27
**OU5** Boc-$P_{S}M_{R}M_{R}M_{S}P_{S}$	0.44	0.61	98.65	0.72	90.41	0.72[c]	0.79	89.84	0.97	77.85
**OU6** Boc-$P_{S}M_{R}M_{S}M_{R}P_{S}$	0.79	1.05	94.84	1.19	83.25	0.56	0.55	80.54	0.64	61.30
**OU7** Boc-$P_{S}M_{S}M_{R}M_{R}P_{S}$	0.79	1.02	91.61	1.20	83.69	0.608	0.61	83.28	0.62	56.36

a - mass of Boc-P used to initialize the oligomer synthesis. b – a mass of trimer used to continue the reaction. c – Boc-$P_{S}M_{R}M_{R}M_{S}P_{S}$ oligomer synthesis was performed continuously.

2. ANALYTICAL METHODS

2.1. NUCLEAR MAGNETIC RESONANCE (NMR)

NMR spectra were recorded in deuterated chloroform (d 99.8 atom % D, Sigma Aldrich) using Avance III HD 500 MHz NMR Spectrometer (BRUKER) equipped with probes: BBI and BBO. The recorded spectra were calibrated using a $CHCl_3$ signal as a reference and analyzed in MNova software.

2.2. ULTRA-HIGH PERFORMANCE LIQUID CHROMATOGRAPHY (HPLC)

The reverse phase ultra-high-performance liquid chromatography (RP-UHPLC) was used to monitor oligourethane syntheses. Analyses were performed using Thermo Scientific Dionex UltiMate 3000 with vacuum degasser SRD-3200; two-component quaternary pump, autosampler UltiMate™ WPS-3000SL/TSL, column thermostat TCC-3000SD, and diode array detector DAD-3000. The system was equipped with a reverse-phase column (150x4.6mm 5u Hypersil GOLD) with a guard. The chromatograms were recorded in the λ=190-400 nm range. Experimental conditions: phase A: 10% ACN (HPLC grade, ≥99.9%, Honeywell) in water (MiliQ), phase B: 100% ACN (HPLC grade, ≥99.9%, Honeywell); gradient elution: -1-0 min 10% B, 0-15 min 10-100% B, 15-18 min 100% B, 18-20 min 100-10% B; flow rate: 0.5 $mL \cdot min^{-1}$; T=25°C. The chromatograms were analyzed at 220 nm using Chromeleon software. The HPLC was used to control the conversion on each step of synthesis and for the characterization of final products.

2.3. SIZE-EXCLUSION CHROMATOGRAPHY (SEC)

Molar mass distributions and dispersities (OU1–OU7) were analyzed by SEC. The SEC setup consists of an Agilent system with a tertiary pump, column oven, UV (260-700 nm), and RI detectors. The analyses were performed using mixed PLGel E 3 µm, 300 x 7.5 mm column equipped with a guard. THF (HPLC grade, BHT stabilized, Chemsolute) was used as the mobile phase for analysis. The experimental details: temperature 30°C, 26 min elution time, 0.5 mL/min flow rate, sample concentration 2 mg/mL, injection 10 µL. The data were evaluated using Chemstation software.

2.4. LIQUID CHROMATOGRAPHY – MASS SPECTROSCOPY (LC-MS)

The LC-MS measurements were performed using a high-resolution Q-ToF spectrometer maXis impact (Bruker Daltonics, Germany) equipped with an electrospray ionization (ESI) source and connected to Dionex UltiMate 3000 RSLC (Thermo Scientific, USA) ultra-high-performance liquid chromatography system. The chromatographic separations were carried out on Syncronis C18 100 x 2.1 mm × 1.7 μm column (Thermo Scientific) in a gradient mode with a column oven set to 40°C. For LC-MS analyses, compound stock solutions 2 mg/mL were dissolved to a final concentration of 50 μg/mL using 1/1, v/v, water/ACN with 0.1% of formic acid. 2 μL. The applied gradient: 0–1 min 5% B, 1– 21 min from 5%–80% B, 21–21.5 min 80-95% B, 21.5–26.5 min 95% B, 26.5–30 min 5% B. In addition to MS detection, UV-DAD chromatograms at 220 nm and 260 nm wavelengths were recorded.

The spectrometer operated in automatic MS/MS (DDA) mode with a maximum number of precursors set to 3, the intensity cutoff threshold of 50000 units, and active precursor exclusion after 5 MS/MS spectra. The ESI source parameters were set as follows: end-plate offset 0.5 kV, capillary voltage 4 kV, nebulizing gas 2 bar, drying gas 8.0 L/min, and dry gas temperature 220°C. Measurements in MS mode were performed with an ion energy of 5.0 eV, collision energy of 7.5 eV, collision RF of 500 Vpp, a transfer time of 80 μs, pre-pulse storage of 8 μs, and low mass cutoff of 50 m/z. An internal calibration segment was added at the beginning of each chromatographic run. Spectra were calibrated using sodium formate clusters in HPC mode with SD < 1 ppm. The calibrant mixture of 10 mM NaOH in 1/1, v/v, water/isopropanol with the addition of 0.2% formic acid was provided by Sigma-Aldrich. The data were analyzed with Data Analysis 4.1 software. Molecular formulas corresponding to parent ions were generated using the SmartFormula algorithm with a maximum admissible error of 5 ppm.

2.5. CIRCULAR DICHROISM (CD)

CD spectra of oligourethanes OU1-OU7 and oligourethane-BPA complexes were measured on a Jasco J-815 apparatus. The analysis parameters were as follows: scanning speed 200 nm/min, band width 3.00 nm, D.I.T. 1 second, 5 spectra accumulations, cell length 1 mm, temperature 25°C. The standard error was calculated as the standard deviation of the mean for three measurements of each sample.

2.6. FLUORESCENCE

The fluorescence spectra were measured on SCINCO Fluorescence Spectrometer FluoroMate FS-2. The experimental processes were controlled by FluoroMaster Plus software. Samples for fluorescence measurements were prepared in $CHCl_3$ (HPLC grade) in a quartz cuvette with a 10x10 mm light path. Spectra were recorded at 260 nm excitation wavelength and 270-700 nm emission wavelength.

3. CHARACTERIZATION OF OLIGOMERS

3.1. SEC CHROMATOGRAMS

OU1 Boc-$P_SM_SM_SM_SP_S$

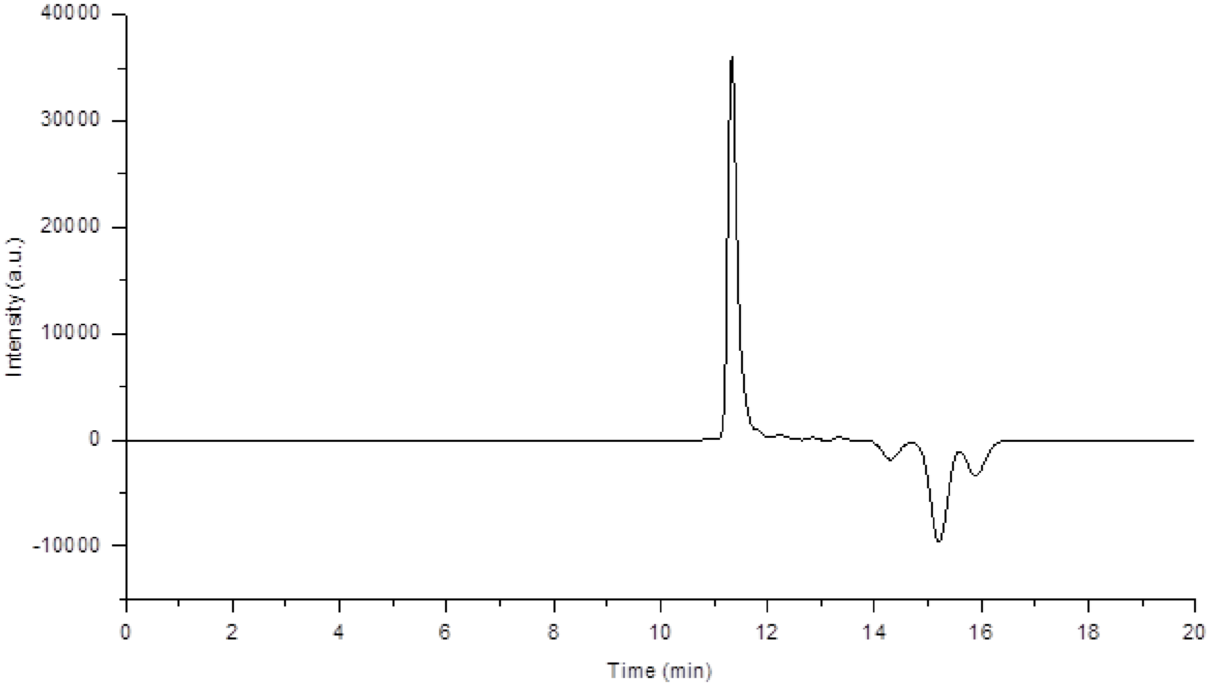

Figure S2. SEC analysis of OU1: R_t = 11.33 min, Đ=1.0.

OU2 Boc-$P_SM_RM_SM_SP_S$

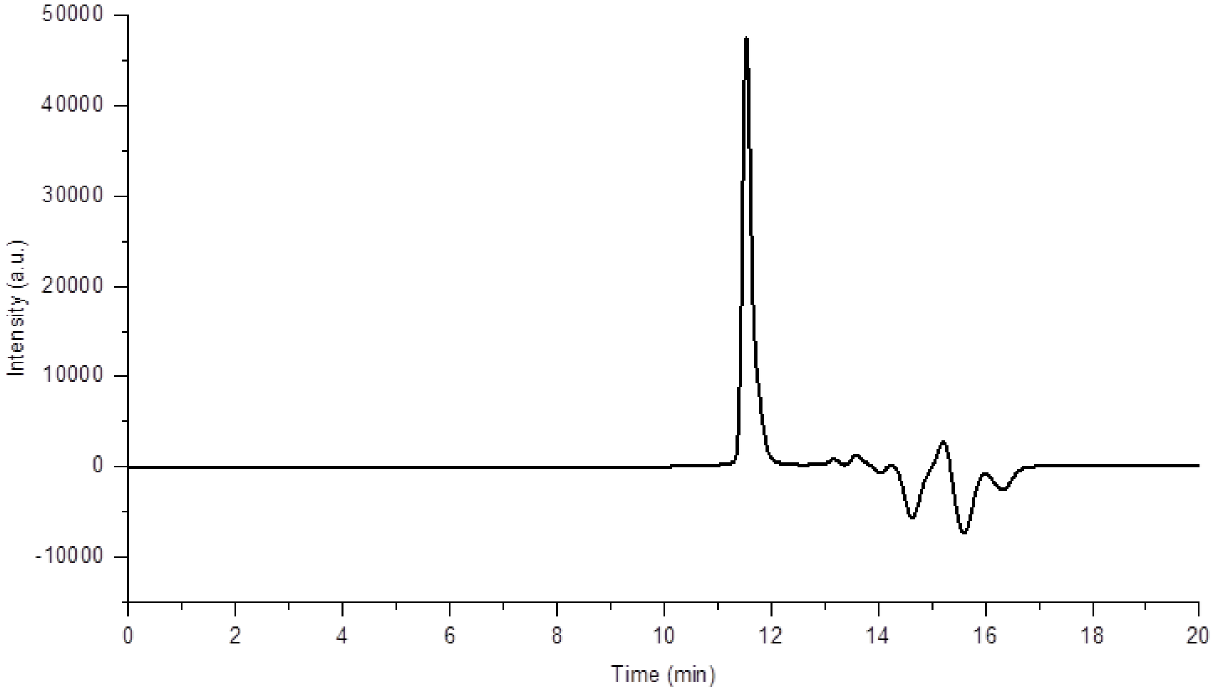

Figure S3. SEC analysis of OU2: Rt = 11.26 min, Đ=1.0.

OU3 Boc-$P_SM_SM_RM_SP_S$

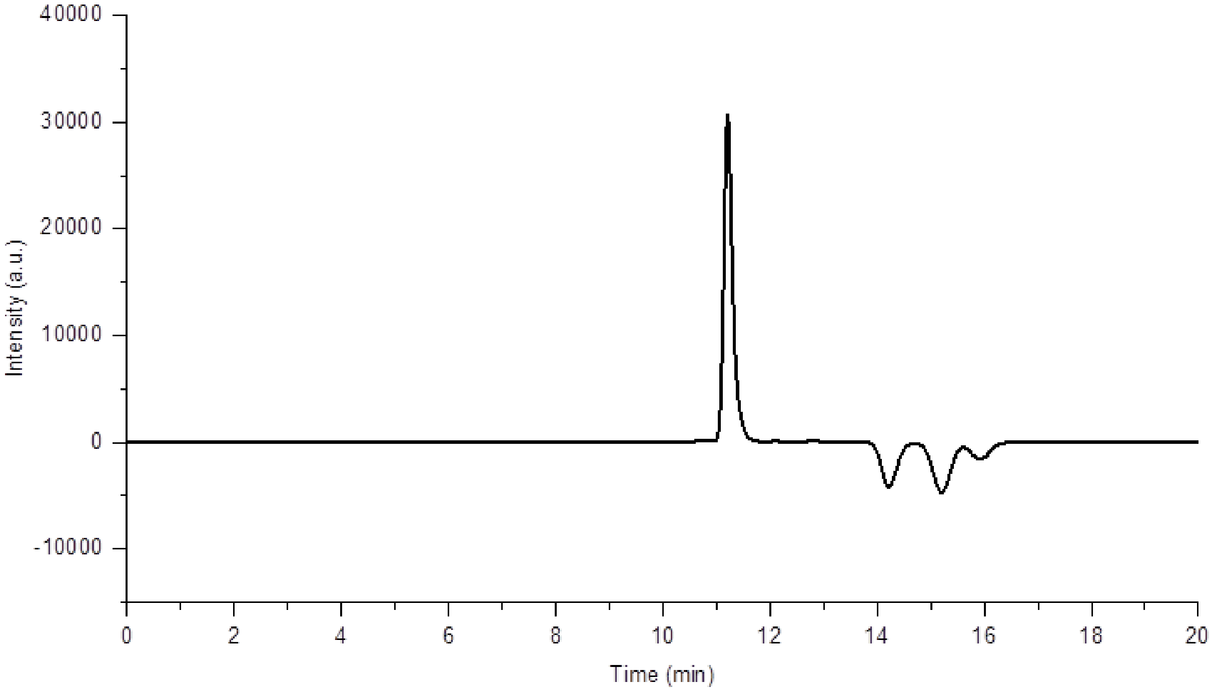

Figure S4. SEC analysis of OU3: Rt = 11.32 min, Đ=1.0.

OU4 Boc-$P_SM_SM_SM_RP_S$

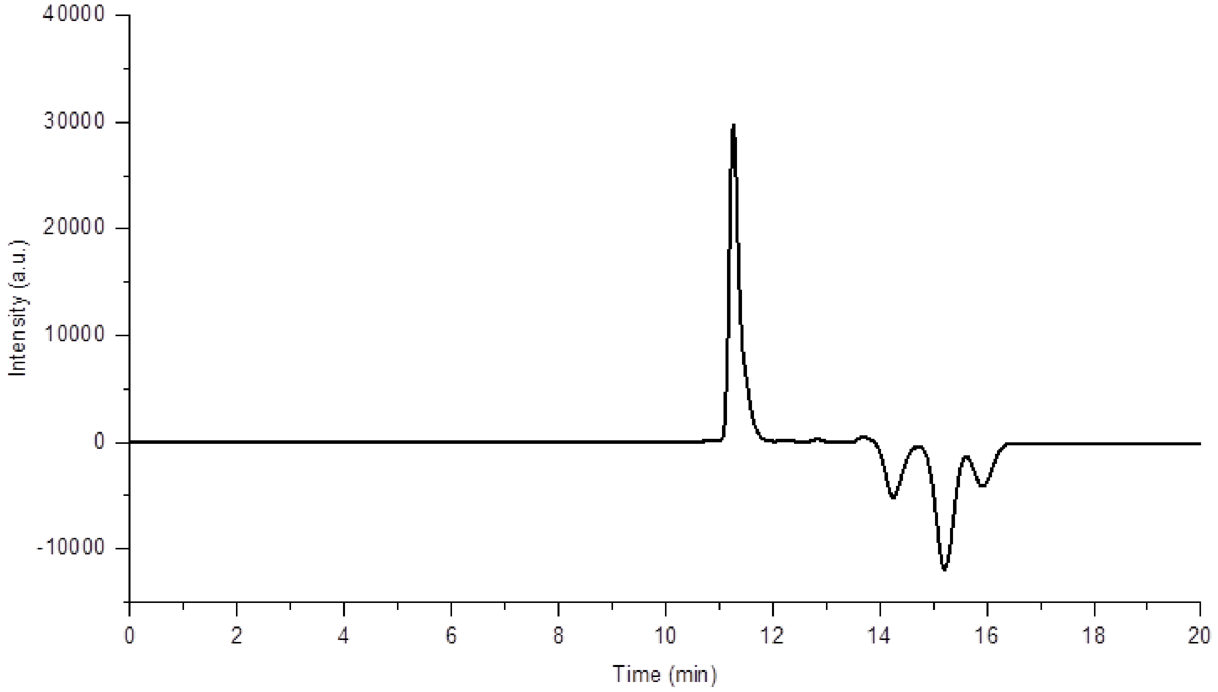

Figure S5. SEC analysis of OU4: R_t = 11.18 min, Đ=1.0.

OU5 Boc-$P_SM_RM_RM_SP_S$

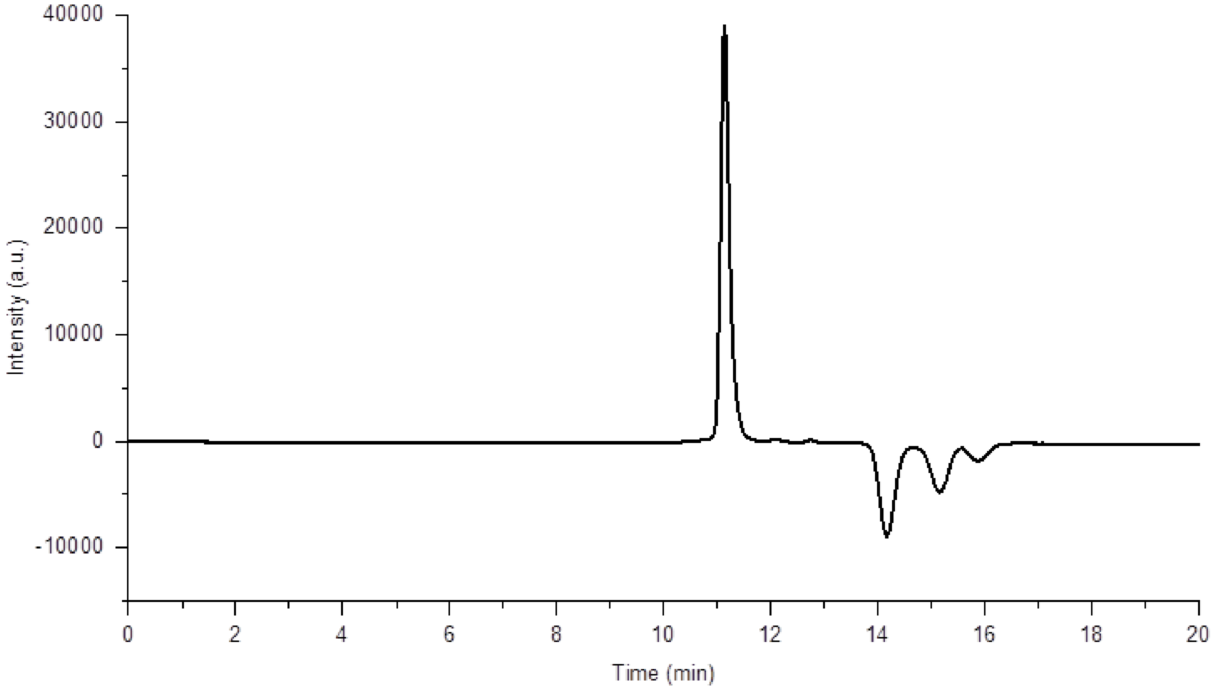

Figure S6. SEC analysis of OU5: R_t = 11.12 min, Đ=1.0.

OU6 Boc-$P_SM_RM_SM_RP_S$

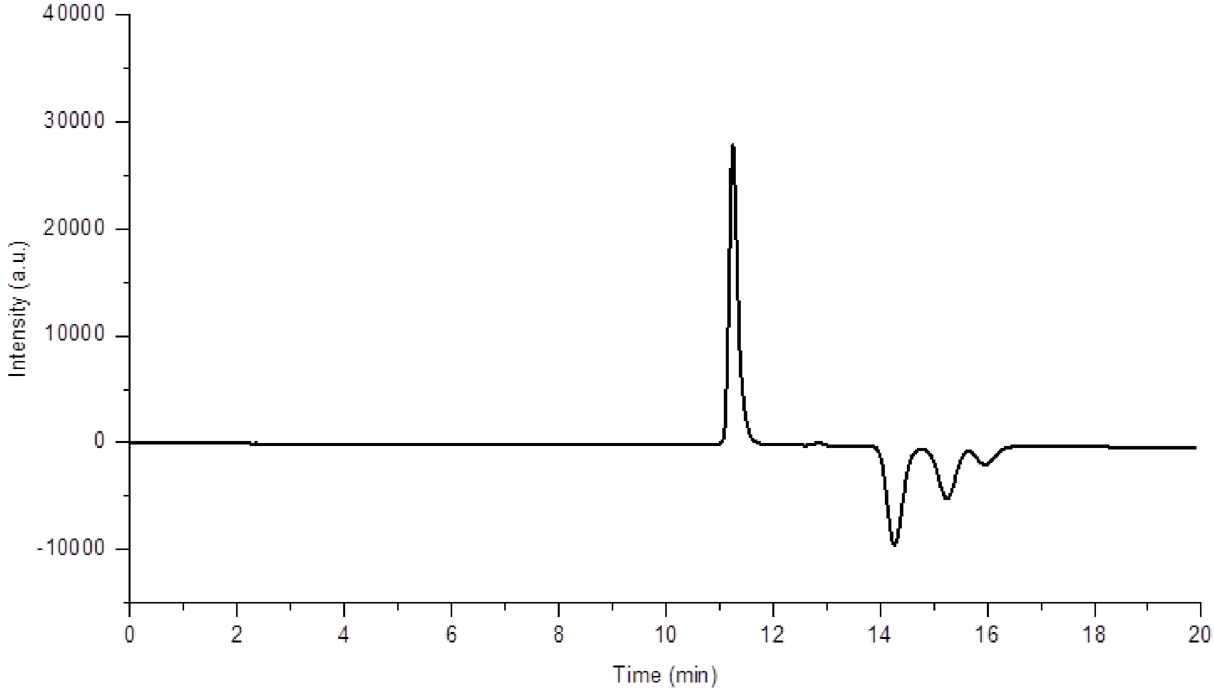

Figure S7. SEC analysis of OU6: R_t = 11.25 min, Đ=1.0.

OU7 Boc-$P_S M_S M_R M_R P_S$

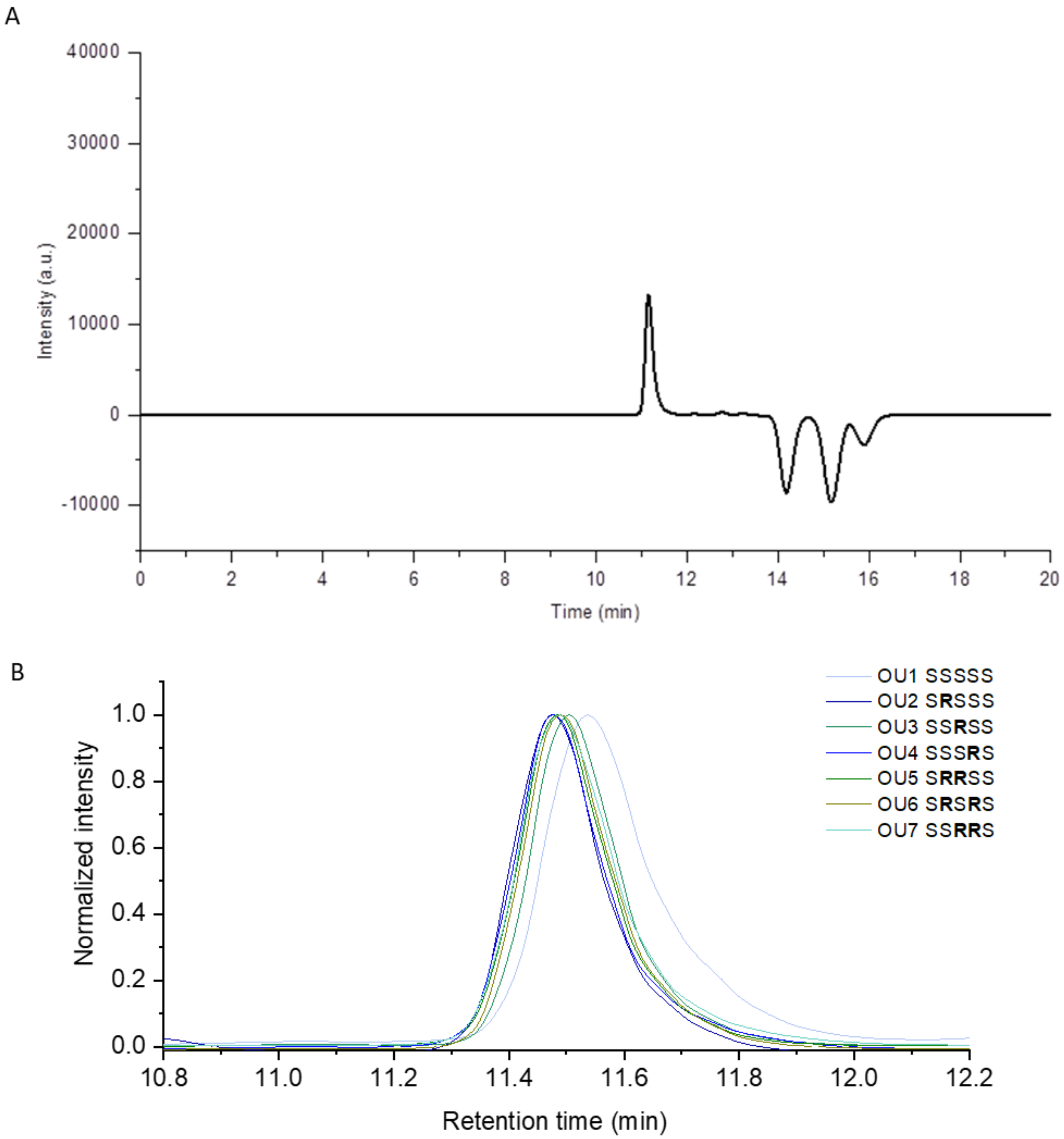

Figure S8. A) SEC analysis of OU7: R_t = 11.25 min, Đ=1.0. B) Comparison of retention time for OU1-OU7, chromatograms were calibrated according to THF signal.

3.2. LC-MS DATA

OU1 Boc-$P_SM_SM_SM_SP_S$

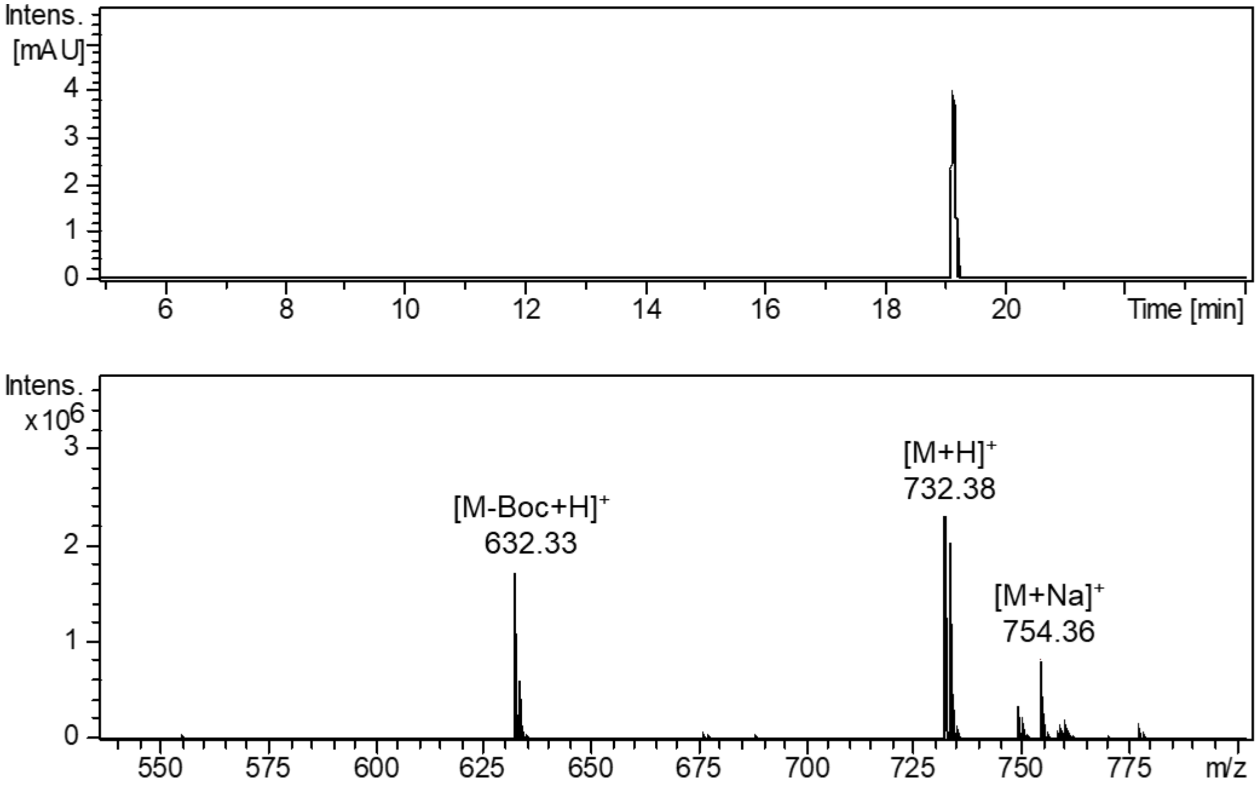

Figure S9. LC-MS characterization of OU1. UV chromatogram (top), and mass spectrum of the main peak R_t = 19.2 min (bottom).

OU2 Boc-$P_SM_RM_SM_SP_S$

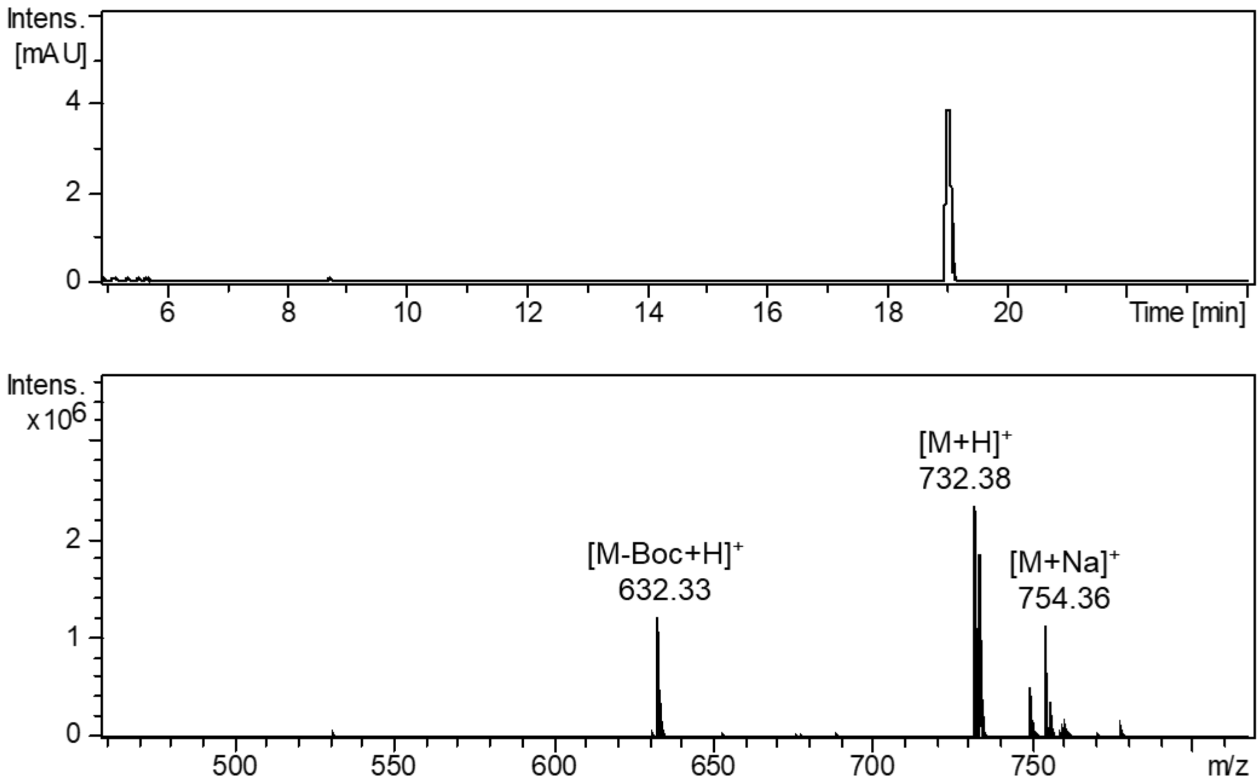

Figure S10. LC-MS characterization of OU2. UV chromatogram (top), and mass spectrum of the main peak R_t = 19.2 min (bottom).

OU3 Boc-$P_SM_SM_RM_SP_S$

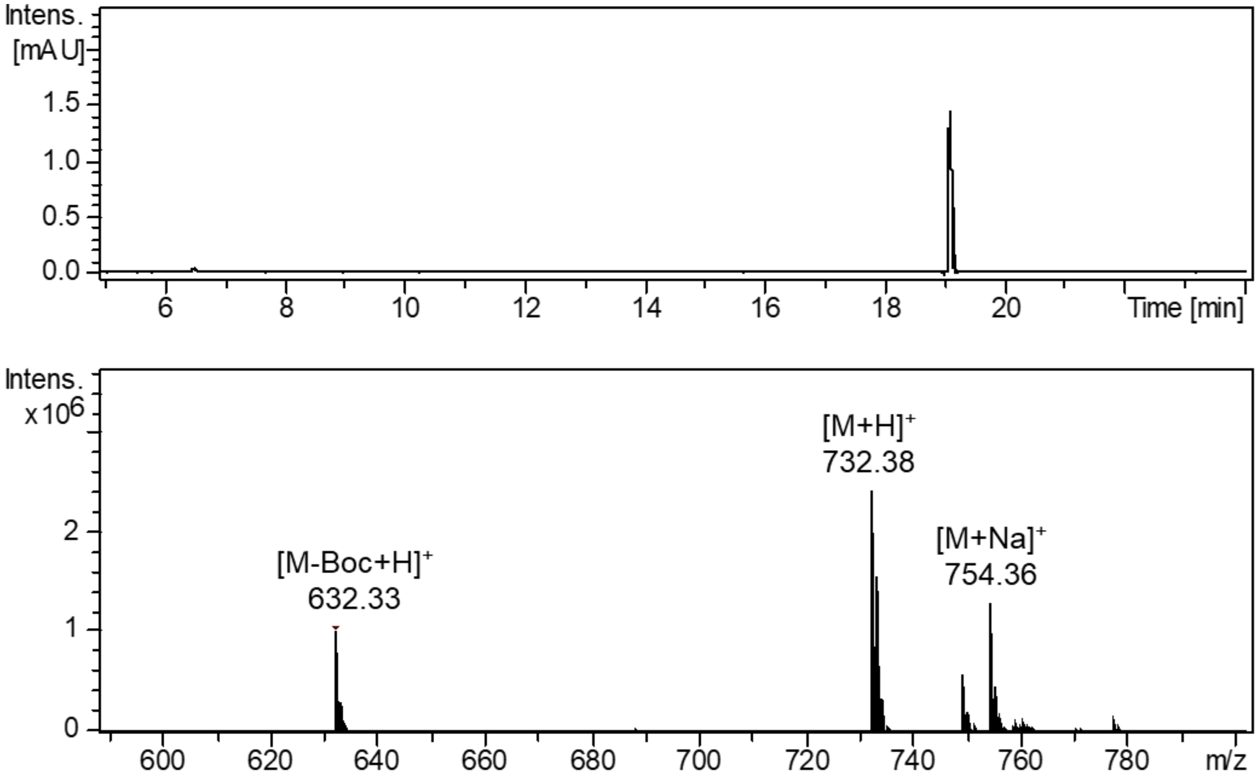

Figure S11. LC-MS characterization of OU3. UV chromatogram (top), and mass spectrum of the main peak R_t = 19.2 min (bottom).

OU4 Boc-$P_SM_SM_SM_RP_S$

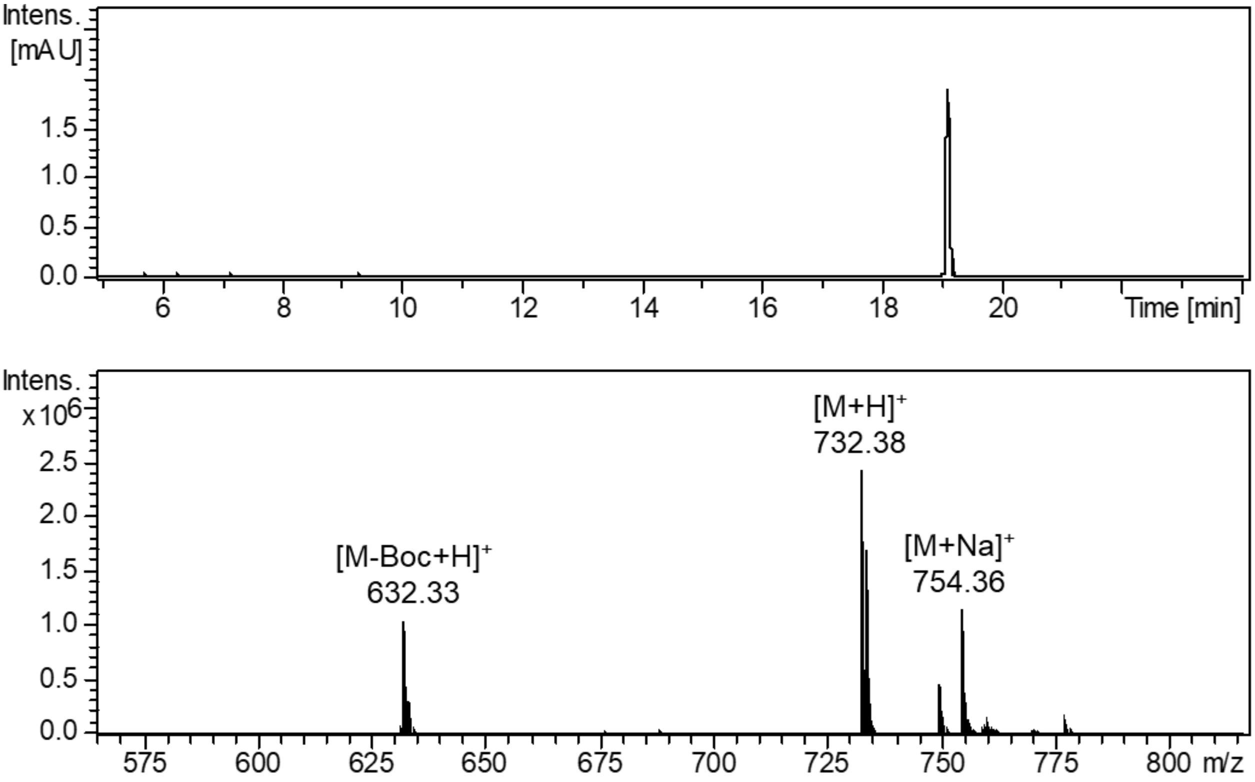

Figure S12. LC-MS characterization of OU4. UV chromatogram (top), and mass spectrum of the main peak R_t = 19.2 min (bottom).

OU5 Boc-$P_SM_RM_RM_SP_S$

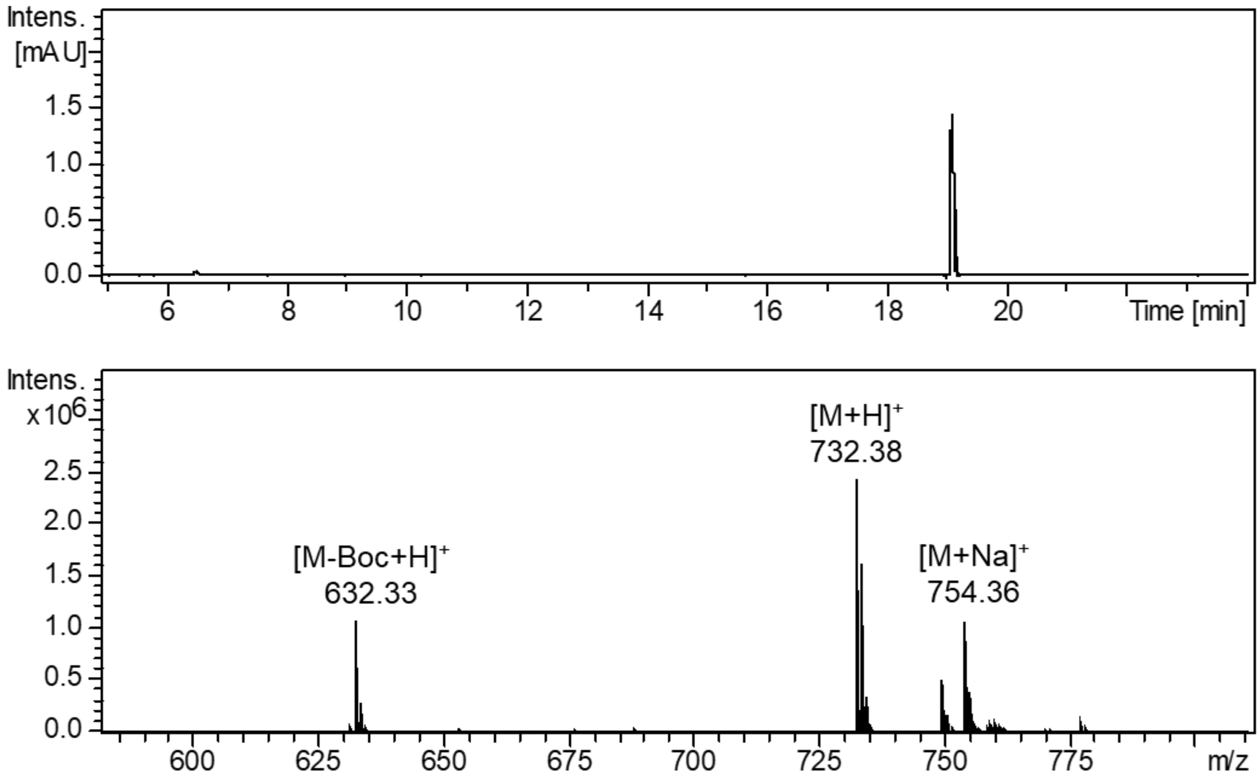

Figure S13. LC-MS characterization of OU5. UV chromatogram (top), and mass spectrum of the main peak R_t = 19.2 min (bottom).

OU6 Boc-$P_SM_RM_SM_RP_S$

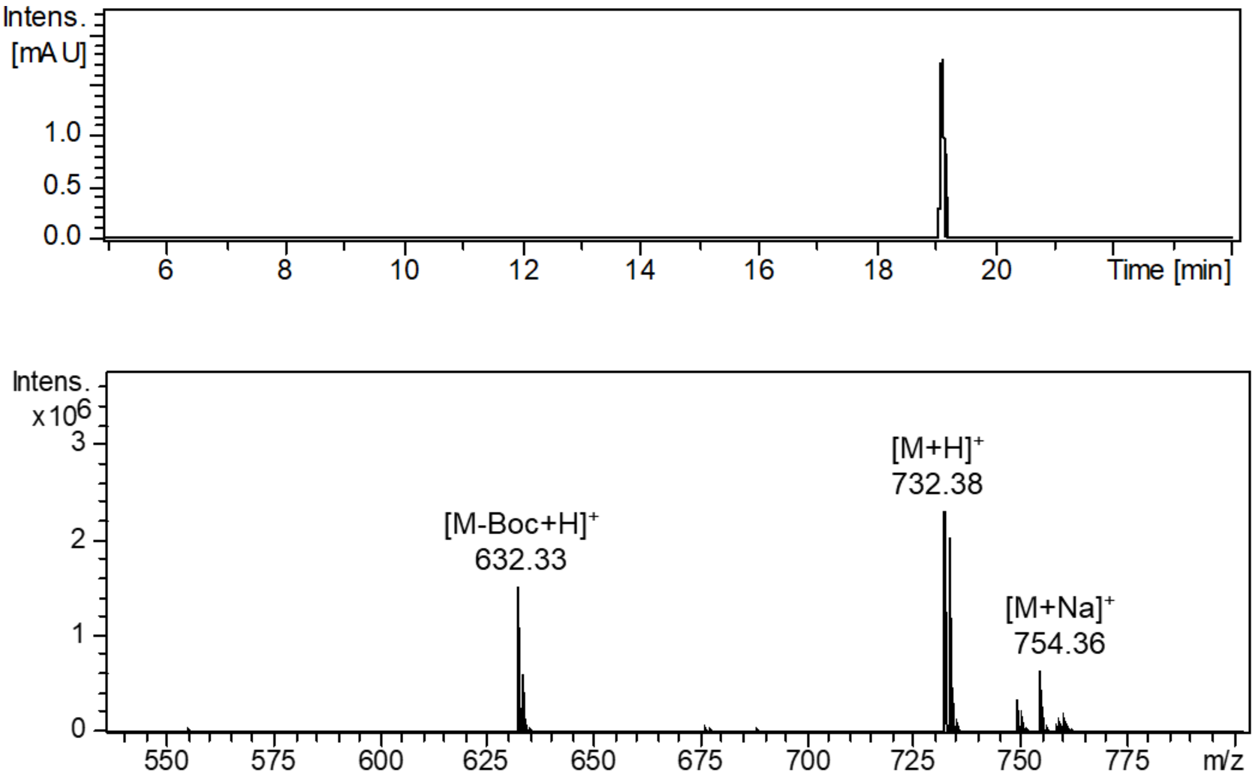

Figure S14. LC-MS characterization of OU6. UV chromatogram (top), and mass spectrum of the main peak R_t = 19.2 min (bottom).

OU7 Boc-$P_SM_SM_RM_RP_S$

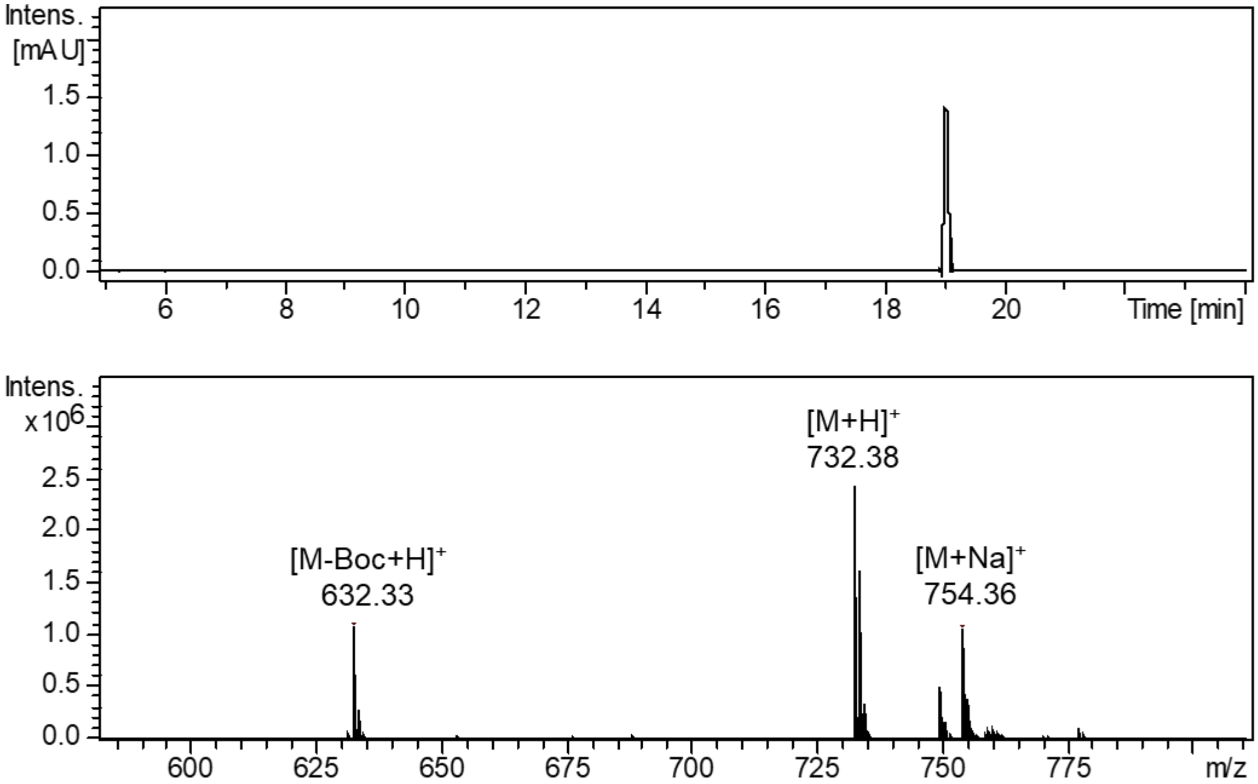

Figure S15. LC-MS characterization of OU7. UV chromatogram (top), and mass spectrum of the main peak R_t = 19.2 min (bottom).

3.3. NMR SPECTRA

OU1 Boc-PSMSMSMSPS

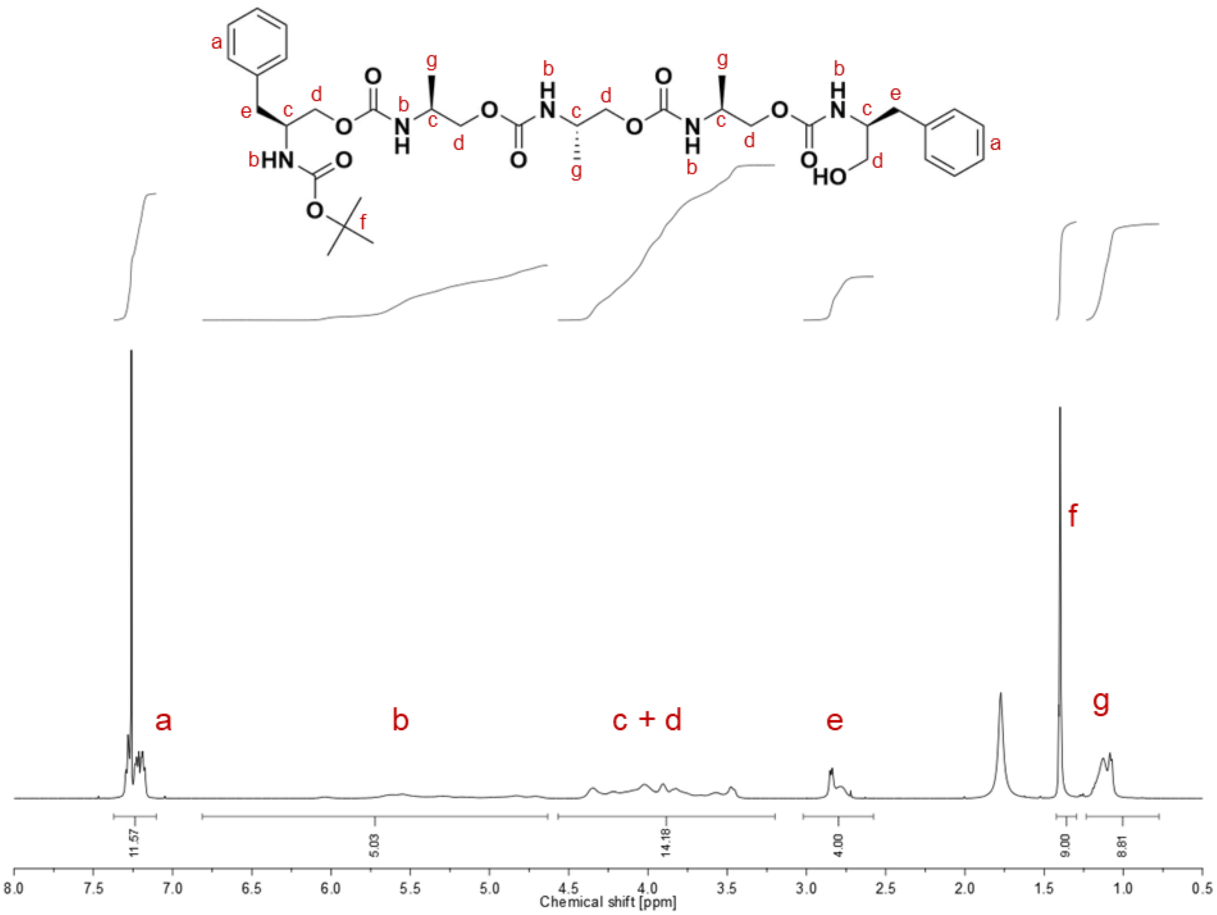

Figure S16. ^{1}H NMR spectrum of OU1 in $CDCl_3$, RT.

OU2 Boc-$P_S M_R M_S M_S P_S$

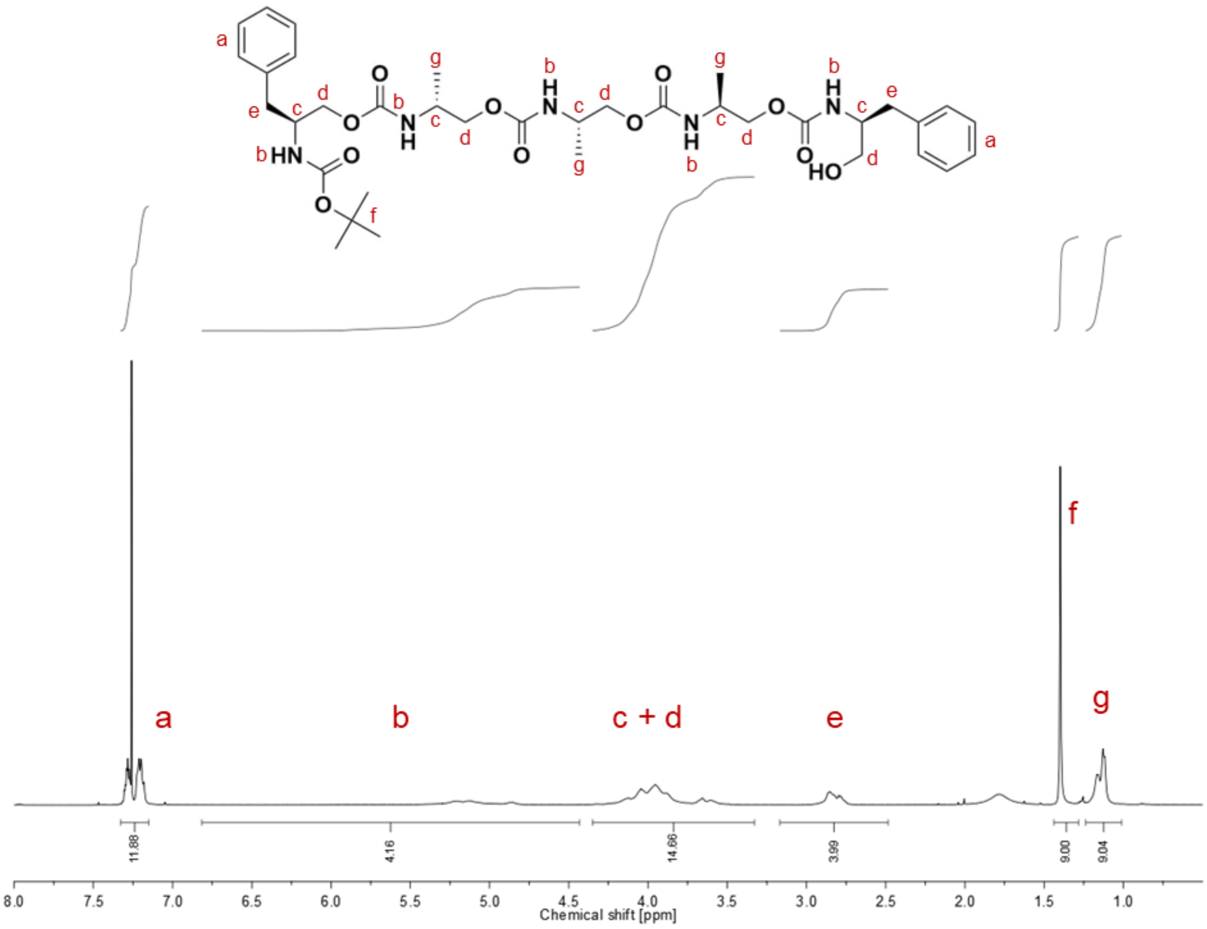

Figure S17. ^{1}H NMR spectrum of OU2 in $CDCl_3$, RT.

OU3 Boc-$P_SM_SM_RM_SP_S$

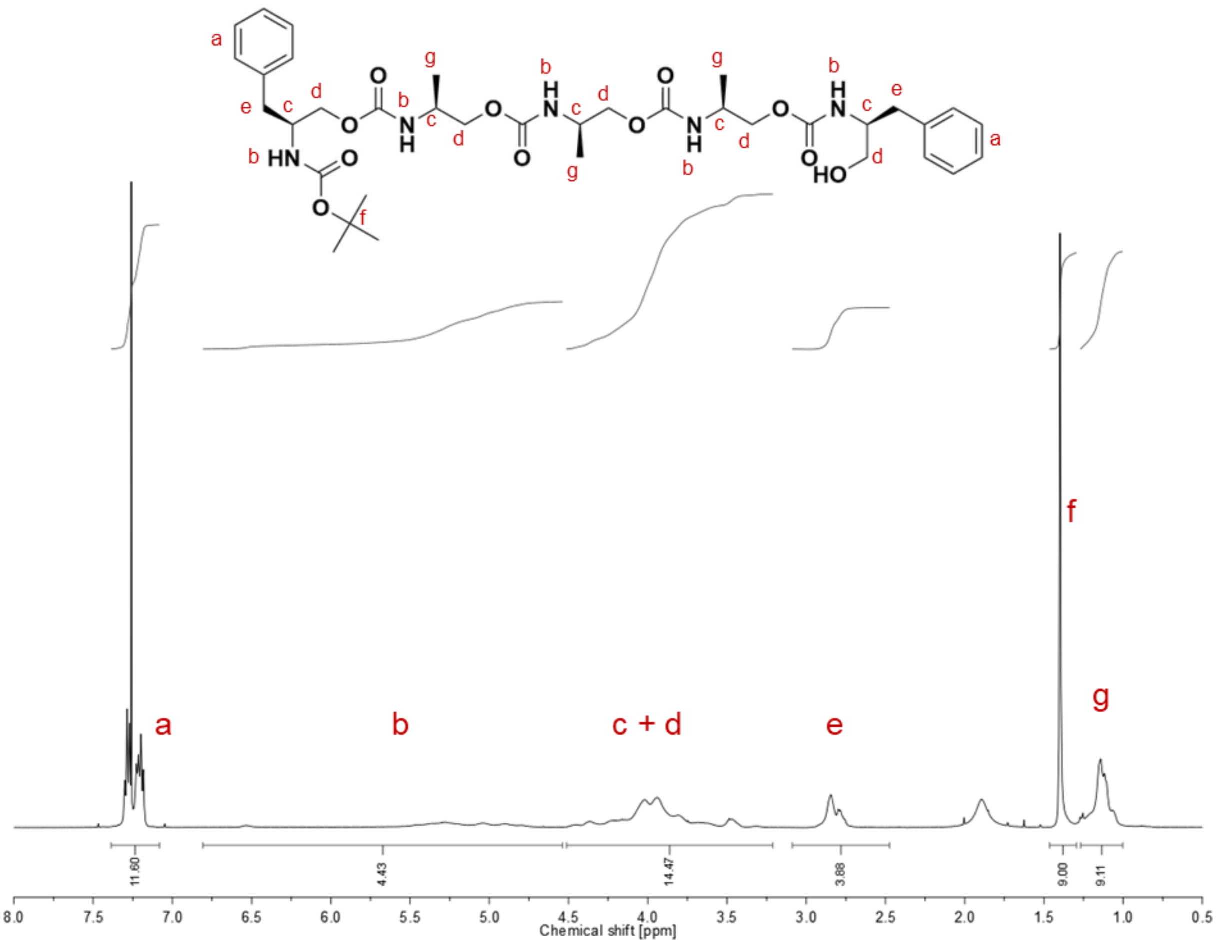

Figure S18. ^{1}H NMR spectrum of OU3 in $CDCl_3$, RT.

OU4 Boc-$P_SM_SM_SM_RP_S$

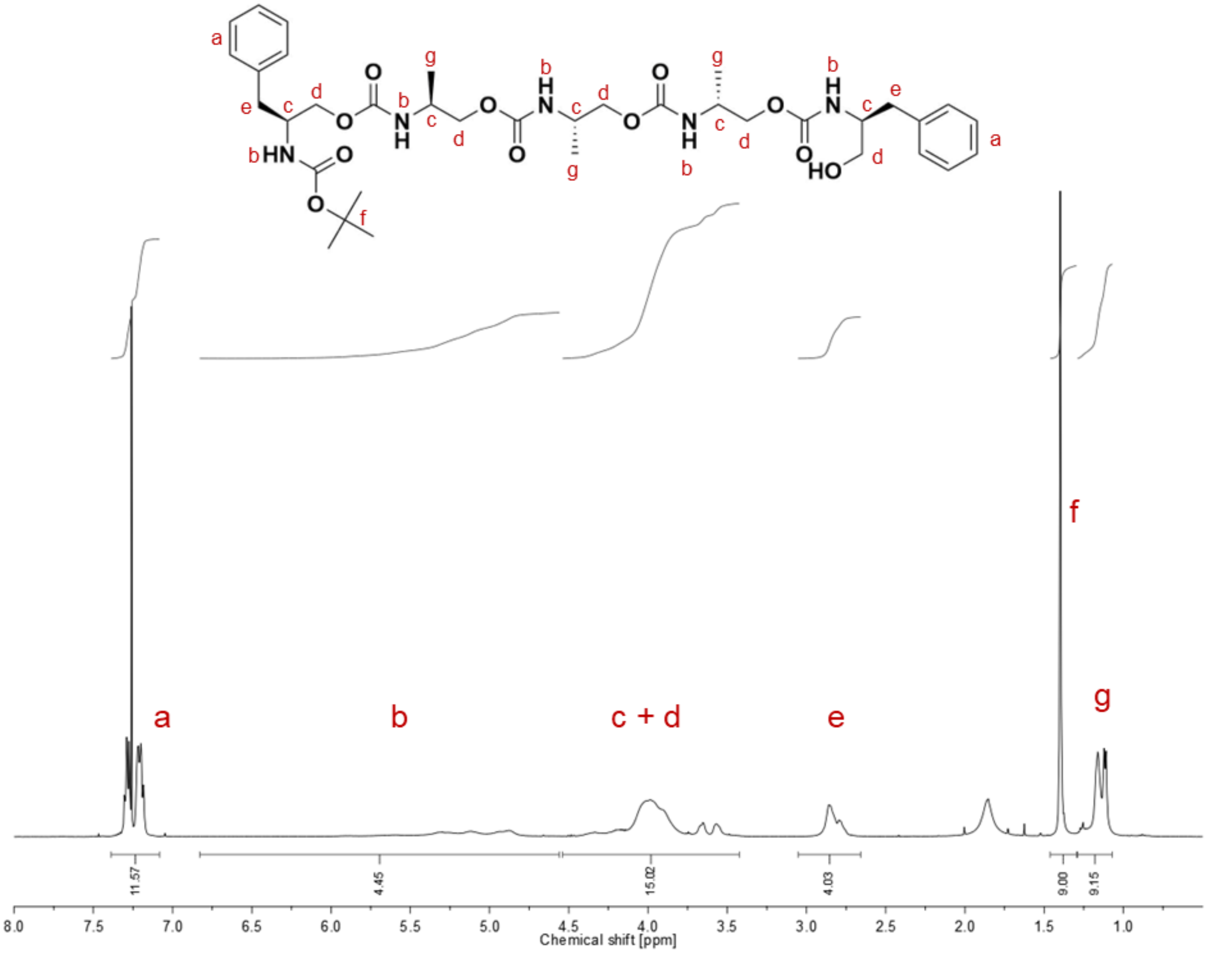

Figure S19. ^{1}H NMR spectrum of OU4 in $CDCl_3$, RT.

OU5 Boc-$P_SM_RM_RM_SP_S$

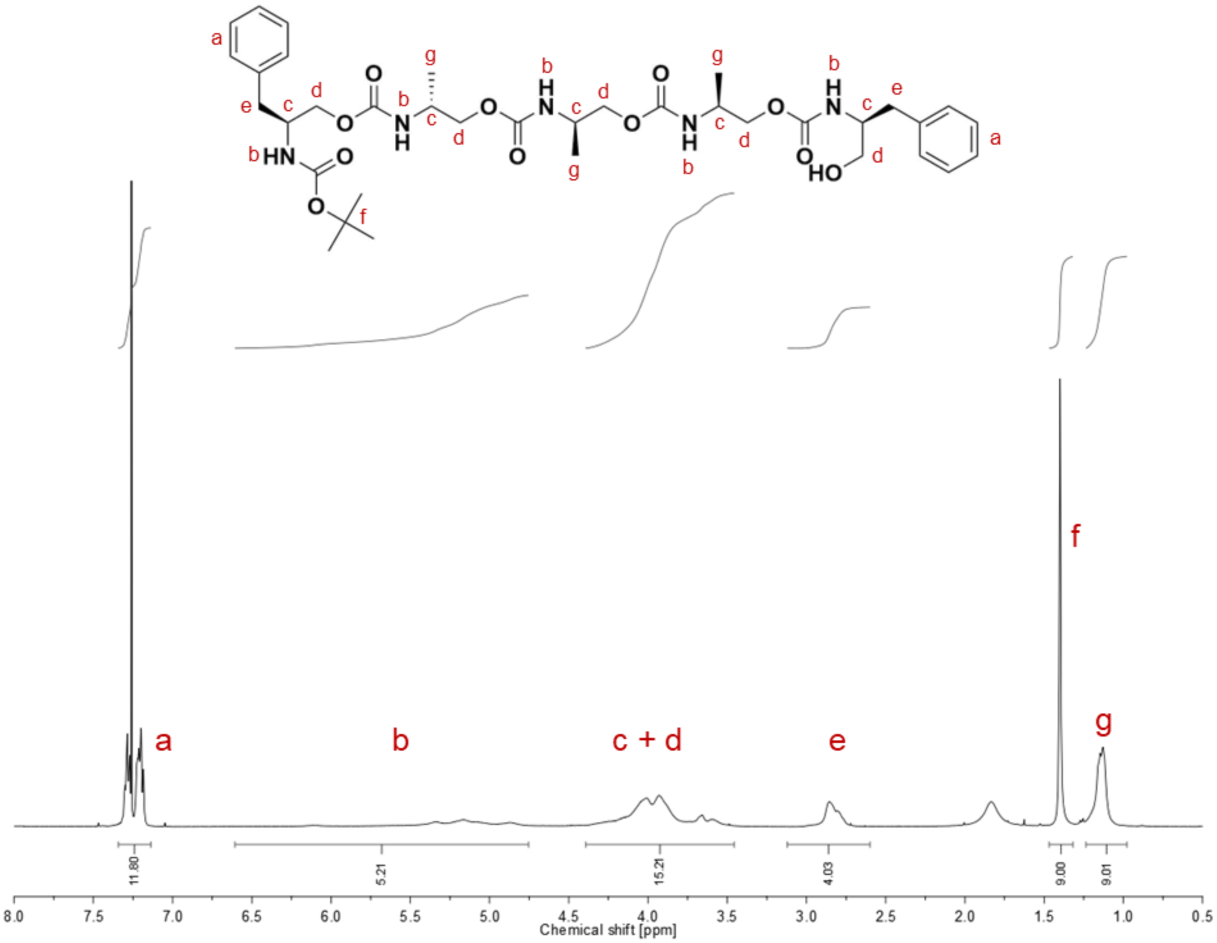

Figure S20. ^{1}H NMR spectrum of OU5 in $CDCl_3$, RT.

OU6 Boc-$P_SM_RM_SM_RP_S$

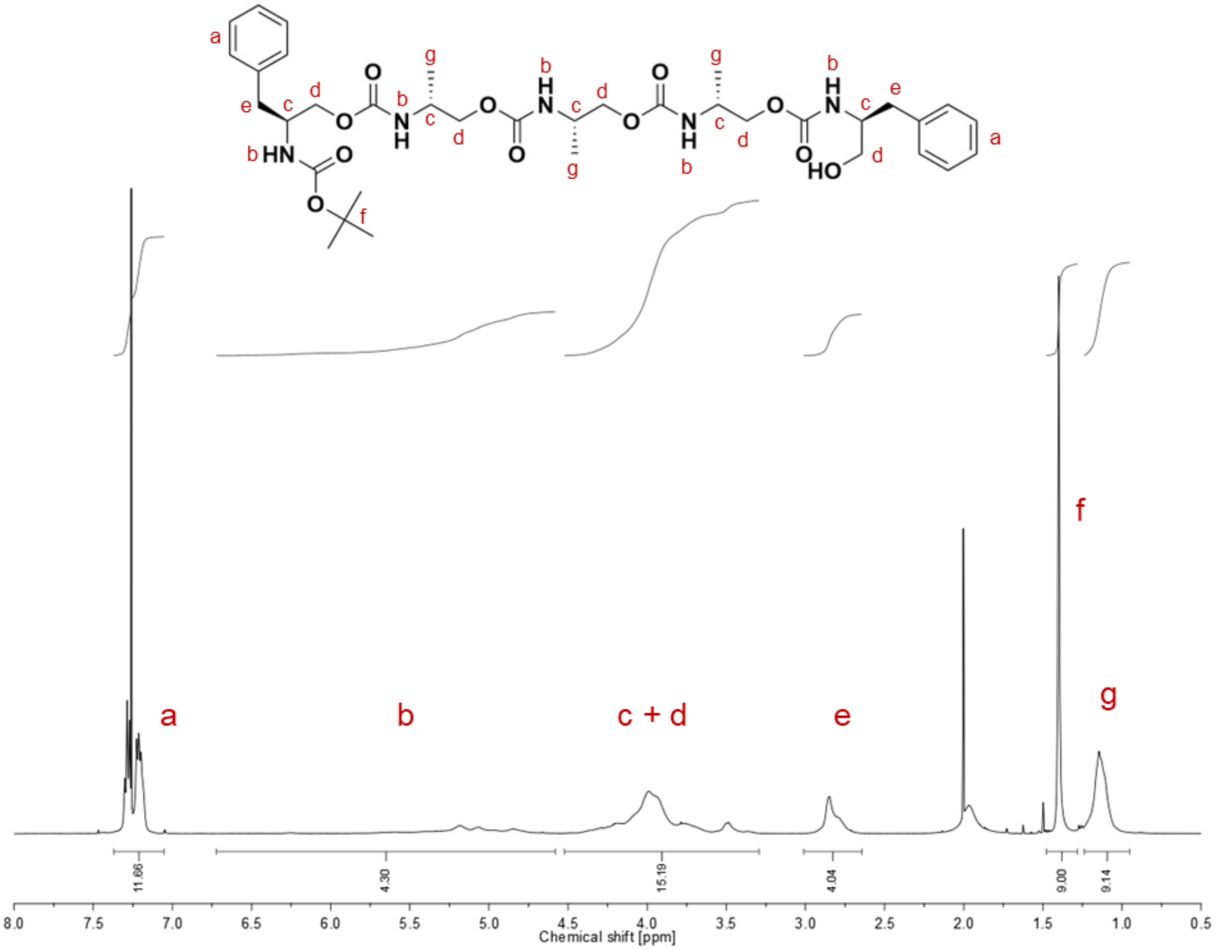

Figure S21. ^{1}H NMR spectrum of OU6 in $CDCl_3$, RT.

OU7 Boc-$P_SM_SM_RM_RP_S$

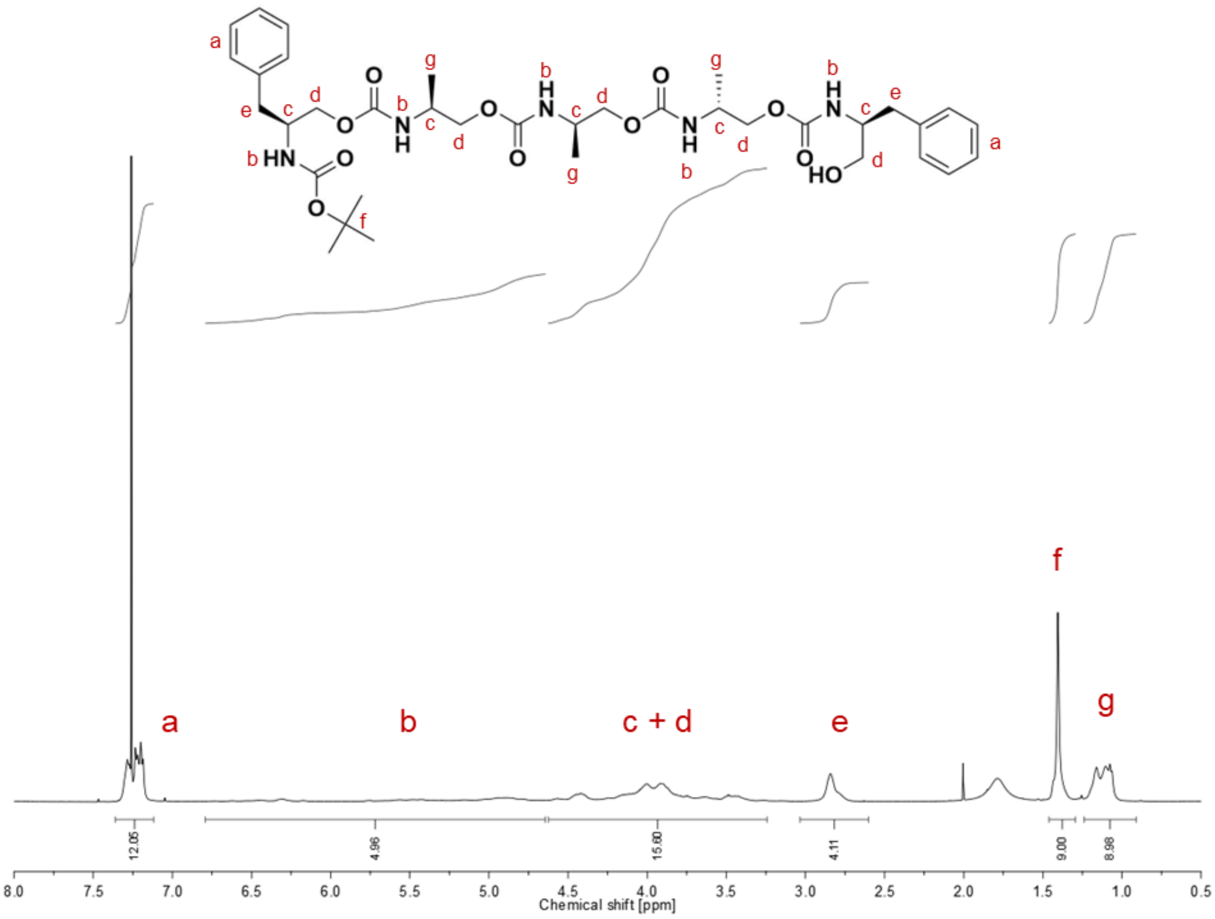

Figure S22. ^{1}H NMR spectrum of OU7 in $CDCl_3$, RT.

3.4. ^{1}H NMR VARIABLE TEMPERATURE

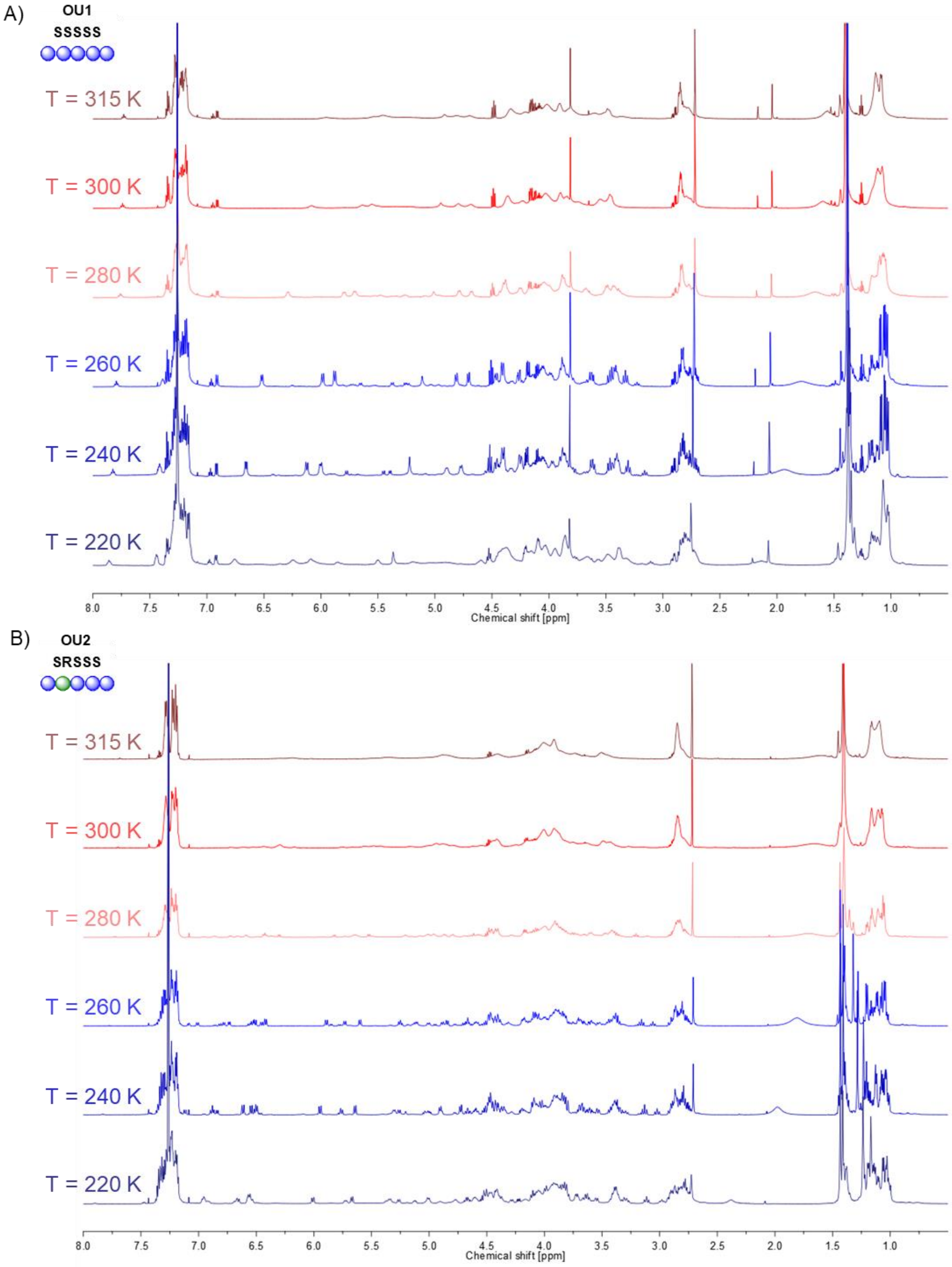

Figure S23. ^{1}H NMR variable temperature spectra at 220, 240, 260, 280, 300, and 315 K of (A) OU1 and (B) OU2 in $CDCl_3$, 6.8 mM.

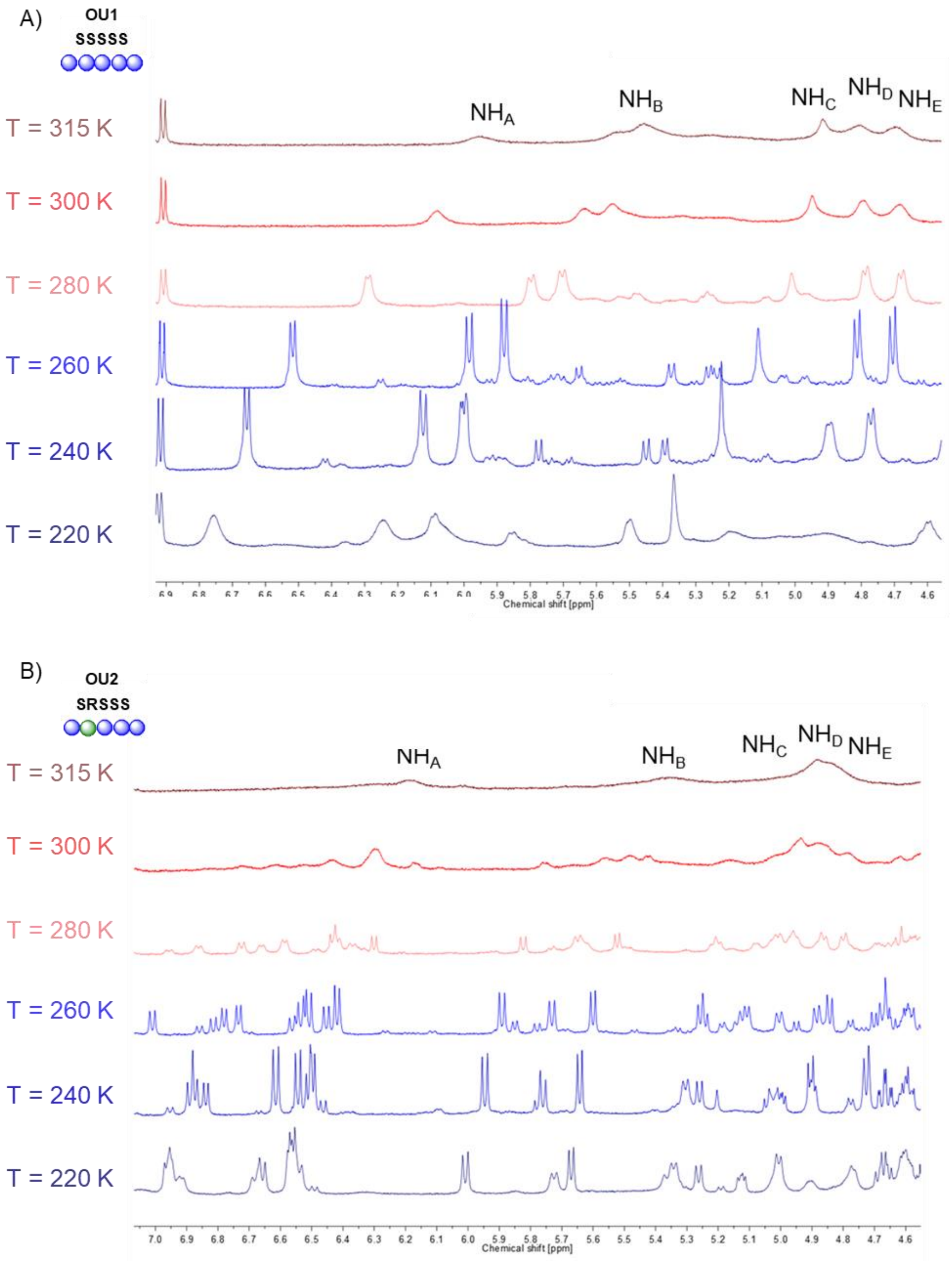

Figure S24. ^{1}H NMR variable temperature spectra at 220, 240, 260, 280, 300, and 315 K of (A) OU1 and (B) OU2 amine region, $CDCl_3$, 6.8 mM.

3.5. ¹H NMR VARIABLE CONCENTRATION

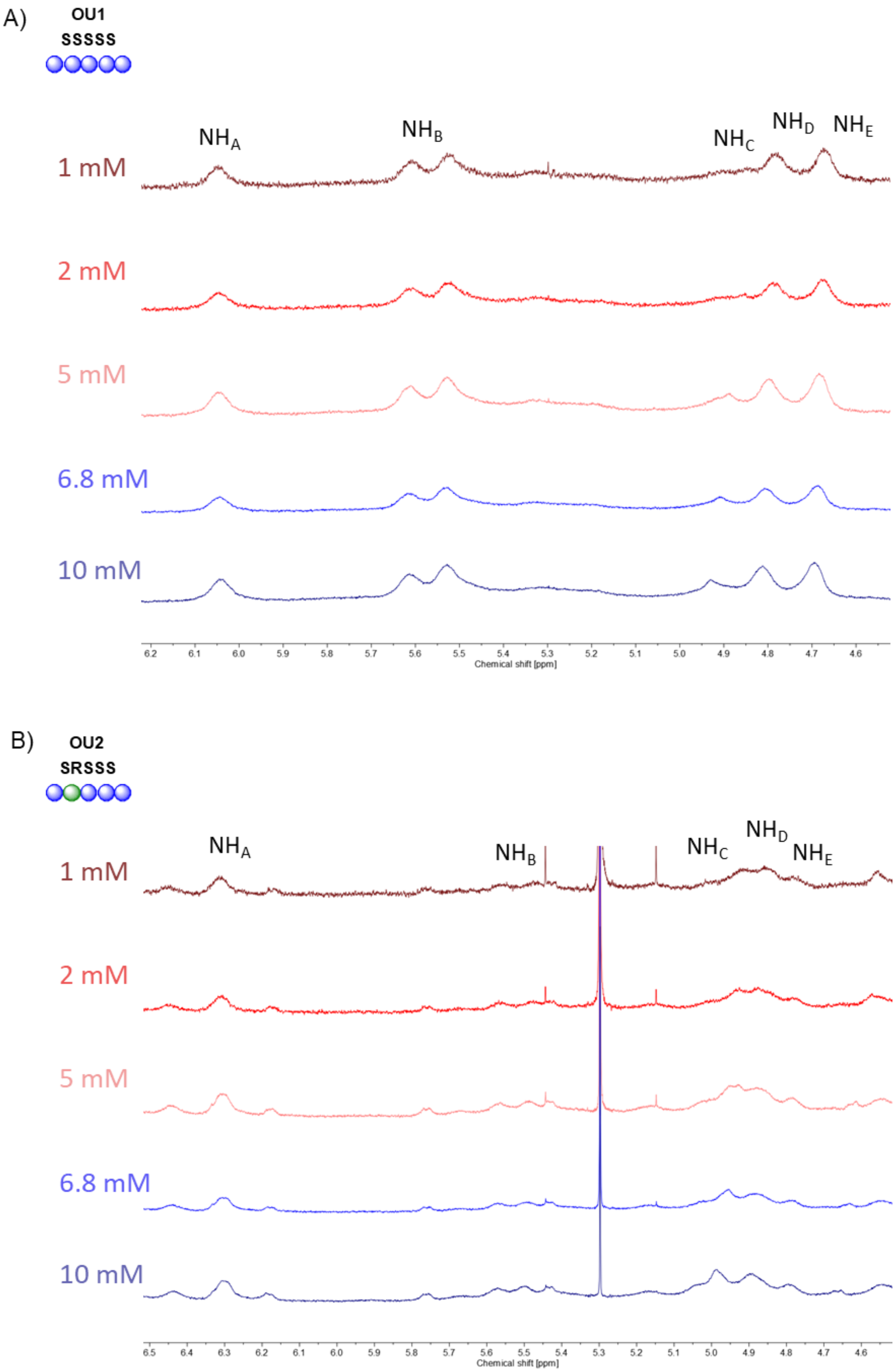

Figure S25. ¹H NMR variable concentration spectra for 1, 2, 5, 6.8, and 10 mM solutions of (A) OU1 and (B) OU2 amine region, $CDCl_3$, RT.

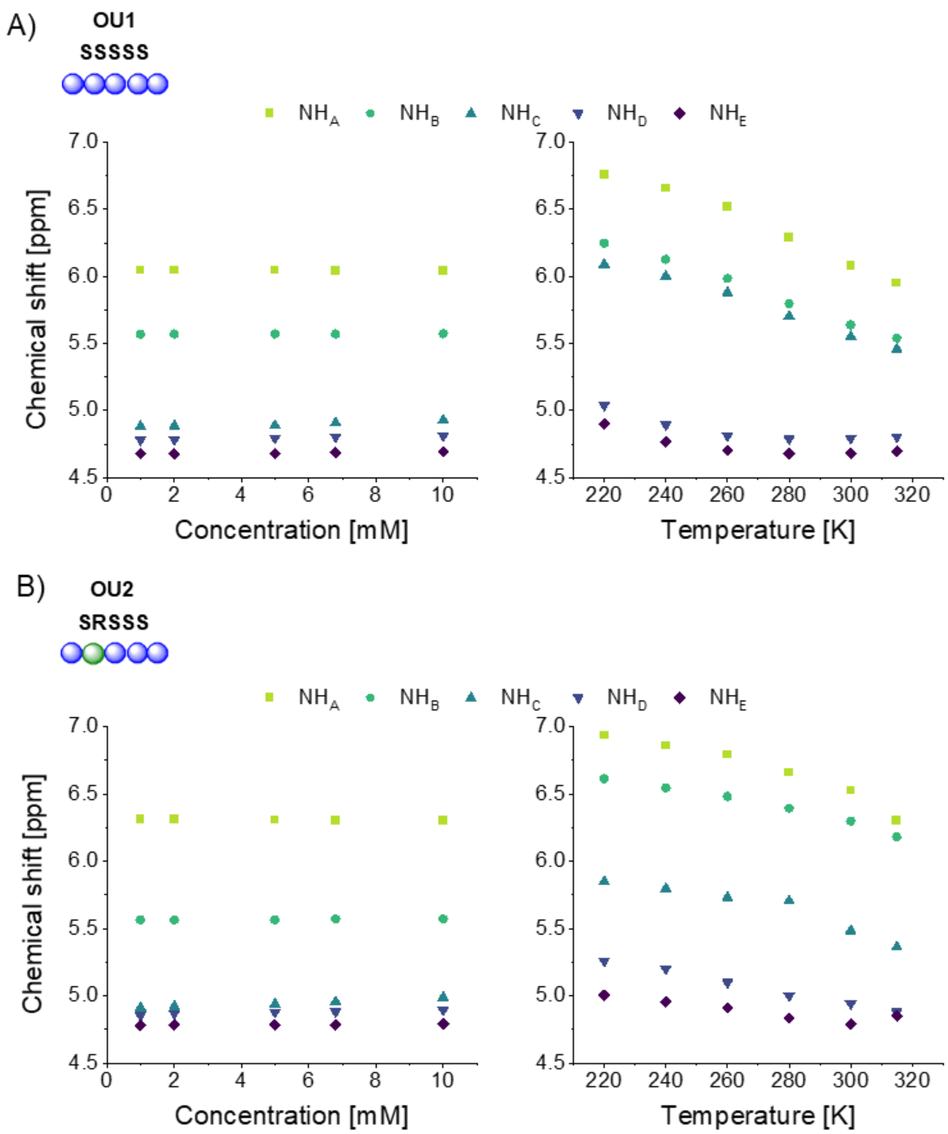

Figure S26. Changes in chemical shifts of N-H protons for (A) OU1 upon variable concentration (left) and temperature (right) and (B) OU2 upon variable concentration (left) and temperature (right). The N-H protons are labelled alphabetically from high to low chemical shifts. Change in chemical shift of N-H with temperature indicates the formation of hydrogen bonds. The lack of change in the N-H chemical shift upon the change in concentration suggests that present hydrogen bonds come from intramolecular interactions.

4. SIMULATIONS

4.1. STARTING GEOMETRY AND PARAMETRIZATION

3D geometries of sequences and BPA were drawn in the ChemSketch software[3], exported separately in the "mol" chemical format, converted to the "mol2" chemical format, and preliminarily optimized by OpenBabel software[4] using MMFF94s force field. After the initial molecular mechanics optimization, structures were submitted for the quantum mechanical structure optimization at an AM1 level of theory. The calculations were done using the Gaussian 2016 software[5]. Optimized structures were submitted to parametrization using the acpype Python package [6]. Acpype asks for several arguments; charge_method argument was set to "user" and the charges provided were the Mulliken charges[7] generated during QM structure optimization; "net_charge" was set to "0"; "multiplicity" was set to "1"; "atom_type" argument was set to "amber", which orders script to parametrize the molecule by AMBER14SB[8] force field. GROMACS input files were generated.

4.2. MULTIPLE SIMULATED ANNEALING – MOLECULAR DYNAMICS (MSA-MD)

Preparation, simulation, and analysis were done with the GROMACS 2018.4 software[9]. Generated GROMACS input geometry and topology files of oligourethane-BPA systems were modified to prepare them for simulations in implicit chloroform solvent. A single BPA molecule was inserted into the geometry files by "gmx_molecules" command, a "-box" option was set to "2" to ensure that the BPA molecule was close to a sequence. New topology files were created; both sequence and BPA molecule parameters were introduced; the AMBER03[10] implicit solvent parameters were used.

Geometries were optimized using two minimization methods: the steepest descent and the conjugate gradient (1000 steps each) in implicit solvent. The dielectric constant was set to "4.81", which refers to a chloroform solvent. Non-bonded interactions were described by the Coulomb potential and the Lennard-Jones potential with an infinite range neighbor search scheme. Periodic boundary conditions were switched off. The generalized Born formalism for implicit solvent was used. The Born radii cut-off was set to "0", and the OBC2 method was used to calculate the radii. Next, optimized structures were used and underwent a simulated annealing procedure, the

temperature was elevated from 298 K to 500 K within 50 ps, equilibrated over 30 ps, and ultimately reduced to 0 K within 70 ps, giving 150 ps of the total simulation time. The time step was equal to 0.001 ps. Nonbonded terms, PBC, and implicit solvent options were kept unchanged. All H-atom bonds are converted into a rigid constraint using the LINCS algorithm. The temperature was controlled by the Nosé–Hoover thermostat with the time coupling equal to 0.1 ps, and velocities were generated randomly using the Maxwell-Boltzmann distribution for the given temperature. The simulated annealing procedure was conducted 300 times, and the last frame of each trajectory was further used for a classical simulation of duration 10 ns and time step 0.002 ps in 298 K. The rest of the options remained the same. The procedure was repeated to obtain 300 trajectories, each consisting of 5001 frames.

A clustering was performed using the gromos[11] method. For the procedure 300 of 10 ns trajectories were merged and submitted for calculation (Fig. S27). The RMSD cut-off was set to “0.35 nm”, and hydrogen atoms were excluded. Only every 35th snapshot was used resulting in a sample size equal to 42866 snapshots. The algorithm generated pdb files containing multiple structures. One of them was composed of clusters of middle structures found in a single system sorted from the most populated to the least populated cluster and other pdb files were composed out of the population of a given cluster of that system. Only the 5 most populated clusters had their population generated in pdb files and that number of clusters was considered in further analysis.

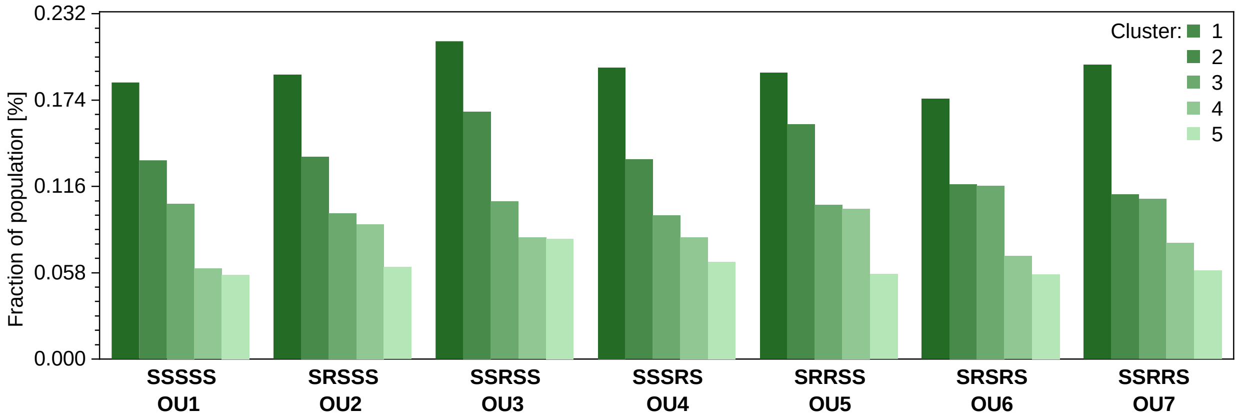

Figure S27. Cluster distributions for oligourethanes OU1-OU7 complexed with BPA. Cluster analysis was performed based on the structural similarity of BPA-oligomer complexes.

4.3. Structural analysis - Ramachandran plot

The Ramachandran plot method was used to analyze the structure of oligomers. In Table S3 we present analyzed torsional angles. Angles were read from cluster population pdb files generated by GROMACS after the clustering phase using a script written in Python. Ramachandran plots were generated with the seaborn module for Python[12]. The “joint grid” class was used to generate figures. It consists of 3 plots. The main one presents a mutual dependence of two variables and two subplots localized along the X and Y axis which show the distribution of X and Y variables, independently. The main plot and subplots are kernel density estimate type plots, in the case of the main plot “thresh”, “levels” and “bw_adjust” parameters were set to “0”, “100”, and “0.05” values accordingly. The top subplot had its “bw_adjust” parameter set to “0.2” whereas the side subplot had it set to “0.01”. On subplots, torsional angles were plotted regarding the monomer unit they describe, whilst the main plots are plotted with no distinction of monomer units. Counting monomer units starts with the Boc-protected monomer (N-terminus). The first monomer, as well as the last monomer, are omitted due to the lack of torsion-defining atoms presented in the previously mentioned table. In the upper left corner of the figures, the codenames of a sequence and the number of clusters are printed.

Table S3. Atoms of monomer that were used to define torsions.

<table>
<tr><td rowspan="6">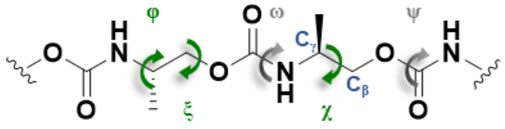
</td><th>Angle</th><th>Defining bond</th></tr>
<tr><td>φ</td><td>$N–C_γ$</td></tr>
<tr><td>ξ</td><td>$C_β–O$</td></tr>
<tr><td>χ</td><td>$C_γ–C_β$</td></tr>
<tr><td>ψ</td><td>C–O</td></tr>
<tr><td>ω</td><td>C–N</td></tr>
</table>

From now on, 4 representative cases will be examined: OU4-3, OU5-1, OU6-5, and OU7-1 (Fig. S28). The OU4-3 conformation resembles an unwinding helix starting from its C-terminus; The OU6-5 structure is a helix with the BPA inserted between one of its turns; OU5-1 and OU7-1 are examples of the random coil. They are found to adopt a distinctive conformation and may serve as an example of structure investigation of urethanes using the Ramachandran plot and its modifications. The first pair of torsions φ and ψ are presented in Fig. S28. which corresponds to

the α-peptide case. According to other systems β- or γ-peptides, φ and ψ should adopt negative or positive values. Such behavior is present in φ angle, which adapts values -120° +/- 40° if the monomer unit is an S isomer and 120° +/- 40° if otherwise. In the case of ψ angle, such a peculiarity is absent. It is a result of a pseudo double bond between O and C, which restricts that angle to a single 180° value. As a result, it provides no relevant structural insights since one of the two variables is constant.

To overcome that limitation, other angles were taken into consideration, namely ξ and χ, and modified Ramachandran plots are presented in Fig. S28-S32. The ω is also limited to 180° values for the same reason as ψ, therefore it is not considered. ξ and χ have different angle preferences, the prior tends to populate two angles +/- 50° whereas the latter has four preferences: +/-180°, +/-90°. A closer investigation of the φ vs ξ plots reveals a limited dependence of the modified Ramachandran plot on the conformation of macromolecules. The ξ torsion is distributed in two ranges 50° and -50° and φ vs ξ plot correctly determines most of the disordered (OU5-1 and OU7-1) and ordered (OU4-3) structures, yet it fails in the case of the OU6-5, where it gives an ambiguous result. Therefore, analysis of another angle is required. The torsion χ is more sensitive to the macromolecule structure; torsions in modified Ramachandran plots populating predominantly I and II quadrants present a helix-like structure (OU4-3 and OU6-5), whereas a random coil populates multiple spots in similar ratios (OU5-1 and OU7-1). Together, φ, ξ and χ portray the wellness of the structure. Occupation of multiple angles reflects the randomness of the structure, a single angle preference led to an organization of conformation.

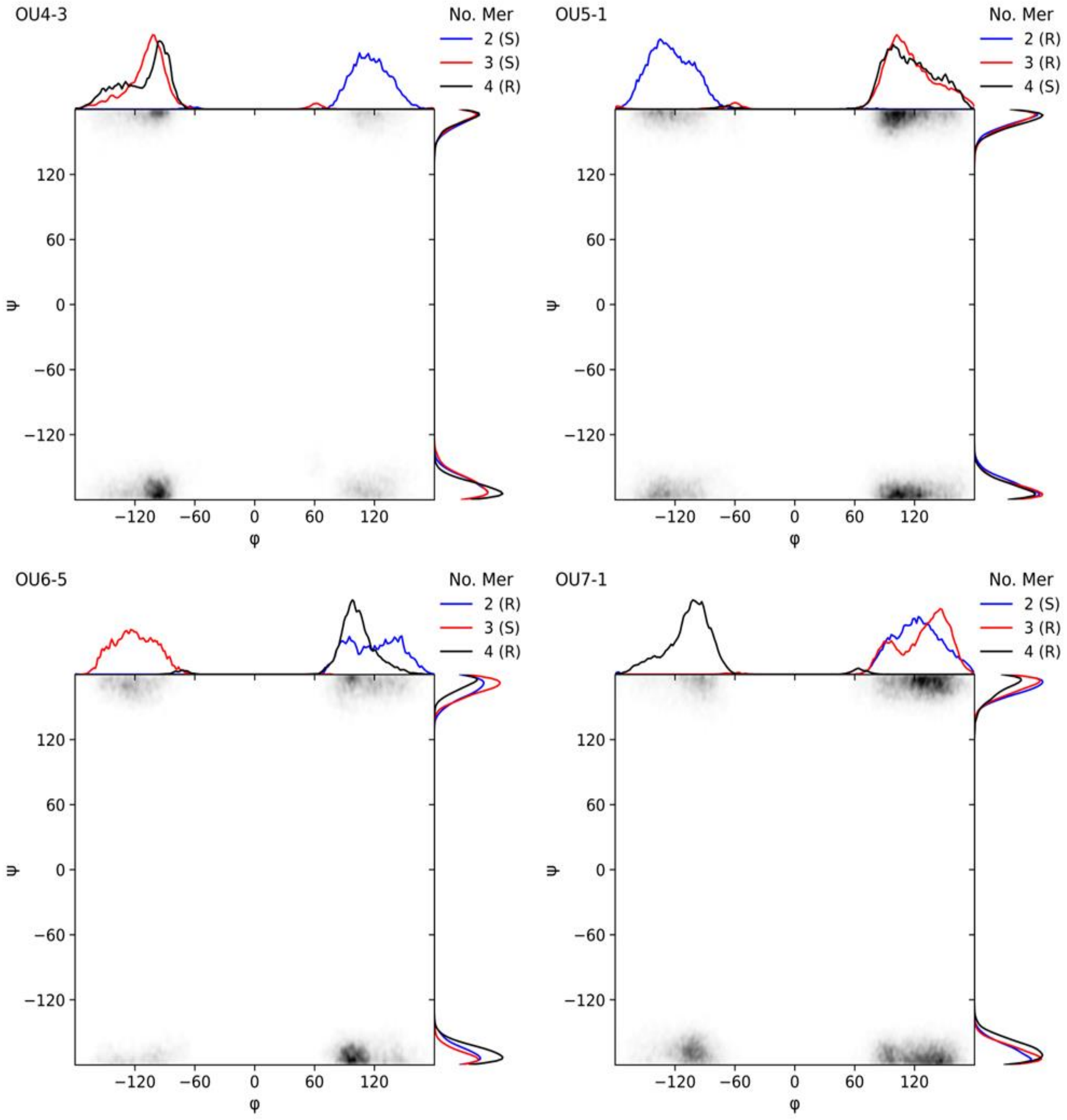

Figure S28. Ramachandran plots of OU4-3, OU5-1, OU6-5 and OU7-1.

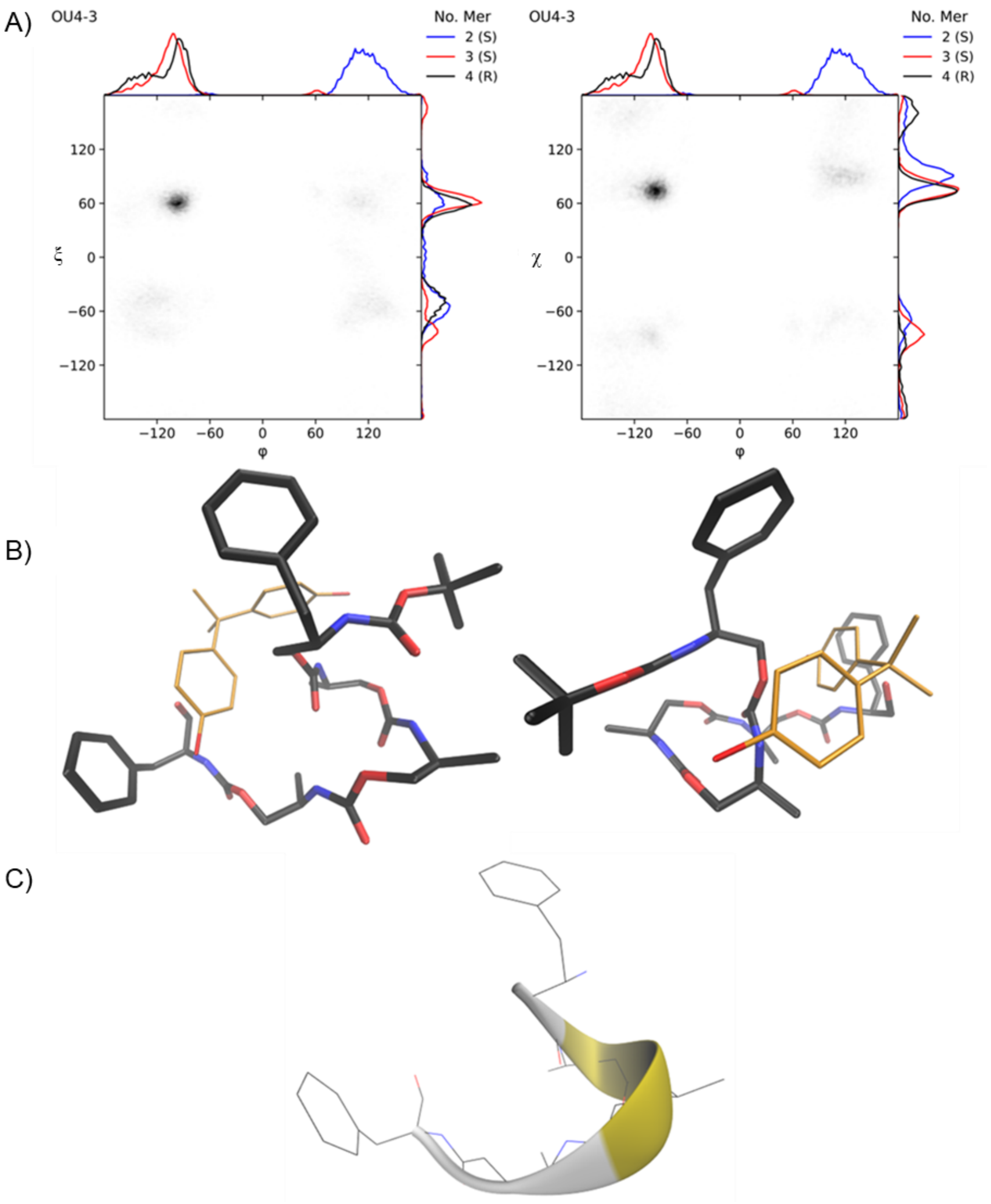

Figure S29. A) Modified Ramachandran plots of OU4-3. B) Structures of OU4-3 C) Structure of OU4-3 drawn in NewCartoon style.

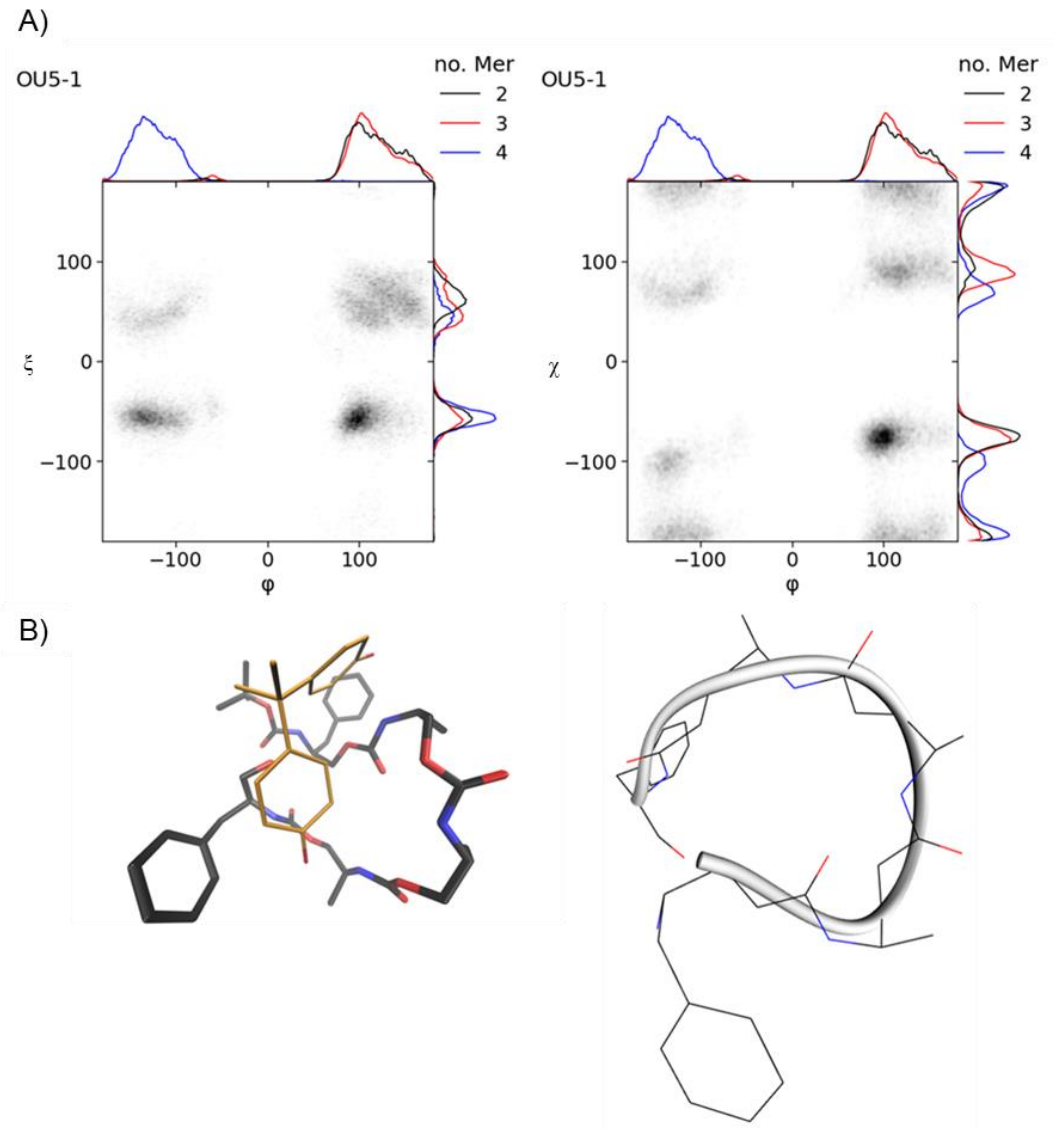

Figure S30. A) Modified Ramachandran plots of OU5-1. B) Structures of OU5-1 in CPK (left) and NewCartoon (right) styles.

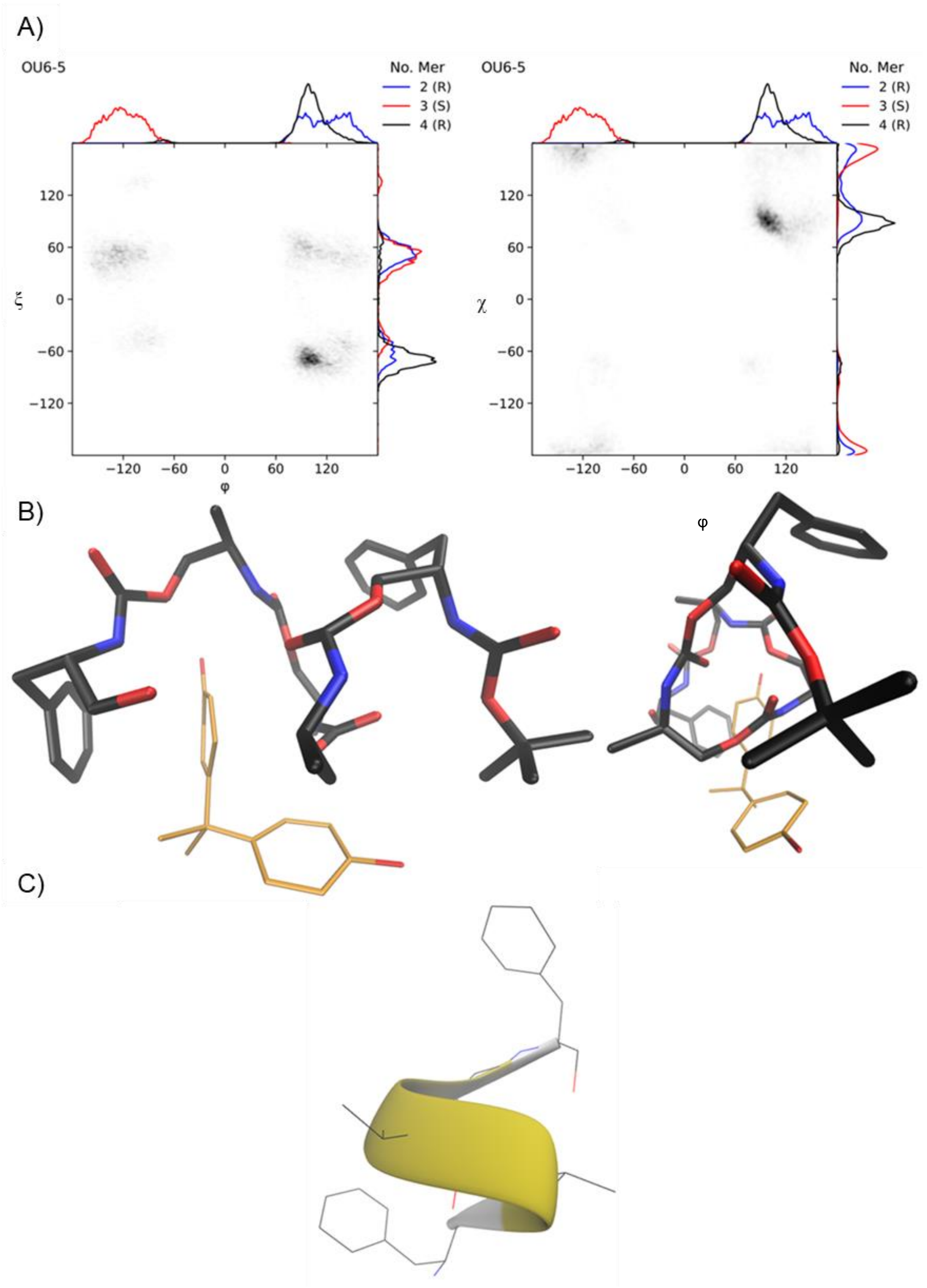

Figure S31. A) Modified Ramachandran plots of OU6-5. B) Structures of OU6-5 C) Structure of OU6-5 drawn in NewCartoon style.

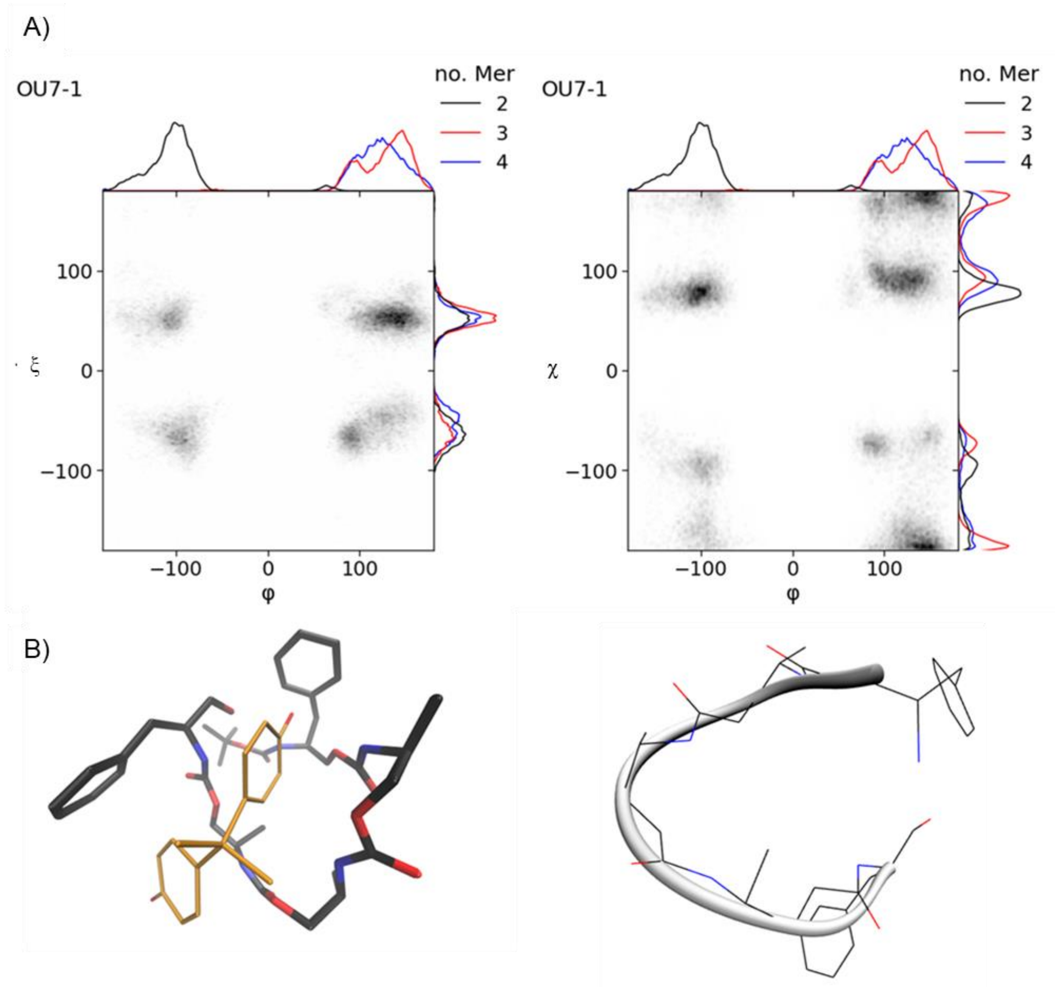

Figure S32. A) Modified Ramachandran plots of OU7-1. B) Structures of OU7-1 in CPK (left) and NewCartoon (right) styles.

4.4. FORCE FIELD COMPARISON

Simulations were carried out using the GAFF[13] force field. GAFF was tested in parallel with AMBER14SB to assess whether a more general force field would be a better fit for our systems. The only change was the parameterization by the force field.

GAFF simulations will be examined by a φ vs ξ plot of OU1-1 (Fig. S33). The structure obtained using the GAFF force field was rejected by alarming φ angle distribution, which shape might be a broad irregular bump (3rd monomer, red line) or a separated distribution maximum (4th monomer, black line). Similar tendencies were spotted in β-peptide simulations [14]. The unphysical behavior may be a result of a bias on the torsional angle preferences posed by GAFF.

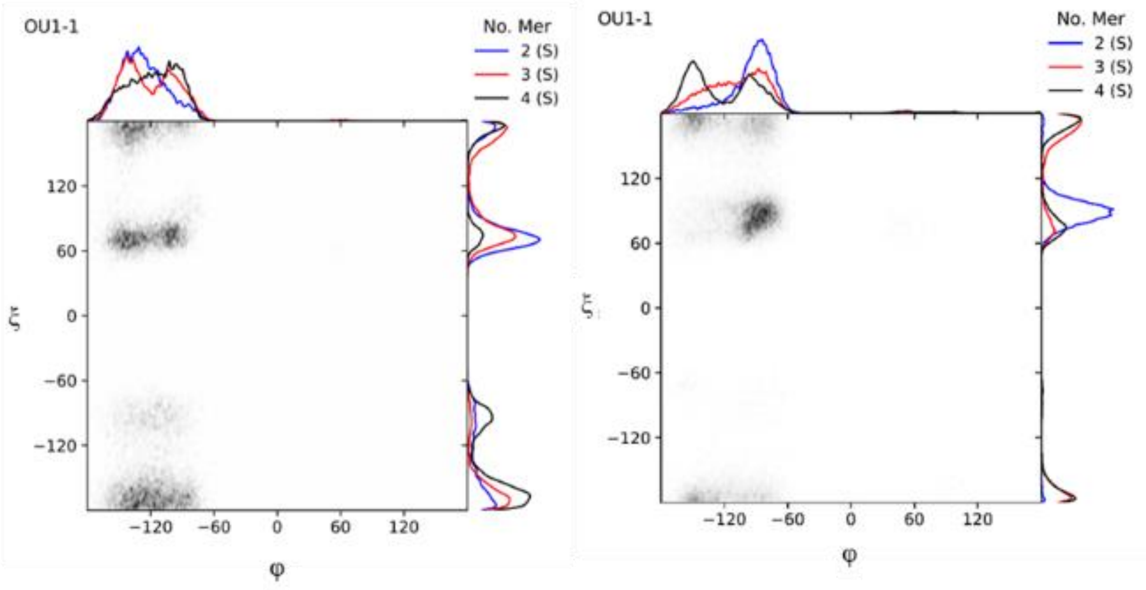

Figure S33. Modified Ramachandran plot of OU1-1 simulated with AMBER14SB (left) and GAFF (right).

4.5. MOLECULAR MECHANICS GENERALIZED BORN SURFACE AREA (MMGBSA)

Two types of ensembles were prepared for this phase. An ensemble of conformations from the population of a single cluster of a complex to calculate an average over the cluster, and an ensemble of 105th frames of merged 10 ns trajectories to calculate an average over the whole sampled configurational space of a complex.

Free energy of binding was assessed with gmx_MMPBSA with the single trajectory method[15].To calculate energetical terms of the intermolecular interaction the same force field has been used as in previous procedures. The Generalized Born method to use was the second model of OCB. All frames of the ensemble were submitted to the calculation of binding energy, and other general and implicit solvent options have been left unchanged. Free energies of binding (ΔG_{bind}) calculated for complexes simulated in $CHCl_3$ are depicted in Fig. S34. An average ΔG_{bind} and its terms are summarized in Table S4. In the case of normal mode calculation (nmode) options where the following: convergence criteria were set to 0.01 and maximum cycles of minimization were set to 500. General and implicit solvent options were the same as in the previous calculation. Due to the higher cost of calculation, only 1/10 of the frames of the submitted ensembles were submitted to this procedure.

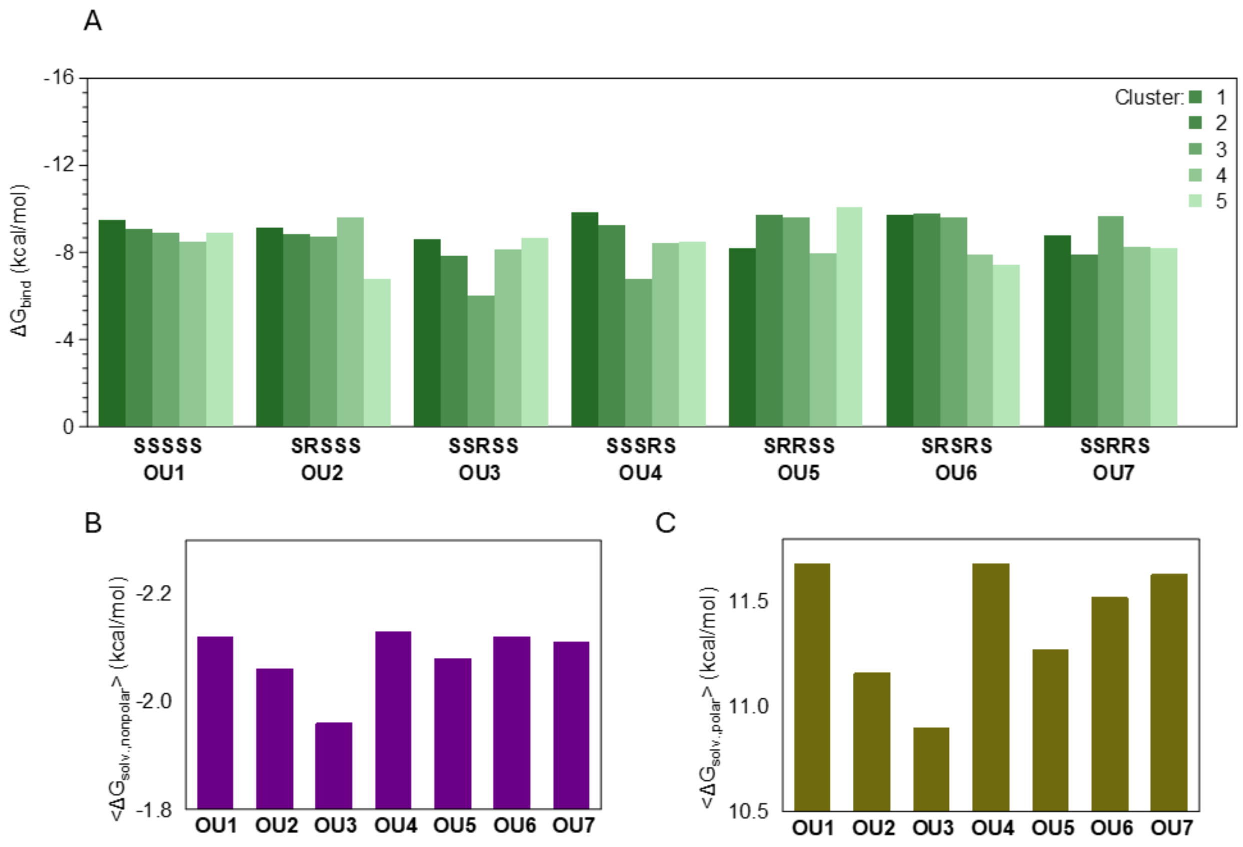

Figure S34. A) Free energies of binding (ΔG_{bind}) calculated for clusters of oligomer-BPA complexes simulated in $CHCl_3$. B) The ensemble average free energies of solvation ($\Delta G_{solv.nonpolar}$) calculated for oligomer-BPA complexes simulated in $CHCl_3$. B) The ensemble average free energies of solvation ($\Delta G_{solv.polar}$) calculated for oligomer-BPA complexes simulated in $CHCl_3$.

Table S4. Free energies of binding and its terms (in kcal/mol) calculated for complexes simulated in $CHCl_3$.

	ΔE_{VdW}	+/-	ΔE_{el}	+/-	$\Delta G_{solv.polar}$	+/-	$\Delta G_{solv.nonpolar}$	+/-	ΔG_{bind}	+/-	ΔG_{bind} + Nmode	+/-
**OU1 SSSSS**	-15.04	3.87	-2.89	2.14	11.68	1.92	-2.11	0.3	-8.36	3.94	3.55	4.82
**OU2 SRSSS**	-14.77	3.75	-2.93	2.12	11.11	1.87	-2.05	0.32	-8.64	3.74	3.35	4.62
**OU3 SSRSS**	-13.81	3.72	-2.8	1.99	10.87	2.02	-1.96	0.31	-7.70	3.86	4.27	4.76
**OU4 SSSRS**	-15.1	3.75	-3.1	2.17	11.63	1.96	-2.13	0.32	-8.70	3.79	3.16	4.57
**OU5 SRRSS**	-14.69	3.67	-2.92	2.05	11.18	1.83	-2.07	0.32	-8.50	3.77	3.42	4.62
**OU6 SRSRS**	-14.83	3.94	-3.09	2.16	11.51	1.93	-2.12	0.34	-8.54	4.06	3.37	4.88
**OU7 SSRRS**	-14.98	4.00	-2.91	2.22	11.63	1.99	-2.13	0.33	-8.37	4.00	3.64	4.84

A magnitude of error in the calculated entropy value being virtually equal to its paired value makes entropy calculations not reliable, therefore no entropy correction was applied.

5. NMR ANALYSIS OF OLIGOMER-BPA COMPLEX

5.1. NUCLEAR OVERHAUSER EFFECT SPECTROSCOPY (NOESY)

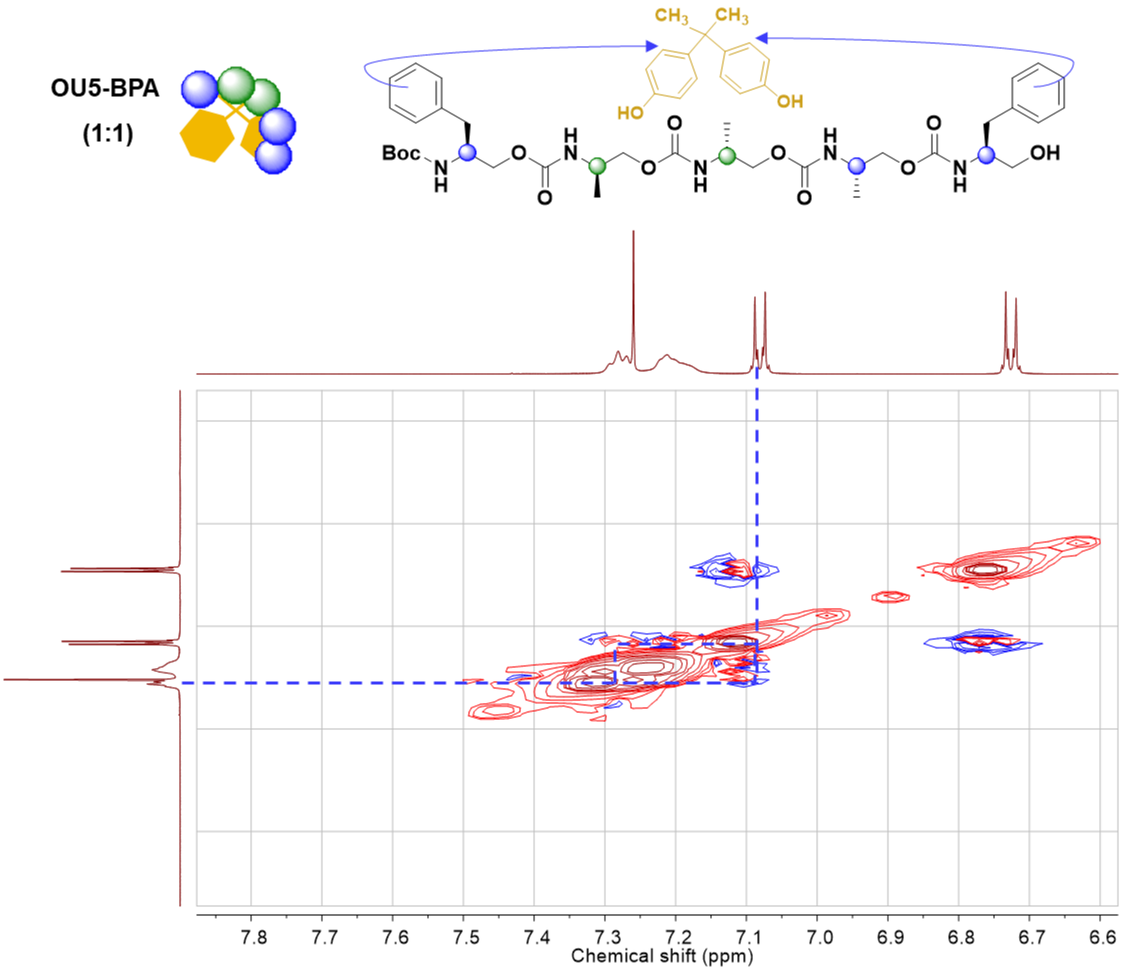

Figure S35. NOESY spectrum of OU5-BPA complex in $CDCl_3$, RT, 6.8 mM. In the spectrum, characteristic cross-couplings that can come from spatial proximity of phenyl rings from oligomer and BPA are observed at the aromatic range.

5.2. ¹H NMR OF OLIGOMERS-BPA

OU1 Boc-$P_SM_SM_SM_SP_S$:BPA, 1:1 molar ratio

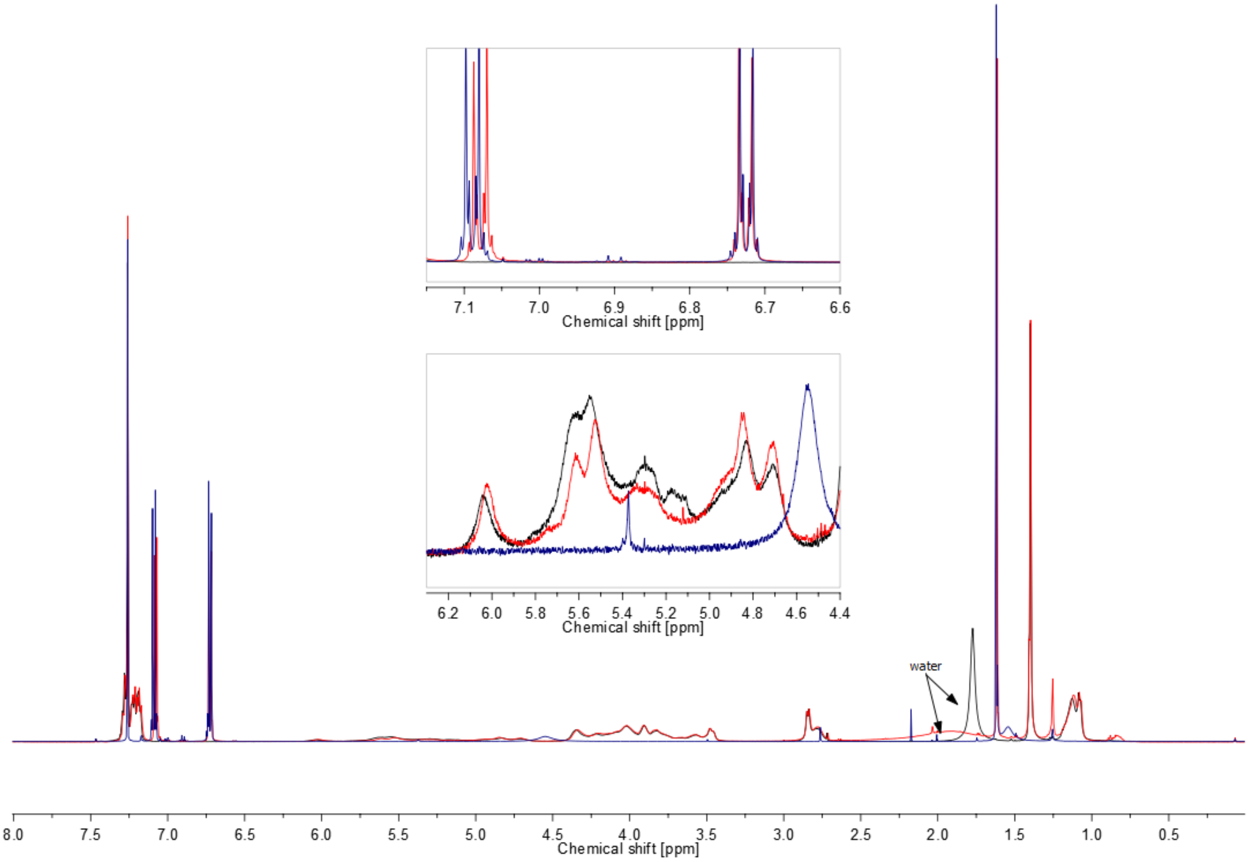

Figure S36. Superposition of ^{1}H NMR spectra: Black: OU1; Red: OU1 with BPA; Blue: BPA, $CDCl_3$, RT, 6.8 mM.

OU2 Boc-$P_SM_RM_SM_SP_S$:BPA, 1:1 molar ratio

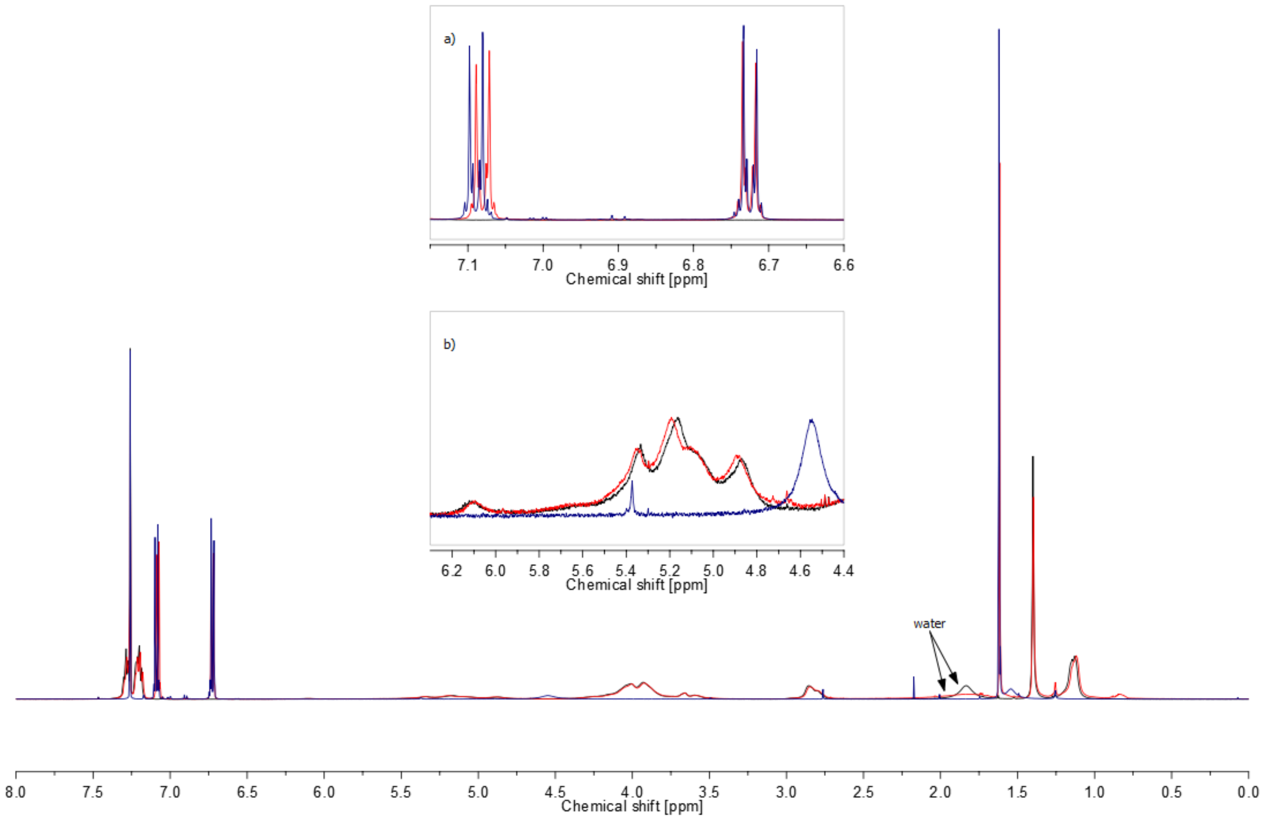

Figure S37. Superposition of ^{1}H NMR spectra: Black: OU2; Red: OU2 with BPA; Blue: BPA, $CDCl_3$, RT, 6.8 mM.

OU3 Boc-$P_SM_SM_RM_SP_S$:BPA, 1:1 molar ratio

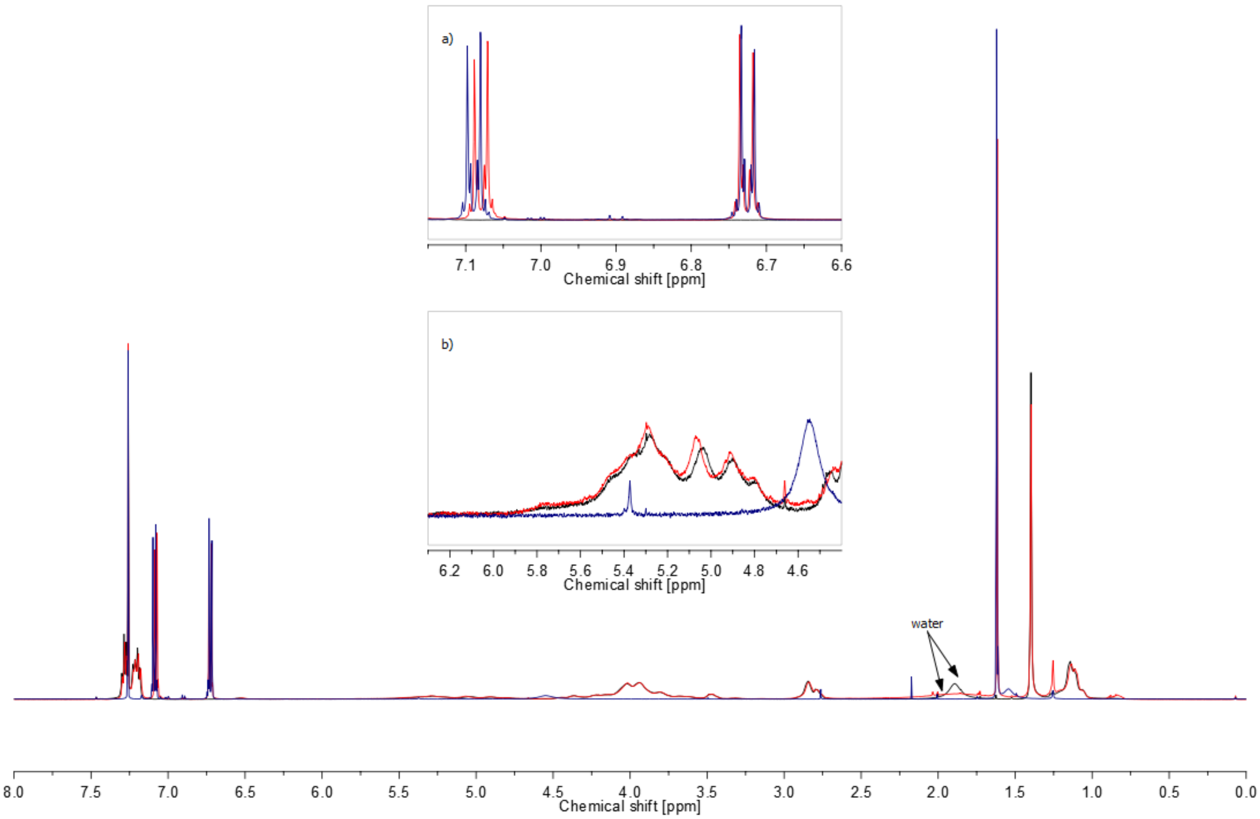

Figure S38. Superposition of ^{1}H NMR spectra: Black: OU3; Red: OU3 with BPA; Blue: BPA, $CDCl_3$, RT, 6.8 mM.

OU4 Boc-$P_SM_SM_SM_RP_S$:BPA, 1:1 molar ratio

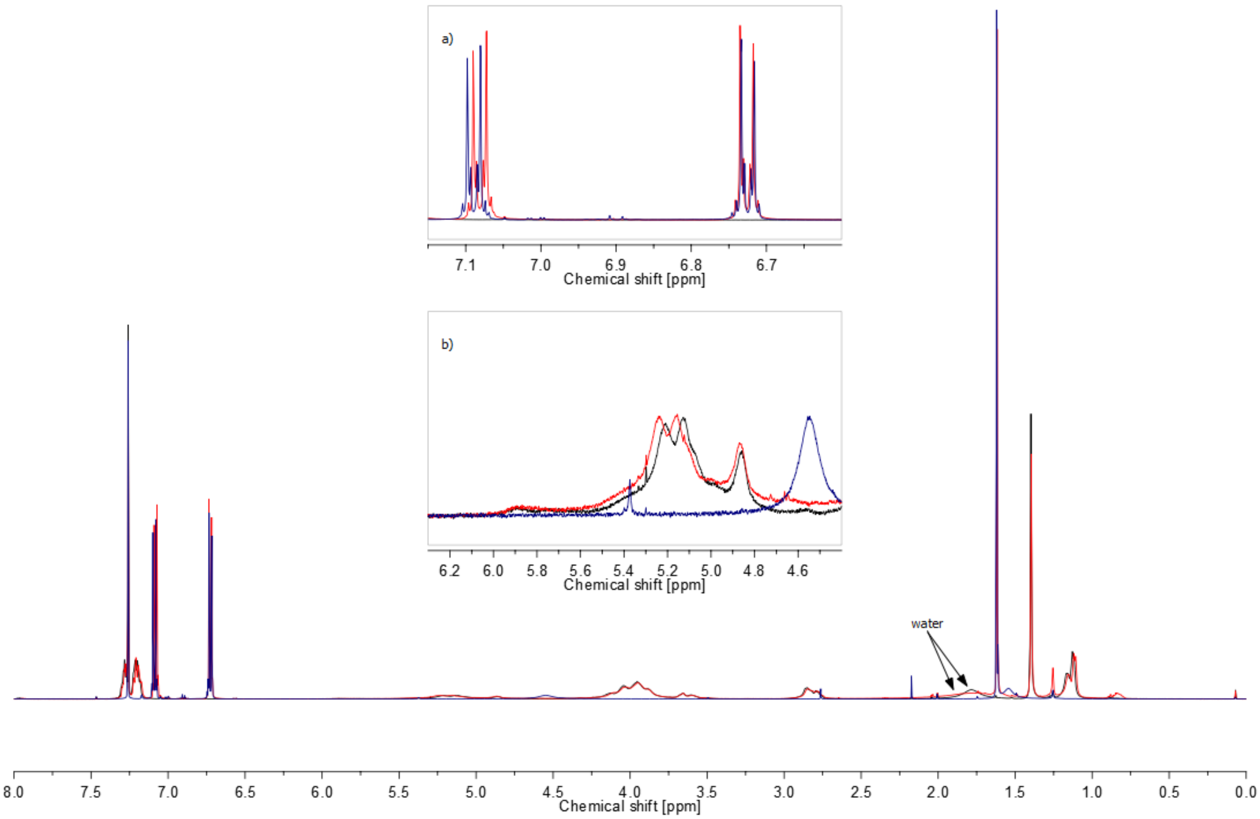

Figure S39. Superposition of ^{1}H NMR spectra: Black: OU4; Red: OU4 with BPA; Blue: BPA, $CDCl_3$, RT, 6.8 mM.

OU5 Boc-$P_SM_RM_RM_SP_S$:BPA, 1:1 molar ratio

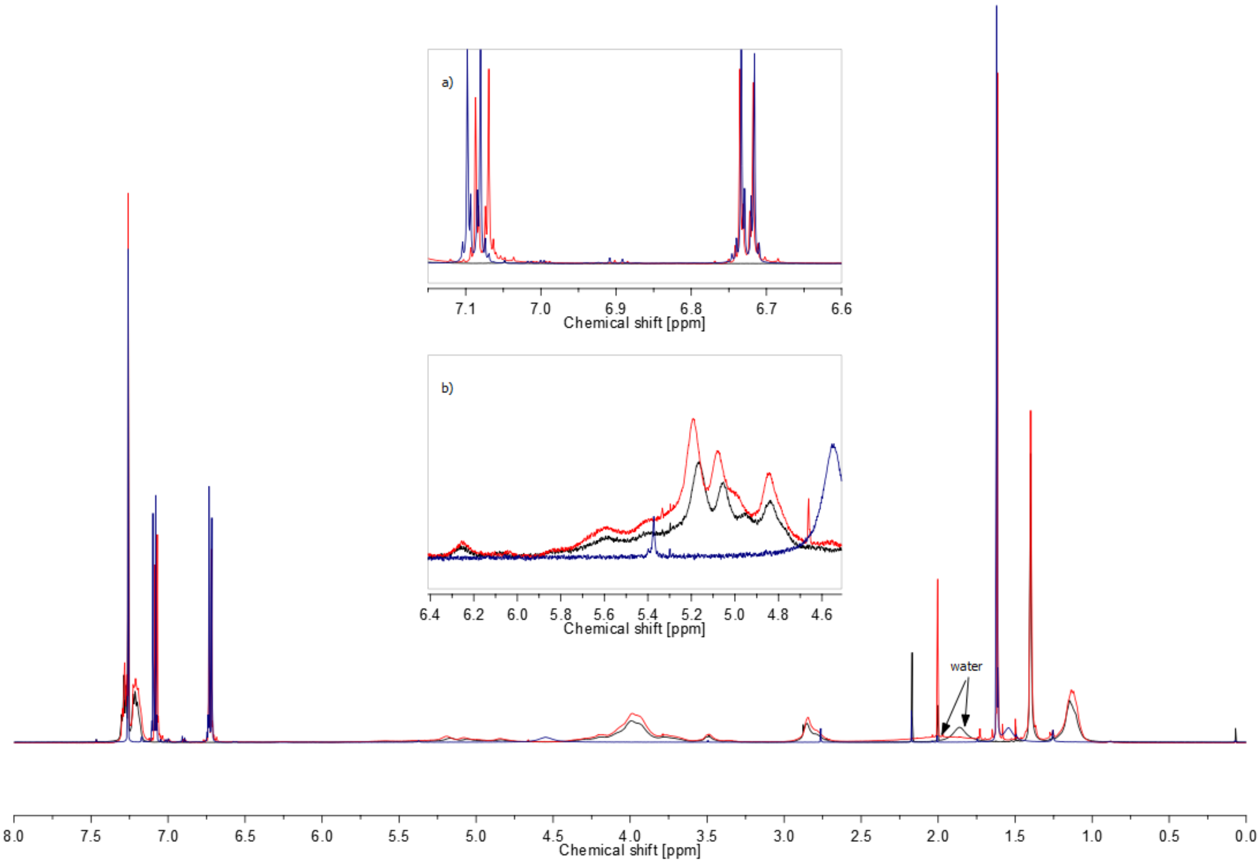

Figure S40. Superposition of 1H NMR spectra: Black: OU5; Red: OU5 with BPA; Blue: BPA, $CDCl_3$, RT, 6.8 mM.

OU6 Boc-$P_SM_RM_SM_RP_S$:BPA, 1:1 molar ratio

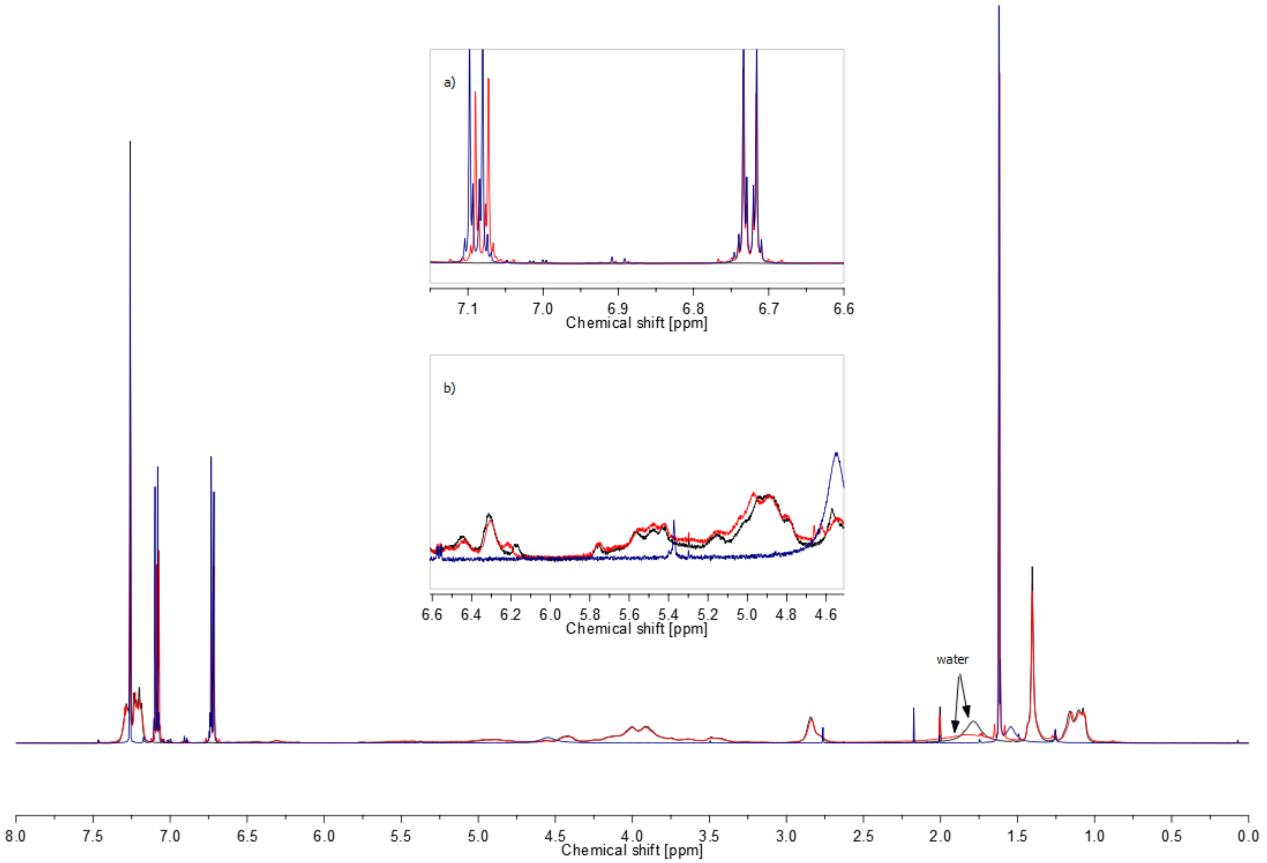

Figure S41. Superposition of 1H NMR spectra: Black: OU6; Red: OU6 with BPA; Blue: BPA, $CDCl_3$, RT, 6.8 mM.

OU7 Boc-P$_S$M$_S$M$_R$M$_R$P$_S$:BPA, 1:1 molar ratio

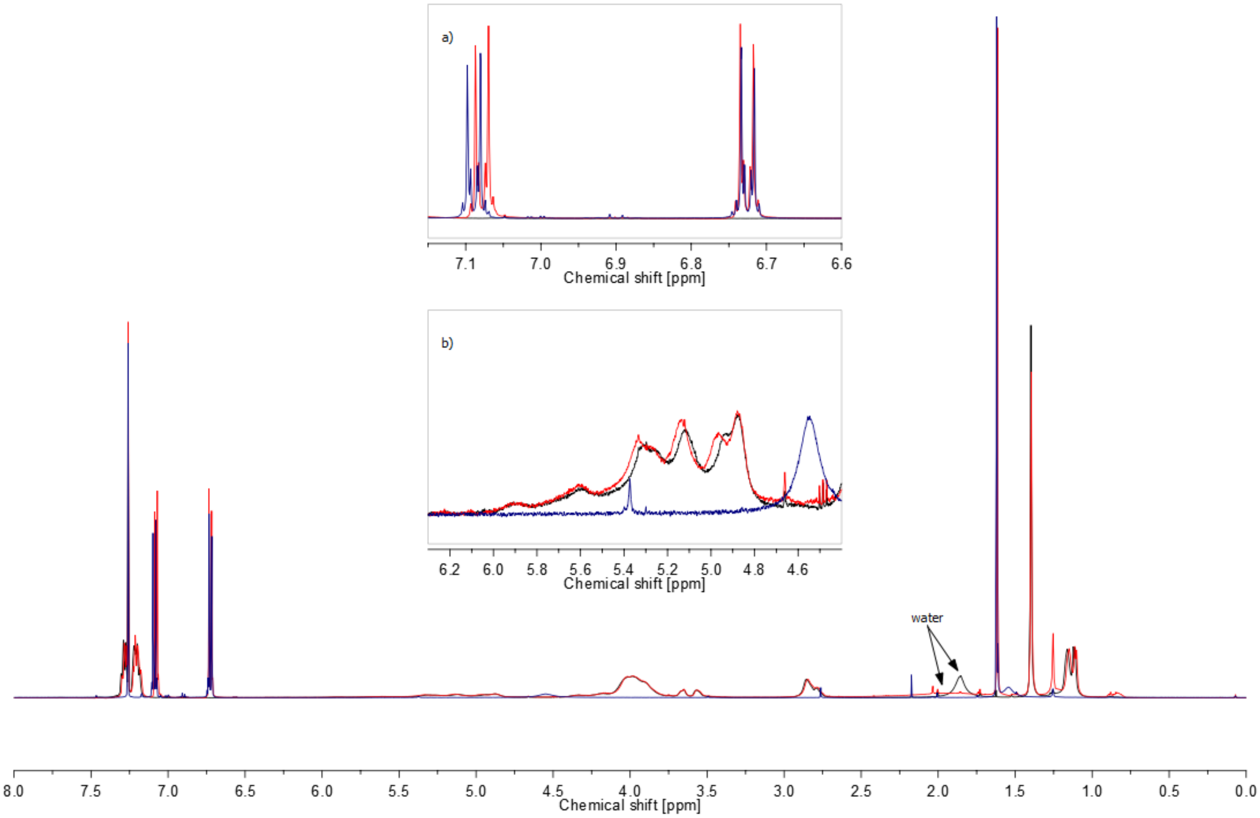

Figure S42. Superposition of ^{1}H NMR spectra: Black: OU7; Red: OU7 with BPA; Blue: BPA, $CDCl_3$, RT, 6.8 mM.

Table S5. Changes of the chemical shift of the BPA doublet at 7.1 ppm in the presence of oligomer.

Oligomer	Chemical shift difference (ppm)
**OU1** Boc-$P_SM_SM_SM_SP_S$	0.011
**OU2** Boc-$P_SM_RM_SM_SP_S$	0.009
**OU3** Boc-$P_SM_SM_RM_SP_S$	0.010
**OU4** Boc-$P_SM_SM_SM_RP_S$	0.009
**OU5** Boc-$P_SM_RM_RM_SP_S$	0.011
**OU6** Boc-$P_SM_RM_SM_RP_S$	0.008
**OU7** Boc-$P_SM_SM_RM_RP_S$	0.010

6. LIGAND TITRATION EXPERIMENTS

6.1. CIRCULAR DICHROISM ANALYSES

OU1 Boc-$P_SM_SM_SM_SP_S$

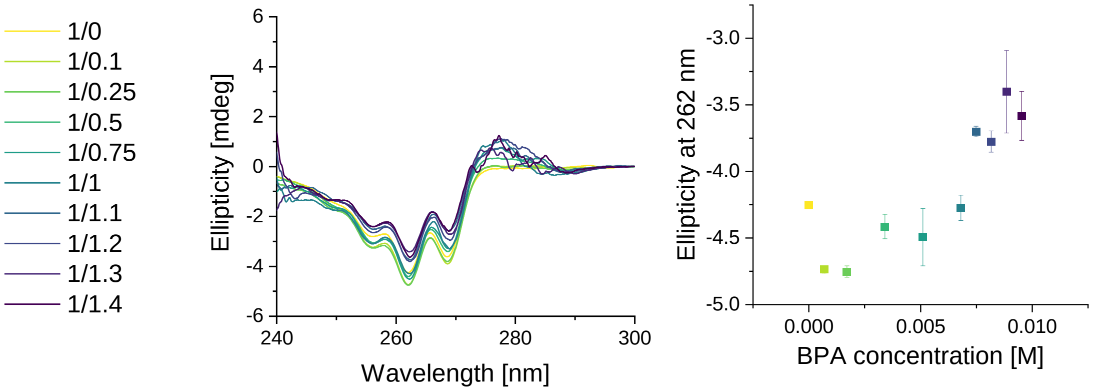

Figure S43. CD spectra of OU1 6.8 mM in $CHCl_3$ during BPA titration (left) and change in the CD signal intensity of OU1 at 262 nm depending on BPA concentration (right).

OU2 Boc-$P_SM_RM_SM_SP_S$

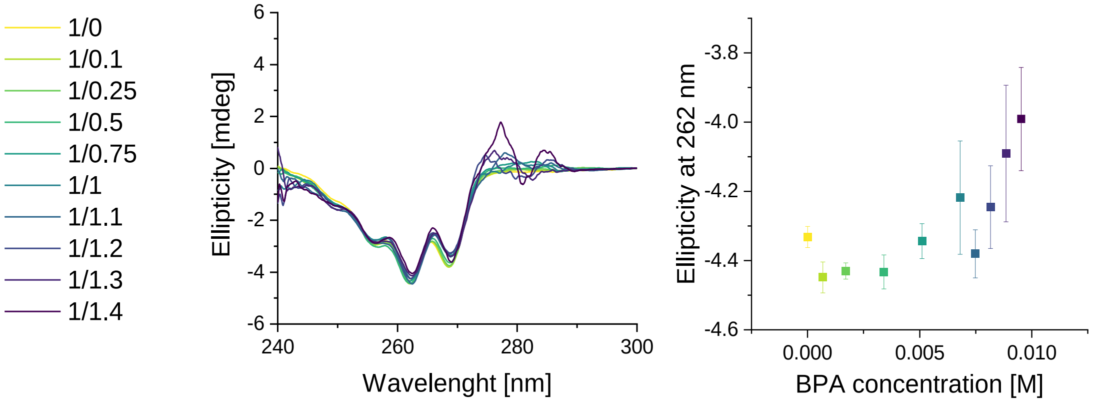

Figure S44. CD spectra of OU2 6.8 mM $CHCl_3$ during BPA titration (left) and change in the CD signal intensity of OU2 at 262 nm depending on BPA concentration (right).

OU3 Boc-$P_SM_SM_RM_SP_S$

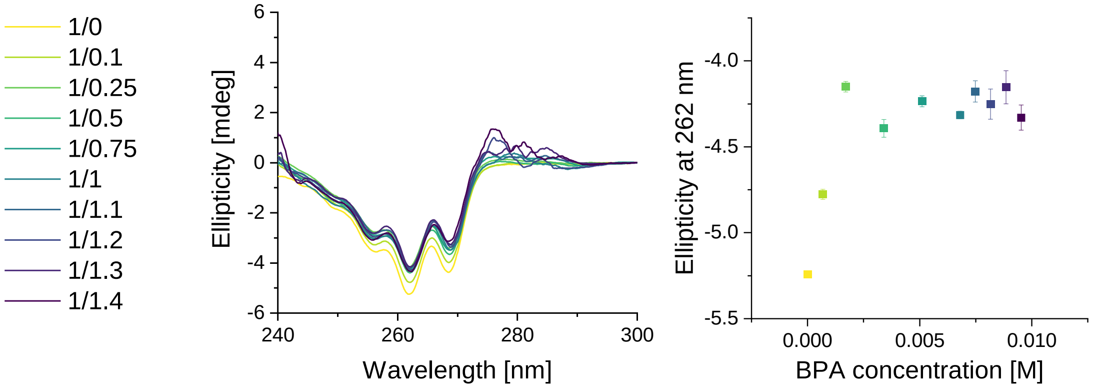

Figure S45. CD spectra of OU3 6.8 mM in $CHCl_3$, 6.8 mM during BPA titration (left) and change in the CD signal intensity of OU3 at 262 nm depending on BPA concentration (right).

OU4 Boc-$P_SM_SM_SM_RP_S$

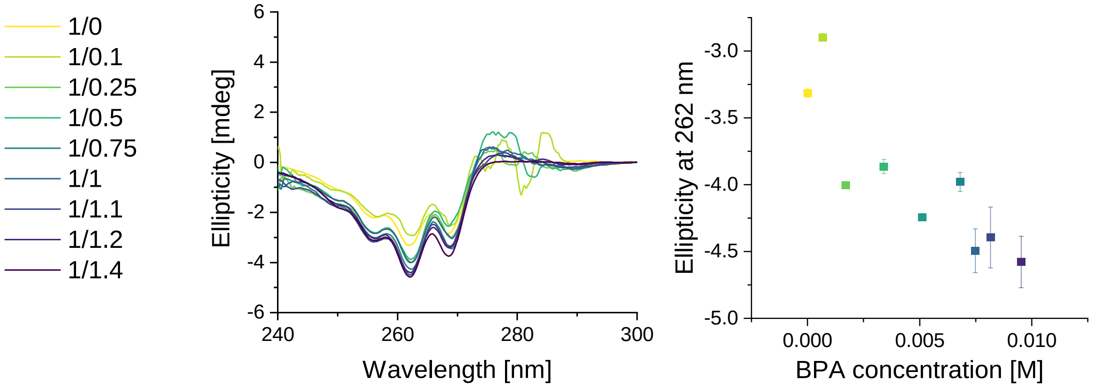

Figure S46. CD spectra of OU4 in $CHCl_3$, 6.8 mM during BPA titration (left) and change in the CD signal intensity of OU4 at 262 nm depending on BPA concentration (right).

OU5 Boc-$P_SM_RM_RM_SP_S$

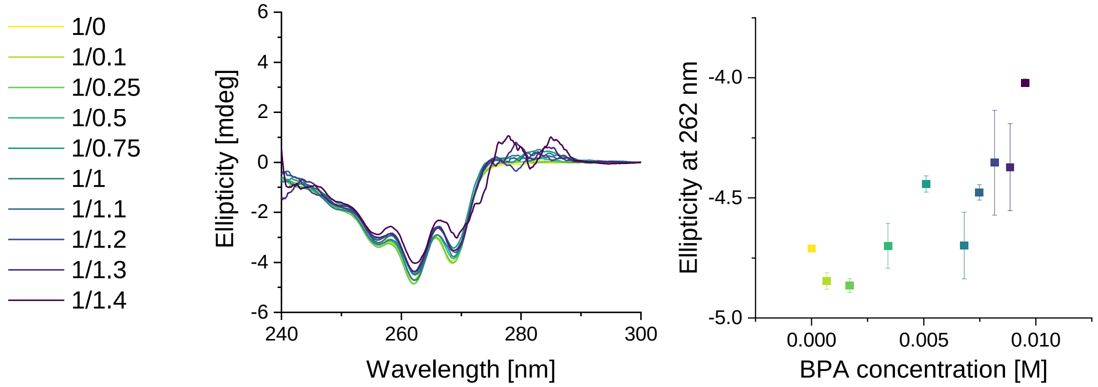

Figure S47. CD spectra of OU5 in $CHCl_3$ during BPA titration (left) and change in the CD signal intensity of OU5 at 262 nm depending on BPA concentration (right).

OU6 Boc-$P_SM_RM_SM_RP_S$

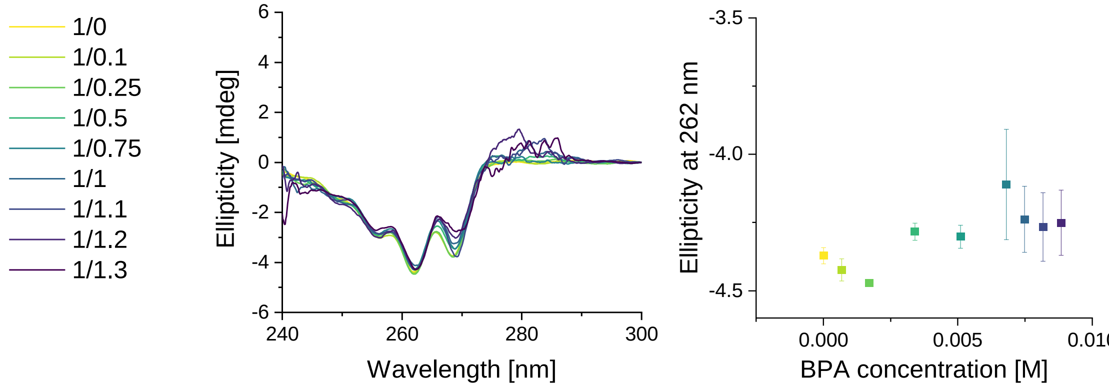

Figure S48. CD spectra of OU6 in $CHCl_3$ during BPA titration (left) and change in the CD signal intensity of OU6 at 262 nm depending on BPA concentration (right).

OU7 Boc-$P_SM_SM_RM_RP_S$

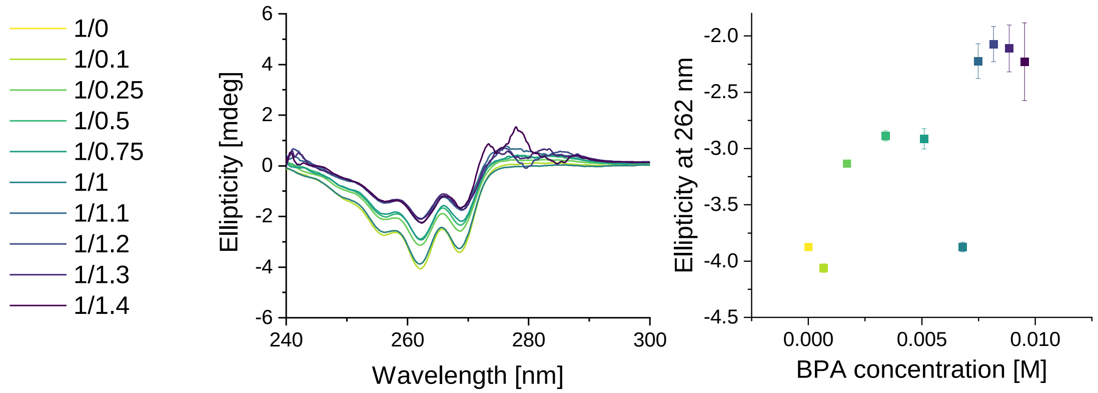

Figure S49. CD spectra of OU7 in $CHCl_3$ during BPA titration (left) and change in the CD signal intensity of OU7 at 262 nm depending on BPA concentration (right).

6.2. FLUORESCENCE ANALYSES

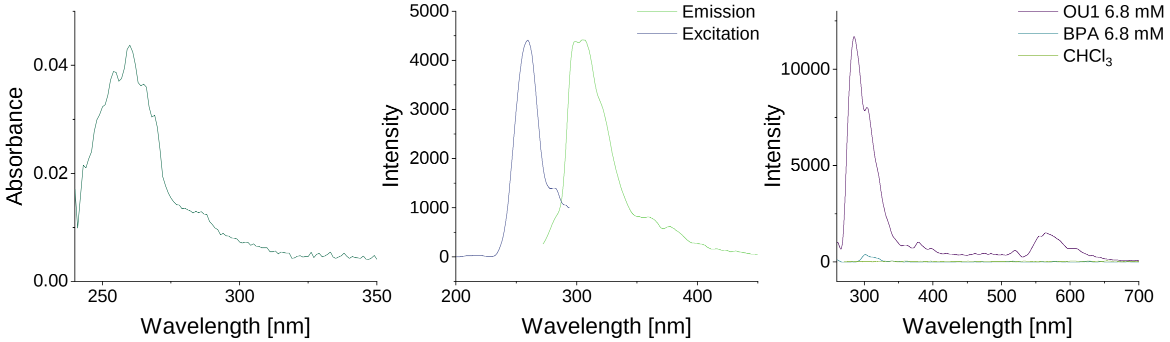

Figure S50. Absorbance measurement in $CHCl_3$ to select the appropriate concentration for fluorescence titration (A <0.05). UV-Vis (left); emission recorded at excitation wavelength 260 nm and excitation spectra of 68 μM OU1 (middle) and emission spectra of 6.8 mM of OU1 and BPA recorded at excitation wavelength 260 nm.

OU1 $Boc\text{-}P_SM_SM_SM_SP_S$

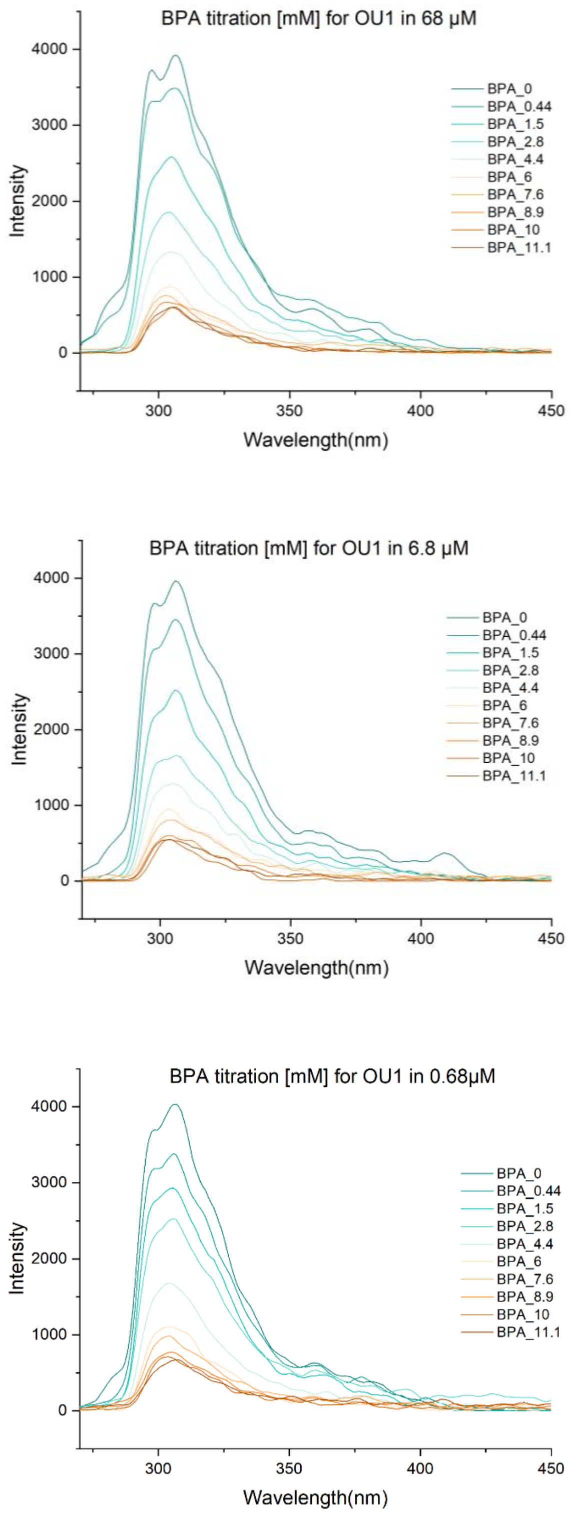

Figure S51. Changes in OU1 fluorescence spectra during BPA titration for different oligomer concentrations 68, 6.8, and 0.68 µM. Emission recorded at excitation wavelength 260 nm.

OU2 Boc-$P_SM_RM_SM_SP_S$

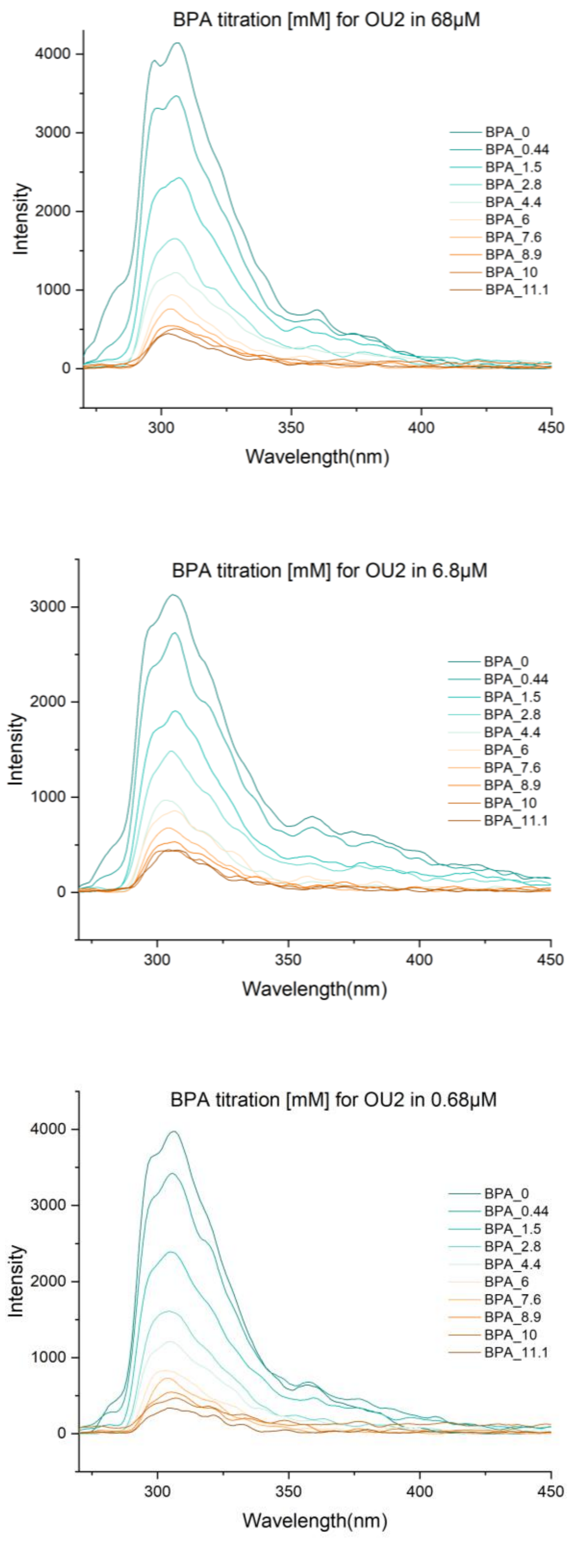

Figure S52. Changes in OU2 fluorescence spectra during BPA titration for different oligomer concentrations 68, 6.8, and 0.68 μM. Emission recorded at excitation wavelength 260 nm.

OU3 Boc-$P_{S}M_{S}M_{R}M_{S}P_{S}$

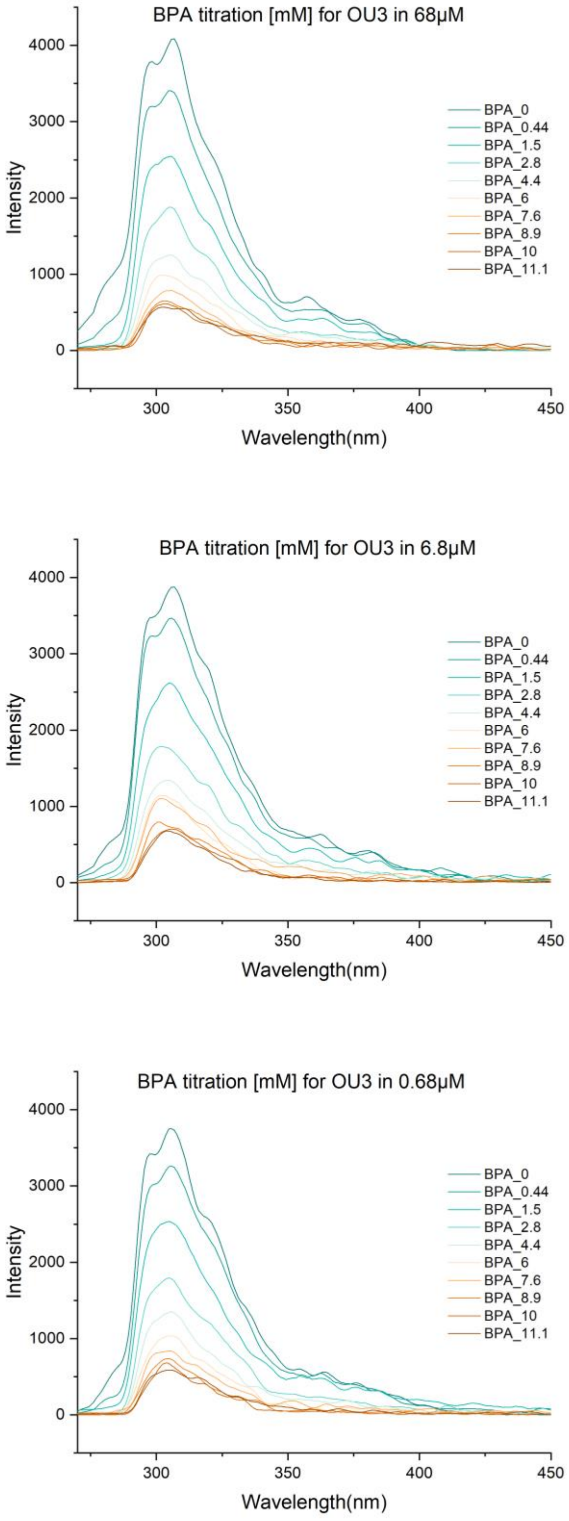

Figure S53. Changes in OU3 fluorescence spectra during BPA titration for different oligomer concentrations 68, 6.8, and 0.68 μM. Emission recorded at excitation wavelength 260 nm.

OU4 Boc-$P_SM_SM_SM_RP_S$

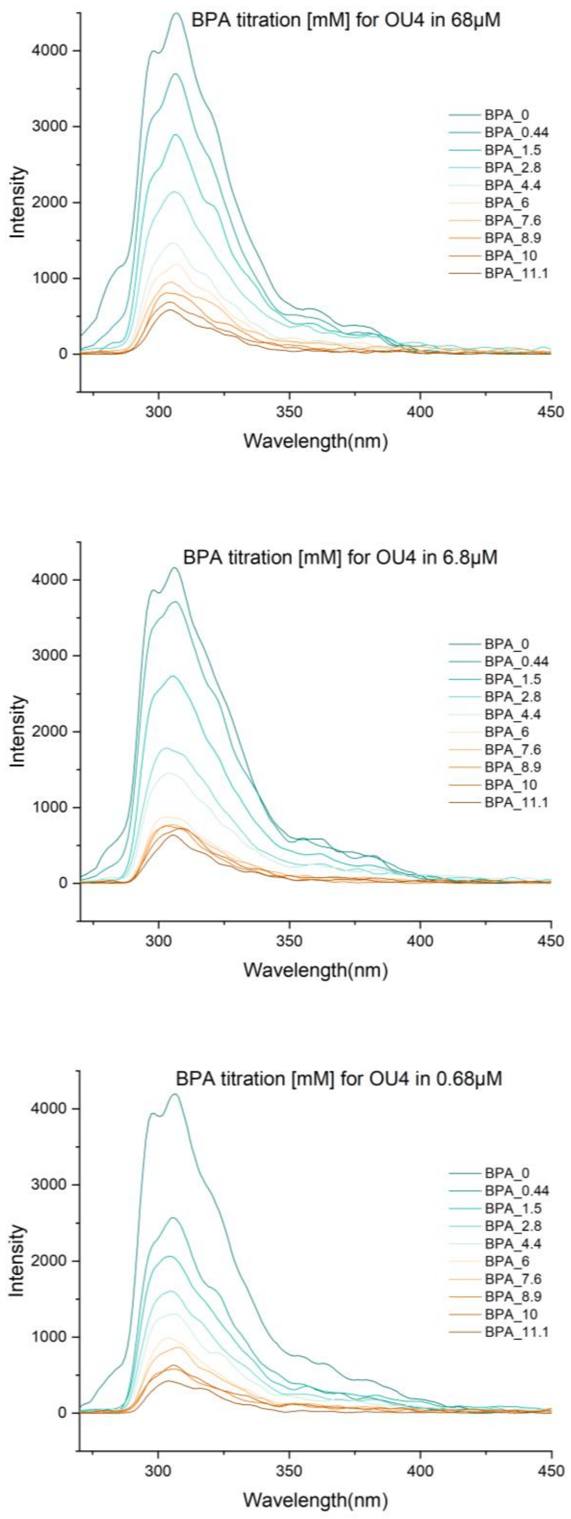

Figure S54. Changes in OU4 fluorescence spectra during BPA titration for different oligomer concentrations 68, 6.8, and 0.68 μM. Emission recorded at excitation wavelength 260 nm.

OU5 Boc-$P_SM_RM_RM_SP_S$

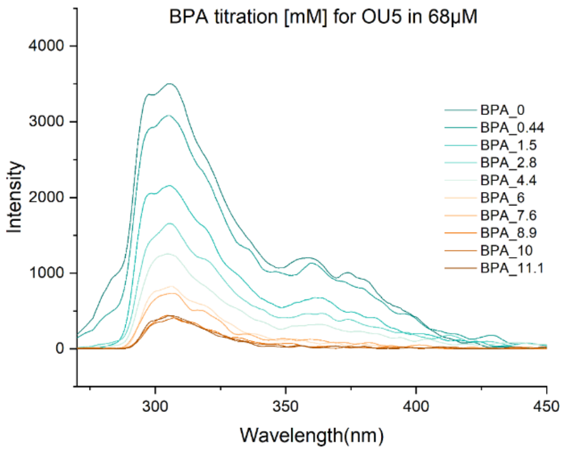

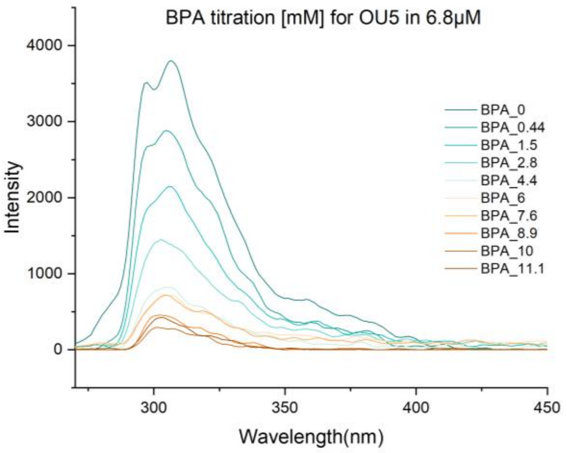

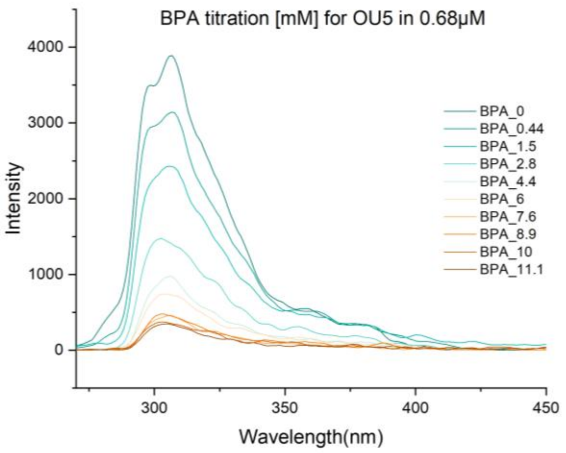

Figure S55. Changes in OU5 fluorescence spectra during BPA titration for different oligomer concentrations 68, 6.8, and 0.68 μM. Emission recorded at excitation wavelength 260 nm.

OU6 Boc-$P_{S}M_{R}M_{S}M_{R}P_{S}$

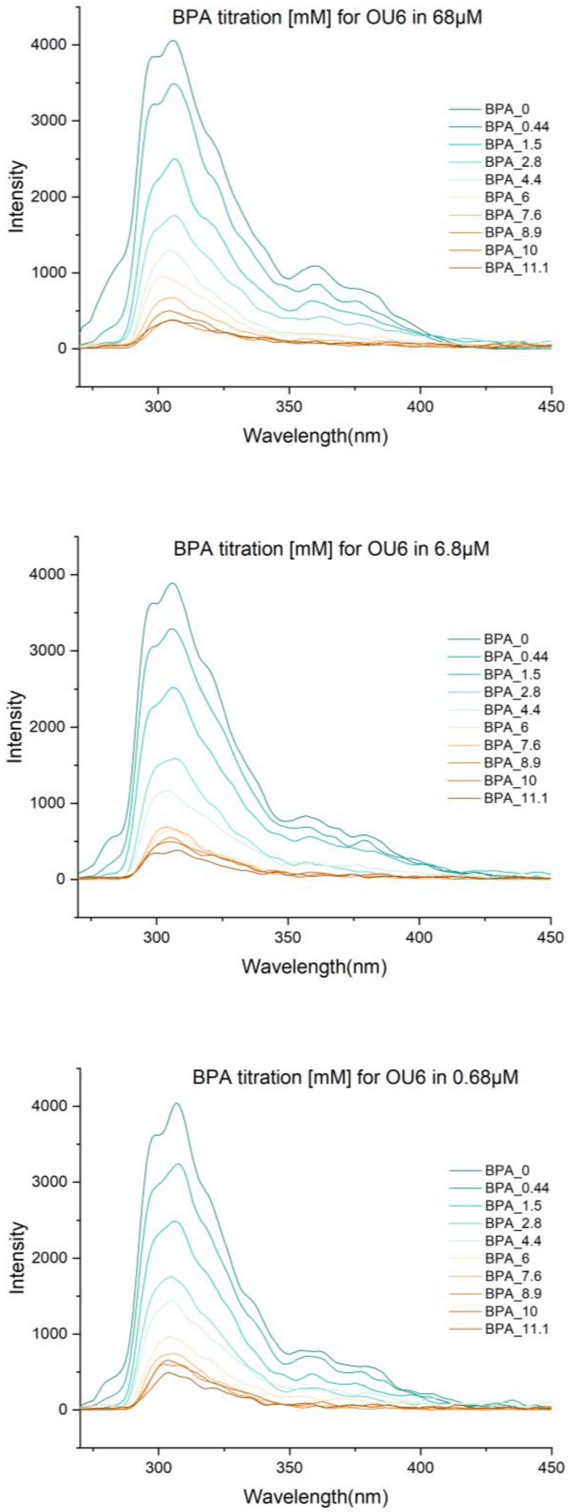

Figure S56. Changes in OU6 fluorescence spectra during BPA titration for different oligomer concentrations 68, 6.8, and 0.68 μM. Emission recorded at excitation wavelength 260 nm.

OU7 Boc-$P_SM_SM_RM_RP_S$

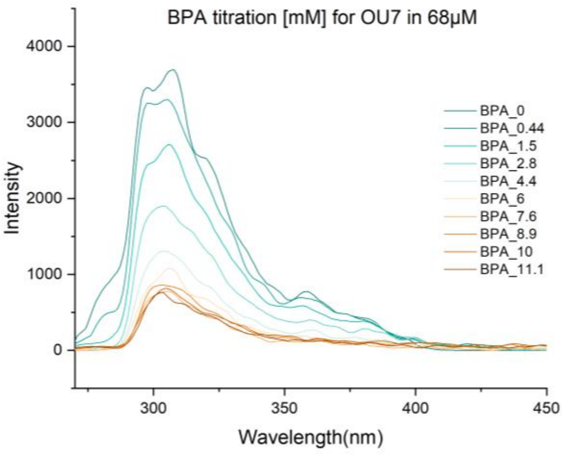

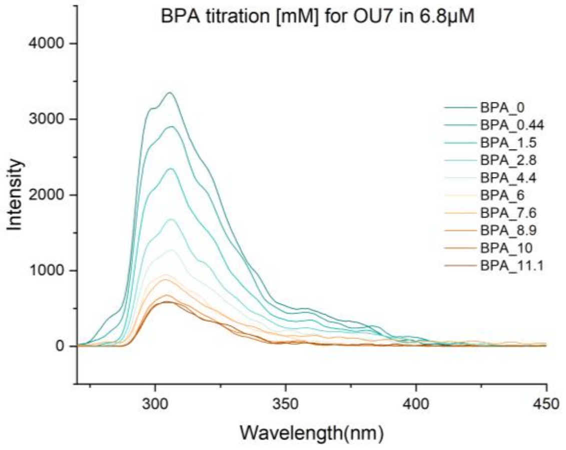

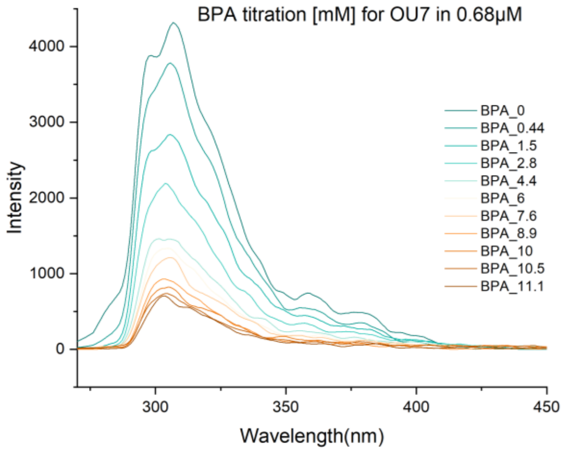

Figure S57. Changes in OU7 fluorescence spectra during BPA titration for different oligomer concentrations 68, 6.8, and 0.68 μM. Emission recorded at excitation wavelength 260 nm.

6.3. CHARACTERIZATION OF OLIGOMER-BPA BINDING

Table S6. Average K_a, K_d and ΔG_{bind} values for each oligomer calculated based on fluorescence quenching using the Sten-Volmer equation.

Oligomer-BPA complex	[a]$K_a \cdot 10^{-3}$ (M^{-1})	[b]K_d (M)	[c]ΔG_{bind}
**OU1** **(Boc-$P_SM_SM_SM_SP_S$)**	0.908	0.0011	-3.96556
**OU2** **(Boc-$P_SM_RM_SM_SP_S$)**	1.507	0.00066	-4.26054
**OU3** **(Boc-$P_SM_SM_RM_SP_S$)**	0.847	0.00118	-3.92489
**OU4** **(Boc-$P_SM_SM_SM_RP_S$)**	1.043	0.00096	-4.04617
**OU5** **(Boc-$P_SM_RM_RM_SP_S$)**	1.899	0.00053	-4.39418
**OU6** **(Boc-$P_SM_RM_SM_RP_S$)**	1.464	0.000683	-4.24368
**OU7** **(Boc-$P_SM_SM_RM_RP_S$)**	0.696	0.00144	-3.8106

[a]Association constant K_a values were calculated form $K_a=1/K_d$

[b]Dissociation constant K_d values were calculated as an average for three concentrations of oligomer $6.8 \cdot 10^{-5}$, $6.8 \cdot 10^{-6}$, $6.8 \cdot 10^{-7}$ M using formula:

$$\log\left(\frac{F_0-F}{F}\right) = \log\frac{1}{K_d} + n\log Q$$

[c]Binding energy ΔG_{bind} of oligourethane-BPA is determined using formula:

$$\Delta G_{bind} = -RT\ln\left(\frac{1}{K_d}\right)$$

7. REFERENCES

(1) Cwynar, P.; Pasikowski, P.; Szweda, R. One-Pot Approach for Multi-Step, Iterative Synthesis of Sequence-Defined Oligocarbamates. *Eur Polym J* **2023**, *182*, 111706. https://doi.org/10.1016/j.eurpolymj.2022.111706.

(2) Forysiak, W.; Kozub, S.; John, Ł.; Szweda, R. Discrete Oligourethanes of Sequence-Regulated Properties – Impact of Stereocontrol. *Polym Chem* **2022**, *13* (20), 2980–2987. https://doi.org/10.1039/D2PY00299J.

(3) Hunter, A. D. ACD/ChemSketch 1.0 (Freeware); ACD/ChemSketch 2.0 and Its Tautomers, Dictionary, and 3D Plug-Ins; ACD/HNMR 2.0; ACD/CNMR 2.0. *J Chem Educ* **1997**, *74* (8), 905. https://doi.org/10.1021/ED074P905.

(4) O'Boyle, N. M.; Banck, M.; James, C. A.; Morley, C.; Vandermeersch, T.; Hutchison, G. R. Open Babel: An Open Chemical Toolbox. *J Cheminform 2011 3:1* **2011**, *3* (1), 1–14. https://doi.org/10.1186/1758-2946-3-33.

(5) Revision C.01, M. J. Frisch, G. W. Trucks, H. B. Schlegel, G. E. Scuseria, M. A. Robb, J. R. Cheeseman, G. Scalmani, V. Barone, G. A. Petersson, H. Nakatsuji, X. Li, M. Caricato, A. V. Marenich, J. Bloino, B. G. Janesko, R. Gomperts, B. Mennucci, H. P. Hr, W. C. Gaussian 2016. **2016**.

(6) Sousa Da Silva, A. W.; Vranken, W. F. ACPYPE - AnteChamber PYthon Parser InterfacE. *BMC Res Notes* **2012**, *5*. https://doi.org/10.1186/1756-0500-5-367.

(7) Mulliken, R. S. Electronic Population Analysis on LCAO–MO Molecular Wave Functions. I. *J Chem Phys* **2004**, *23* (10), 1833. https://doi.org/10.1063/1.1740588.

(8) Tian, C.; Kasavajhala, K.; Belfon, K. A. A.; Raguette, L.; Huang, H.; Migues, A. N.; Bickel, J.; Wang, Y.; Pincay, J.; Wu, Q.; Simmerling, C. Ff19SB: Amino-Acid-Specific Protein Backbone Parameters Trained against Quantum Mechanics Energy Surfaces in Solution. *J Chem Theory Comput* **2020**, *16* (1), 528–552. https://doi.org/10.1021/ACS.JCTC.9B00591/SUPPL_FILE/CT9B00591_SI_002.ZIP.

(9) Lindahl; Abraham; Hess; Spoel, van der. GROMACS 2021.3 Manual. **2021**. https://doi.org/10.5281/ZENODO.5053220.

(10) Duan, Y.; Wu, C.; Chowdhury, S.; Lee, M. C.; Xiong, G.; Zhang, W.; Yang, R.; Cieplak, P.; Luo, R.; Lee, T.; Caldwell, J.; Wang, J.; Kollman, P. A Point-Charge Force Field for Molecular Mechanics Simulations of Proteins Based on Condensed-Phase Quantum

Mechanical Calculations. *J Comput Chem* **2003**, *24* (16), 1999–2012. https://doi.org/10.1002/JCC.10349.

(11) Daura, X.; Gademann, K.; Jaun, B.; Seebach, D.; van Gunsteren, W. F.; Mark, A. E.; Rigault, A.; Siegel, J.; Harrowfield, J.; Chevrier, B.; Moras, D.; Lehn, J.; Garrett, M.; Koert, U.; Meyer, D.; Fischer, J. Peptide Folding: When Simulation Meets Experiment. *Angew Chem Int Ed* **1998**, *31*, 1387–1404. https://doi.org/10.1002/(SICI)1521-3773(19990115)38:1/2.

(12) Waskom, M. L. Seaborn: Statistical Data Visualization. *J Open Source Softw* **2021**, *6* (60), 3021. https://doi.org/10.21105/JOSS.03021.

(13) Wang, J.; Wolf, R. M.; Caldwell, J. W.; Kollman, P. A.; Case, D. A. Development and Testing of a General Amber Force Field. *J Comput Chem* **2004**, *25* (9), 1157–1174. https://doi.org/10.1002/JCC.20035.

(14) Németh, L. J.; Hegedüs, Z.; Martinek, T. A. Predicting Order and Disorder for β-Peptide Foldamers in Water. *J Chem Inf Model* **2014**, *54* (10), 2776–2783. https://doi.org/10.1021/CI5003476.

(15) Valdés-Tresanco, M. S.; Valdés-Tresanco, M. E.; Valiente, P. A.; Moreno, E. Gmx_MMPBSA: A New Tool to Perform End-State Free Energy Calculations with GROMACS. *J Chem Theory Comput* **2021**, *17* (10), 6281–6291. https://doi.org/10.1021/ACS.JCTC.1C00645/ASSET/IMAGES/LARGE/CT1C00645_0005.JPEG.
